# Dysregulation of miRNAs has broad impacts on virus infection in *Drosophila*

**DOI:** 10.1128/jvi.00850-26

**Published:** 2026-07-02

**Authors:** Tyson G. Thomson, Daniel Chew, Karyn N. Johnson

**Affiliations:** 1 School of the Environment, The University of Queensland 1974, Brisbane, Queensland, Australia; Wageningen University & Research, Wageningen, Netherlands

**Keywords:** virus infection, microRNAs, functional screen, Drosophila C virus, Flock House virus, Sindbis virus, host-virus interactions, *Drosophila melanogaster*

## Abstract

Animal microRNAs (miRNAs) are important post-transcriptional regulators of gene expression. While the impact of certain miRNAs on viruses is well established, the frequency and magnitude of impacts more broadly are largely unknown. To address this, we screened the functional impact that the dysregulation of 34 individual miRNAs had on virus infection using a panel of three distinct RNA viruses: Drosophila C virus (*Dicistroviridae*), Flock House virus (*Nodaviridae*), and the arbovirus Sindbis virus (*Togaviridae*). We found that most of the 34 miRNA deletion mutant lines impacted infection of at least one virus, with close to half of the miRNA mutants impacting two or more. A wide variability and diversity in impacts on virus-induced mortality and viral RNA accumulation were also identified. Of those mutants that impacted the virus, the majority increased virus-induced mortality. Taken together, these results show that animal miRNAs play a broad and variable role in the dynamics of virus infection.

**IMPORTANCE:**

A clear understanding of how host and virus interact is fundamental in developing techniques to reduce virus infection. Animal microRNAs (miRNAs) are a group of genetic regulators that have been shown to impact host and virus interactions. However, the frequency and magnitude to which miRNAs impact virus infection is still largely unknown. Here, we used a randomly selected panel of miRNA mutant *Drosophila melanogaster* fly populations to identify how often and variably miRNAs influence virus infection. Our findings reveal that a high proportion of miRNA mutants impacted virus, with close to half of them impacting multiple viruses. We also found a wide range of miRNA impacts on virus-induced mortality and viral RNA accumulation. Taken together, our results suggest that miRNAs play a common and variable role in the outcome of virus infection.

## INTRODUCTION

Animal microRNAs (miRNAs) are small 22-nucleotide RNAs that modulate the post-transcriptional regulation of gene expression (1). miRNAs are well established to impact virtually all biological functions, including cell growth, development, metabolism, and immunity (2–6). miRNAs are biosynthesized from precursor-miRNA transcripts through a canonical miRNA pathway, which produces a mature miRNA duplex (7, 8). Mature miRNAs are loaded into silencing complexes, where they induce translational repression and degradation of partially complementary messenger RNA (mRNA) transcripts (9, 10). As such, single miRNAs can target many genes, and many miRNAs may target a single gene. miRNAs are diverse gene regulators, each with distinct targets, expression profiles, and subsequent roles within the host (11–13).

The influence of host miRNAs on virus infection has been demonstrated across the animal kingdom, including in humans and mosquitoes (14, 15). miRNAs may impact virus directly, through the targeting of viral RNA or immune pathways (16, 17), or indirectly, by modulating relevant host factors such as cellular environment and metabolism (3, 15). miRNAs have the capacity to influence the speed and efficiency of virus infection, either promoting or preventing replication (16, 18, 19). Additionally, miRNAs may influence the ability of hosts to tolerate or resist the consequences of infection (20, 21). Different viruses are diverse, varying substantially in tropism, infection strategy, and pathogenicity (4). As such, the ways that host miRNAs interact with viruses are also diverse (20). Virus infection itself has been shown to alter host miRNA expression, although the specific mechanisms that underpin this are largely unclear (22–25). Overall, the outcome of infection is the product of all these complex interactions.

Current research focuses primarily on individual miRNA-virus-host interactions in isolation, mapping their impacts on host organisms, roles in virus infection, and mechanisms of action (17, 23, 26, 27). Consequently, limited information exists regarding the frequency and magnitude of functional impacts that miRNAs have on virus infection (28). Functional impact is defined as the total impact that individual miRNAs have, made up of many individual miRNA-mRNA interactions: with consequences positive, negative, neutral, direct, and indirect on the outcome of virus infection.

Better understanding the interplay between miRNA and virus will provide important context for the overall relationship between host and virus. We sought to understand the impact of miRNAs in the context of viral infection and whether these impacts varied between diverse viruses. To do this, we screened the functional impact that 34 individual miRNA deletions had on infection with a panel of three viruses from distinct RNA virus families.

## RESULTS

### The majority of miRNA mutant lines impact virus infection

**Fig 1:**
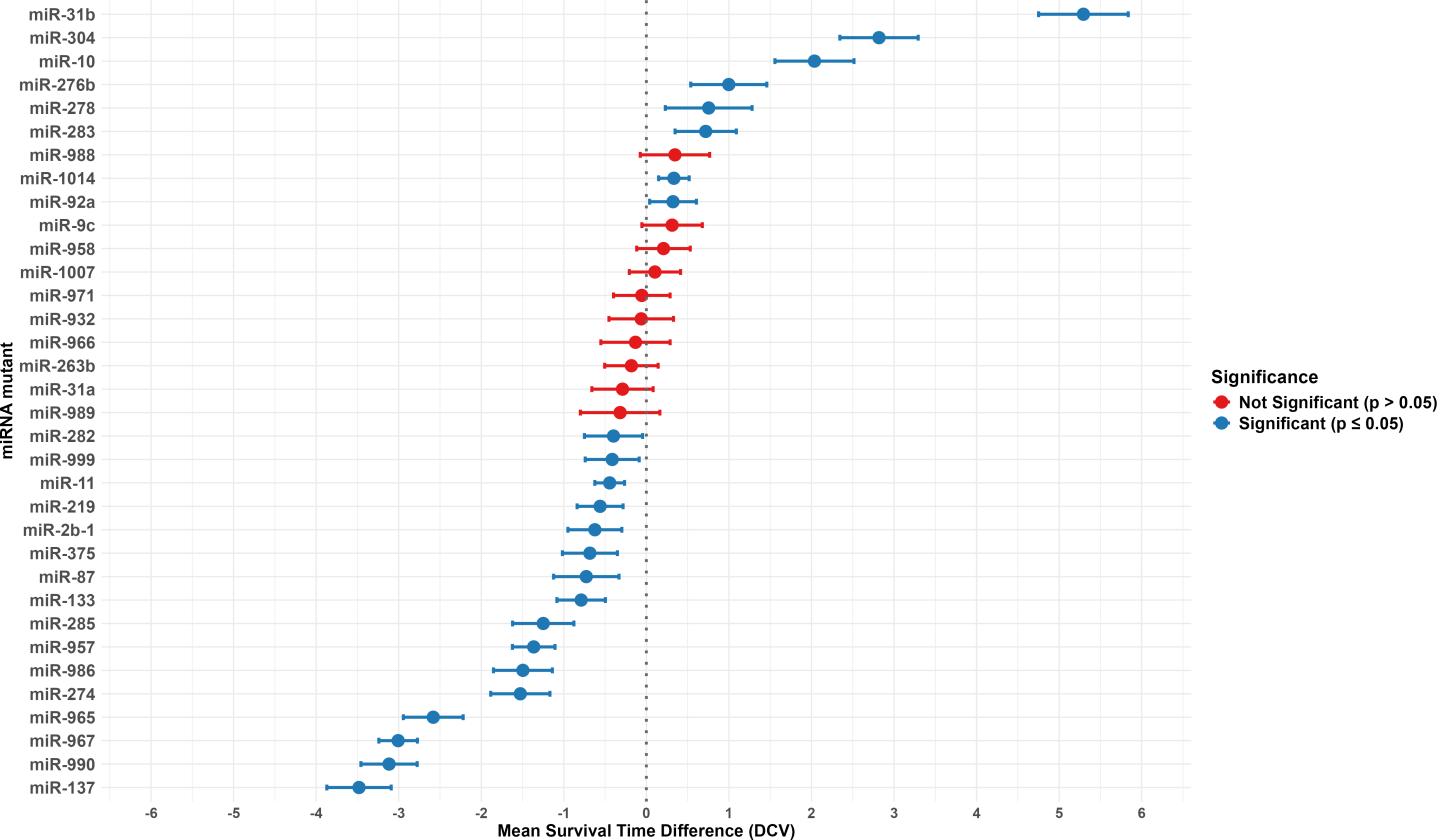
miRNA dysregulation variably impacts DCV-induced mean survival time. Each row on the *y*-axis indicates a distinct *D. melanogaster* miRNA mutant line (e.g., miR-31b, indicates a line with loss of miR-31b gene). The *x*-axis indicates the restricted mean survival time (RMST) difference of the mutant line compared to control, upon DCV-induced mortality. RMST difference and statistical analyses were conducted using the *rmst2* package in R. Each dot indicates the approximate RMST difference, and the tails indicate 95% confidence intervals. The dotted vertical line indicates an RMST difference of 0—that is, no functional impact of miRNA loss on survival vs control. Blue dots indicate a statistically significant difference (*P* ≤ 0.05), while red indicates no significance (*P* > 0.05). A negative RMST difference can be interpreted as days decrease in survival, while a positive RMST difference as days increase in survival vs control. To assist with interpretation, miR-31b has an RMST difference of 5.3; thus, on average, flies of the miR-31b loss-of-function line lived 5.3 days longer than control. Each datapoint consists of at least three independent biological replicates. Survival curves for each individual miRNA mutant are provided in Fig. S1, and statistical outputs are provided in Table S1.

**Fig 2:**
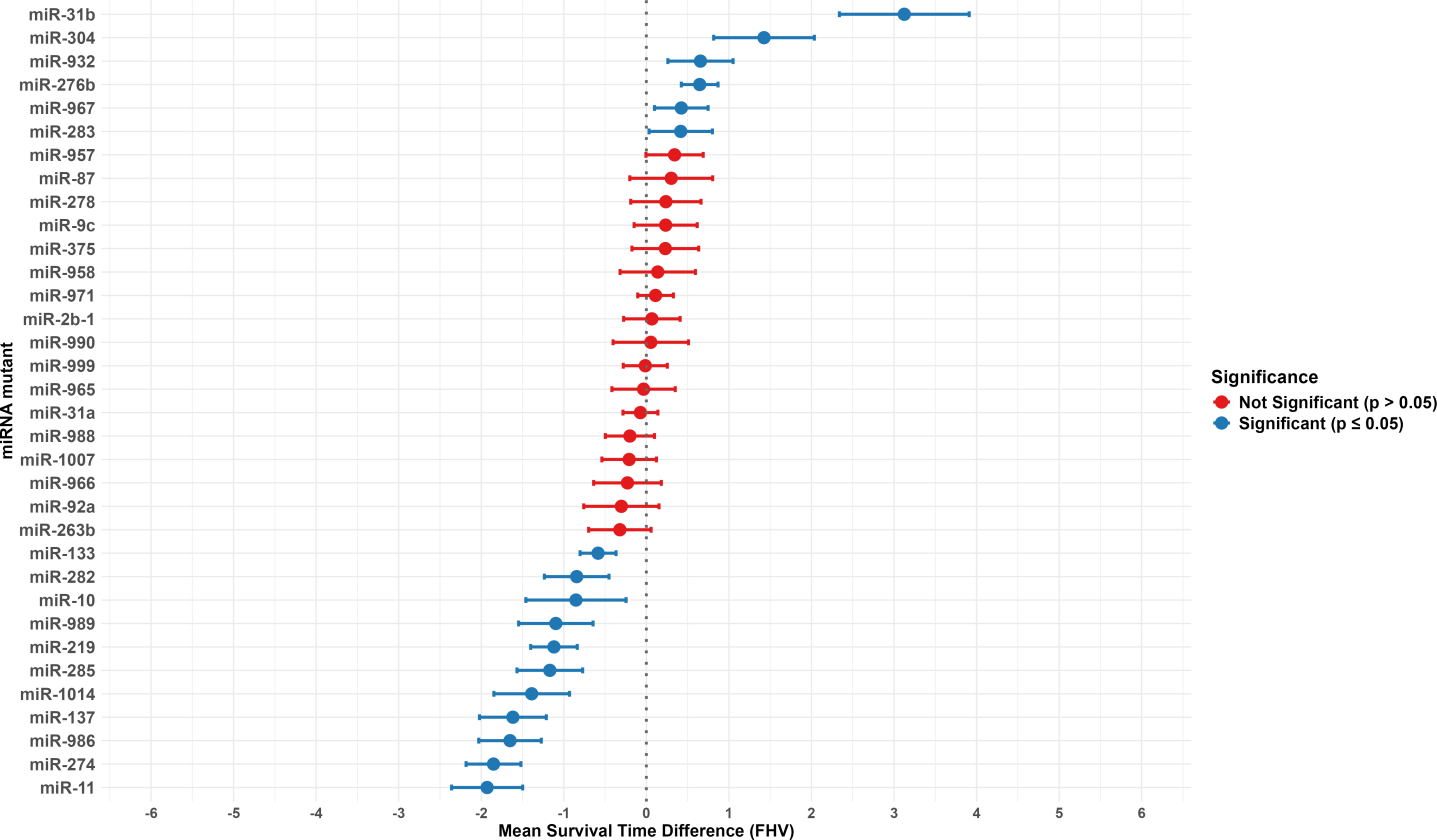
miRNA dysregulation variably impacts FHV-induced mean survival time. Each row on the *y*-axis indicates a distinct *D. melanogaster* miRNA mutant line (e.g. miR-31b, indicates a line with loss of gene miR-31b). The x-axis indicates the restricted mean survival time (RMST) difference of the mutant line compared to control, upon FHV-induced mortality. RMST difference and statistical analyses were conducted using the *rmst2* package in R. Each dot indicates the approximate RMST difference, and the tails indicate 95% confidence intervals. The dotted vertical line indicates an RMST difference of 0 — that is, no functional impact of miRNA loss on survival vs control. Blue dots indicate a statistically significant difference (*P* ≤ 0.05), while red indicates no significance (*P* > 0.05). A negative RMST difference can be interpreted as days decrease in survival, while a positive RMST difference as days increase in survival vs control. To assist with interpretation, miR-31b has an RMST difference of 3.1; thus, on average, flies of the miR-31b loss-of-function line lived 3.1 days longer than control. Each datapoint consists of at least three independent biological replicates. Survival curves for each individual miRNA mutant are provided in Fig. S2, and statistical outputs are provided in Table S2.

To systematically identify the impact of miRNA dysregulation on virus infection, we screened a randomly selected panel of 34 miRNA loss-of-function fly lines with three distinct viruses: Drosophila C virus (DCV, *Dicistroviridae*), Flock House virus (FHV, *Nodaviridae*), and Sindbis virus (SINV, *Togaviridae*). For this screen, we infected more than 13,000 adult *Drosophila melanogaster* flies and scored over 400 independent biological replicates. For DCV and FHV, virus-induced mortality was measured by scoring survival daily, following virus challenge. Survival analyses were conducted using restricted mean survival time (RMST) as a summary measure of each miRNA loss-of-function line, vs control. For DCV, the screen identified that 71% (24/34) of the miRNA mutant lines tested showed a statistically significant impact on virus-induced mortality (RMST, *P* ≤ 0.05; Fig. 1; Table S1). Of those impacting DCV outcome, there was a mean increase of 1.7 days, ranging from 0.3 to 5.3 days, and a mean decrease of 1.4 days, ranging from 0.4 to 3.5 days. For FHV, the screen identified that 50% (17/34) of the miRNA mutant panel had an impact on virus-induced mortality (RMST, *P* ≤ 0.05; Fig. 2; Table S2). Of those lines impacting FHV outcome, there was a mean increase of 1.1 days, ranging from 0.4 to 3.1 days, and a mean decrease of 1.3 days, ranging from 0.6 to 1.9 days.

**Fig 3:**
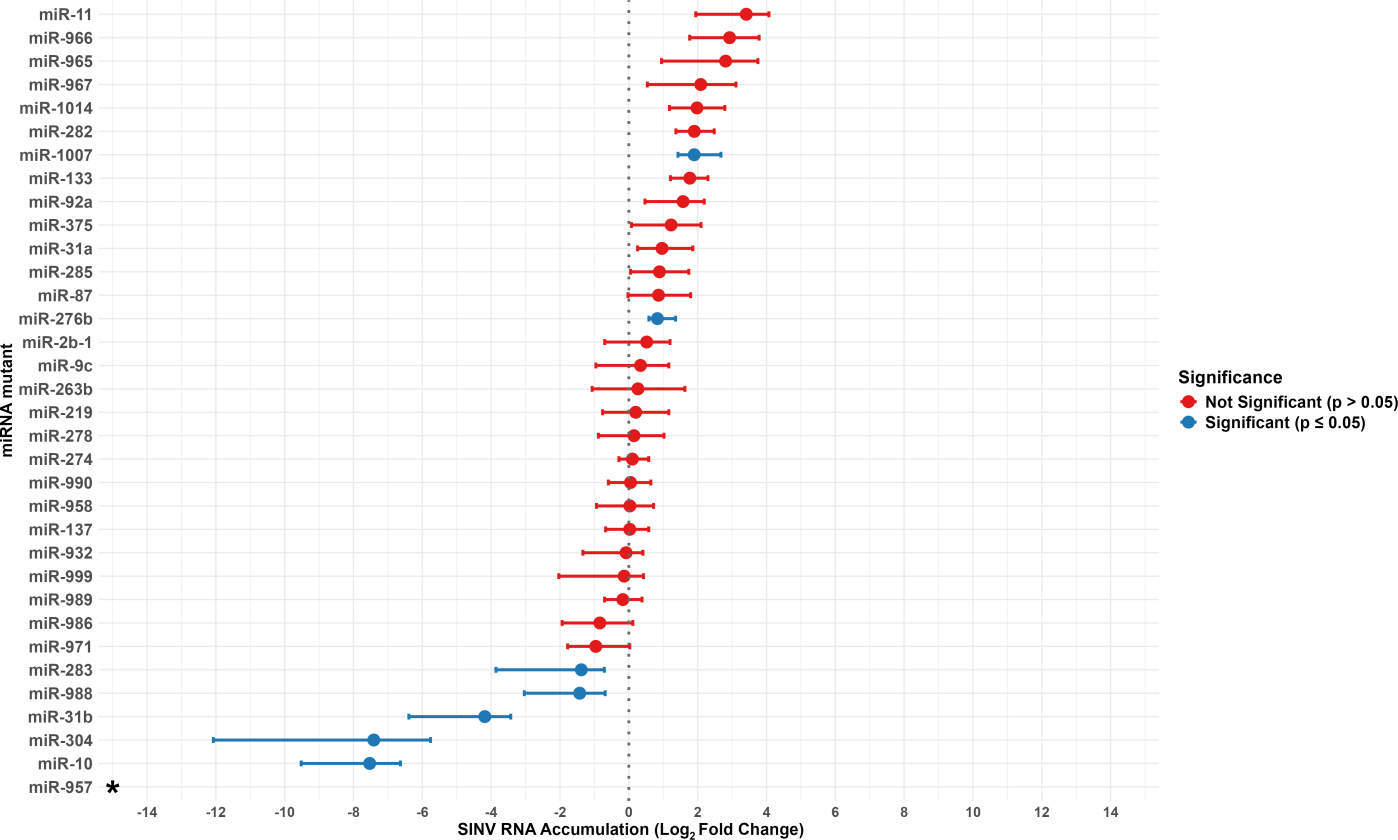
miRNA dysregulation variably impacts SINV RNA accumulation. Each row on the *y*-axis indicates a distinct miRNA mutant line (e.g., miR-31b, indicates a line with loss of gene miR-31b). The *x*-axis indicates the log_2_ fold change (FC) difference in viral RNA levels at 3 days post-infection, for the miRNA mutant line compared to control. Each dot indicates the mean log_2_FC, while the tails indicate 95% confidence intervals. The dotted vertical line indicates a log_2_FC difference of 0— that is, no difference from control. Statistical analyses were conducted using a paired two-tailed *t*-test, with Benjamini-Hochberg correction in R. Blue dots indicate a statistically significant change in mean normalized expression (*P* ≤ 0.05), while red indicates no significance (*P* > 0.05). * Indicates miRNA mutant line that had no detectable SINV signal. To assist with interpretation, miR-10 had a log_2_FC of −7.5, which is equivalent to a decrease of 99.5%; miR-1007 had a log_2_FC of 1.90, which is equivalent to an increase of 300%. Each datapoint consists of at least three independent biological replicates. Viral RNA was quantified by RT-qPCR, using subgenomic primers specific to the SINV E2 glycoprotein normalized to housekeeping gene *RpL32*. Statistical outputs are provided in Table S3.

For SINV, since arboviruses commonly do not cause mortality in their insect host, impact was quantified using viral RNA accumulation measured at 3 days post-infection (dpi). RNA accumulation analysis was conducted using mean normalized expression, comparing miRNA loss-of-function line and control. Although *D. melanogaster* is not a natural host of SINV, we identified that 24% (8/34) of the miRNA mutants tested impacted the accumulation of viral RNA (paired *t*-test, *P* ≤ 0.05; Fig. 3; Table S3). Of those miRNAs that impacted SINV RNA accumulation, there was a mean viral RNA increase of 175%, ranging from 80% to 300%. Additionally, there was a mean decrease of 80%*, ranging from 60% to 99.5% (*excludes miR-957, which had SINV levels below the level of detection). Using this experimental design, we were unable to distinguish whether miR-957 being below the level of detection was due to greatly reduced viral RNA levels or elimination of virus.

**Fig 4:**
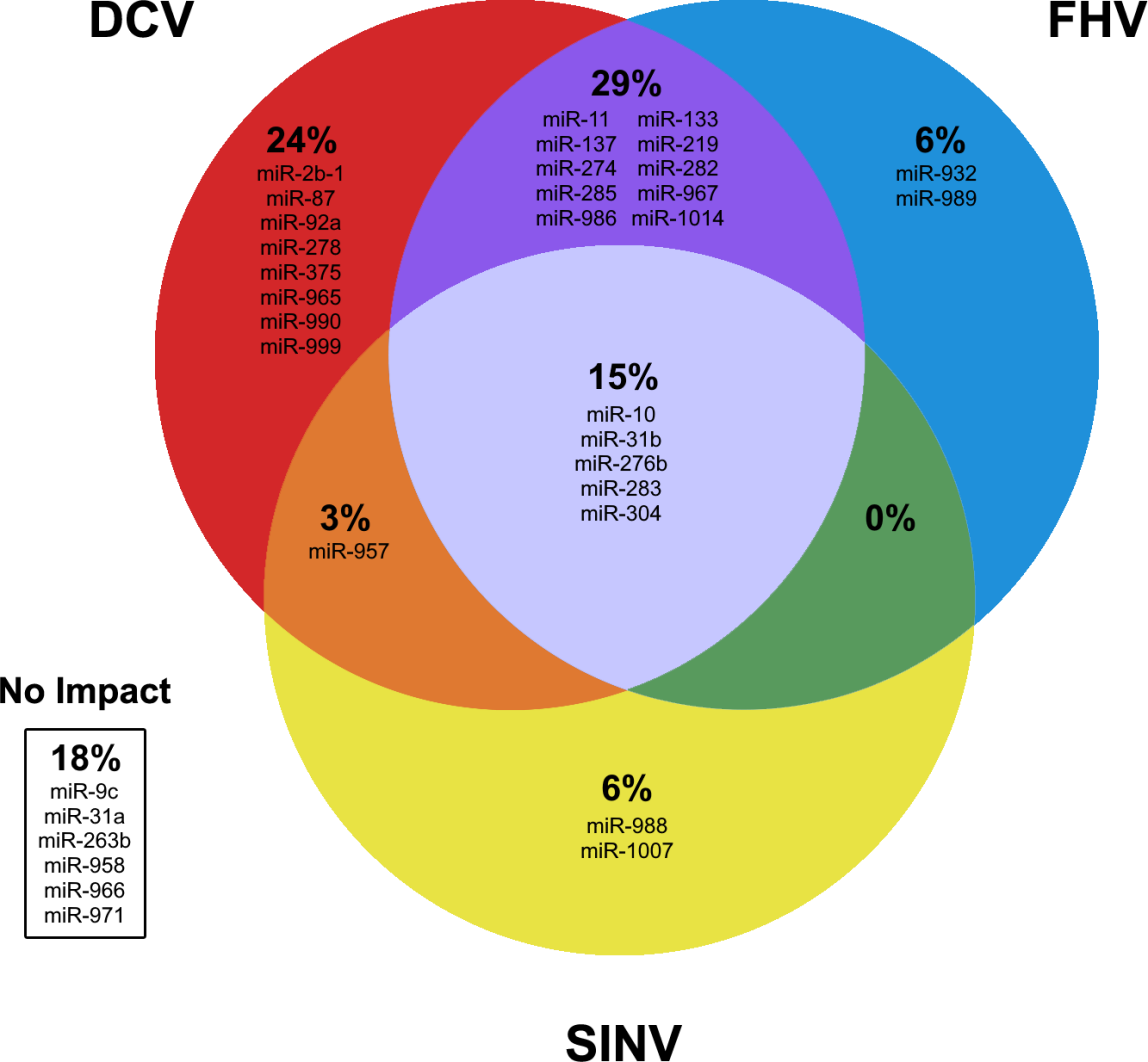
miRNA mutant lines differentially impact diverse viruses. A panel of 34 miRNA mutant lines was screened upon infection with each of DCV, FHV, and SINV. DCV and FHV were screened using survival bioassays and analyzed using RMST. SINV was quantified using mean normalized expression and analyzed with paired *t*-tests. A miRNA mutant line was considered to impact virus if it yielded a statistically significant change compared to control. Each colored section indicates a different combination of impacts: DCV only (red), FHV only (blue), SINV only (yellow), DCV-FHV (purple), DCV-SINV (orange), FHV-SINV (green), DCV-FHV-SINV (gray), and no impact (white). Due to whole number rounding, the above percentages sum to 101%.

To visualize the impact of each miRNA mutant line on virus infection, we compiled the screen results for DCV, FHV, and SINV into a summary graphic (Fig. 4). It is worth noting that SINV impact was quantified differently from DCV and FHV. Our screen identified that in total, 82% (28/34) of the miRNA mutants impacted at least one virus. Furthermore, 47% (16/34) of the lines impacted at least two viruses, and 15% (5/34) impacted all three viruses. Notably, only 18% (6/34) of the miRNA dysregulation lines had no quantifiable impact on any of the three viruses tested.

### miRNA mutant lines variably impact DCV and FHV RNA accumulation

**Fig 5:**
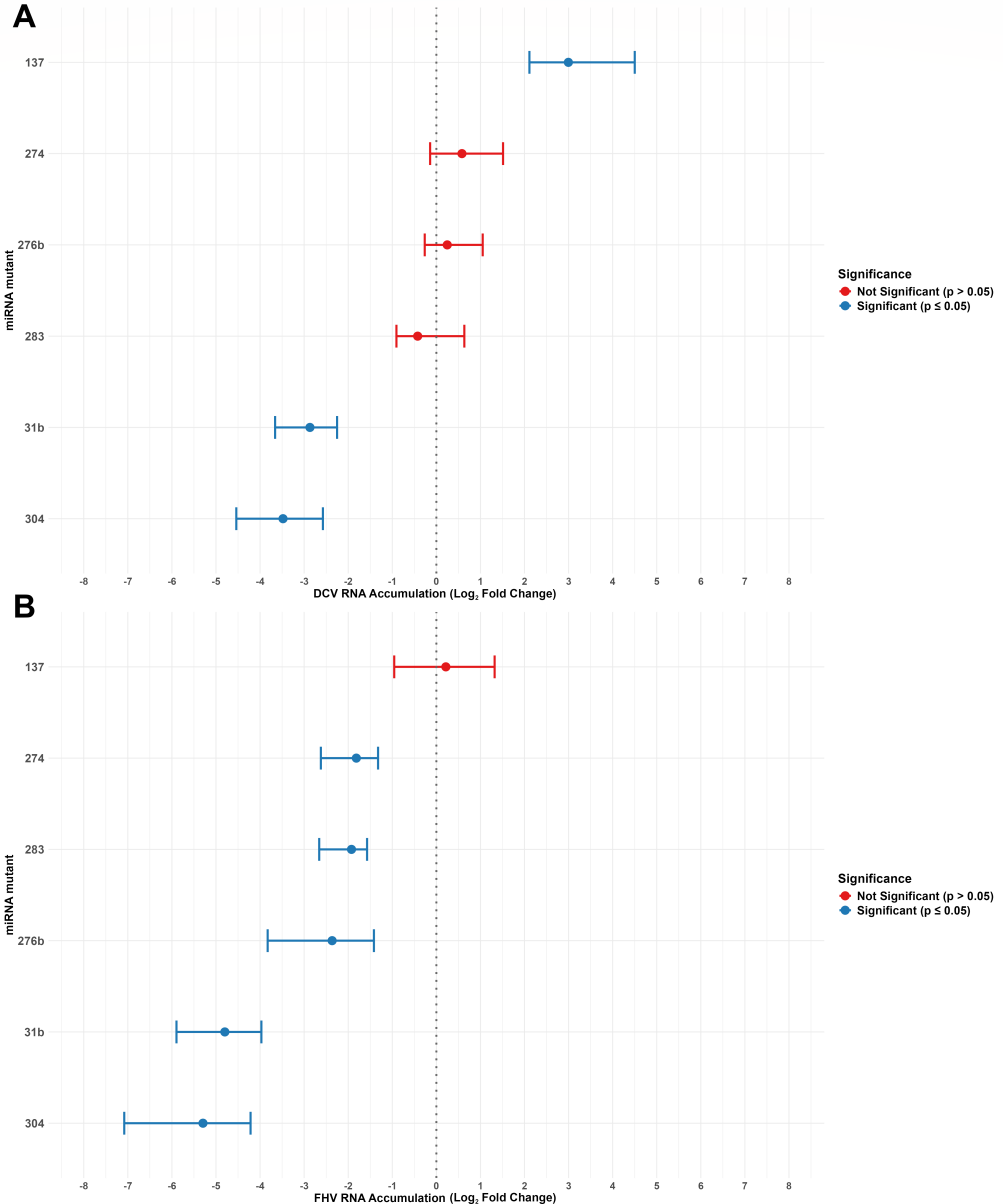
Subset of miRNA dysregulation lines variably impacts DCV and FHV RNA accumulation. Each row on the *y*-axis indicates a distinct miRNA mutant line. The *x*-axis indicates the log_2_FC difference in (**A**) DCV and (**B**) FHV RNA expression at 3 dpi, for the miRNA mutant line compared to control. Each dot indicates the mean log_2_FC, while the tails indicate 95% confidence intervals. The dotted vertical line indicates a log_2_FC difference of 0—that is, no difference from control. Statistical analyses were conducted using a paired two-tailed *t*-test in R. Blue dots indicate a statistically significant change in mean normalized expression (*P* ≤ 0.05), while red indicates no significance (*P* > 0.05). To assist with interpretation, miR-304 DCV had a log_2_FC of −3.5, which is equivalent to a decrease of 90%; miR-137 DCV had a log_2_FC of 3.0, which is equivalent to an increase of 700%. Each datapoint consists of at least three independent biological replicates. Viral RNA was quantified by RT-qPCR, using primers specific to either the DCV Replicase Polyprotein or Flock House virus hypothetical protein gp1, normalized to housekeeping gene *RpL32*. Outputs are provided in Tables S4 and S5.

To explore how the miRNA mutant lines impacted the accumulation dynamics of viruses other than SINV, DCV, and FHV viral RNA levels were quantified for a subset of the miRNA panel. Six miRNAs were selected for follow-up using two criteria: a high impact on DCV/FHV mortality, and/or a significant impact on all three viruses. For DCV, three of the six miRNA mutants had an impact on viral RNA accumulation at 3 dpi (paired *t*-test, *P* ≤ 0.05; Fig. 5A; Table S4). The miR-137 mutant increased DCV viral RNA accumulation by 700%, while miR-31b and miR-304 mutants each decreased it by 90%. For FHV, we identified that five of the six miRNA mutants impacted viral RNA levels at 3 dpi (paired *t*-test, *P* ≤ 0.05; Fig. 5B; Table S5). FHV levels were only reduced, ranging from a decrease of 70–98%.

### Mutants that impact DCV and FHV mainly increase mortality

To determine trends within the screen, the directionality of the functional impacts on mortality was contrasted. Given that mortality was quantified for DCV and FHV only, comparative analyses were performed for these two viruses. Of those miRNA mutants that impacted DCV, 67% (16/24) increased mortality, while 33% (8/24) decreased it (Fig. 1). The same distribution was observed for FHV, with 65% (11/17) of the impactful miRNAs increasing mortality, while 35% (6/17) decreased mortality (Fig. 2).

### Most mutants impacting DCV and FHV share the same directionality

**Fig 6:**
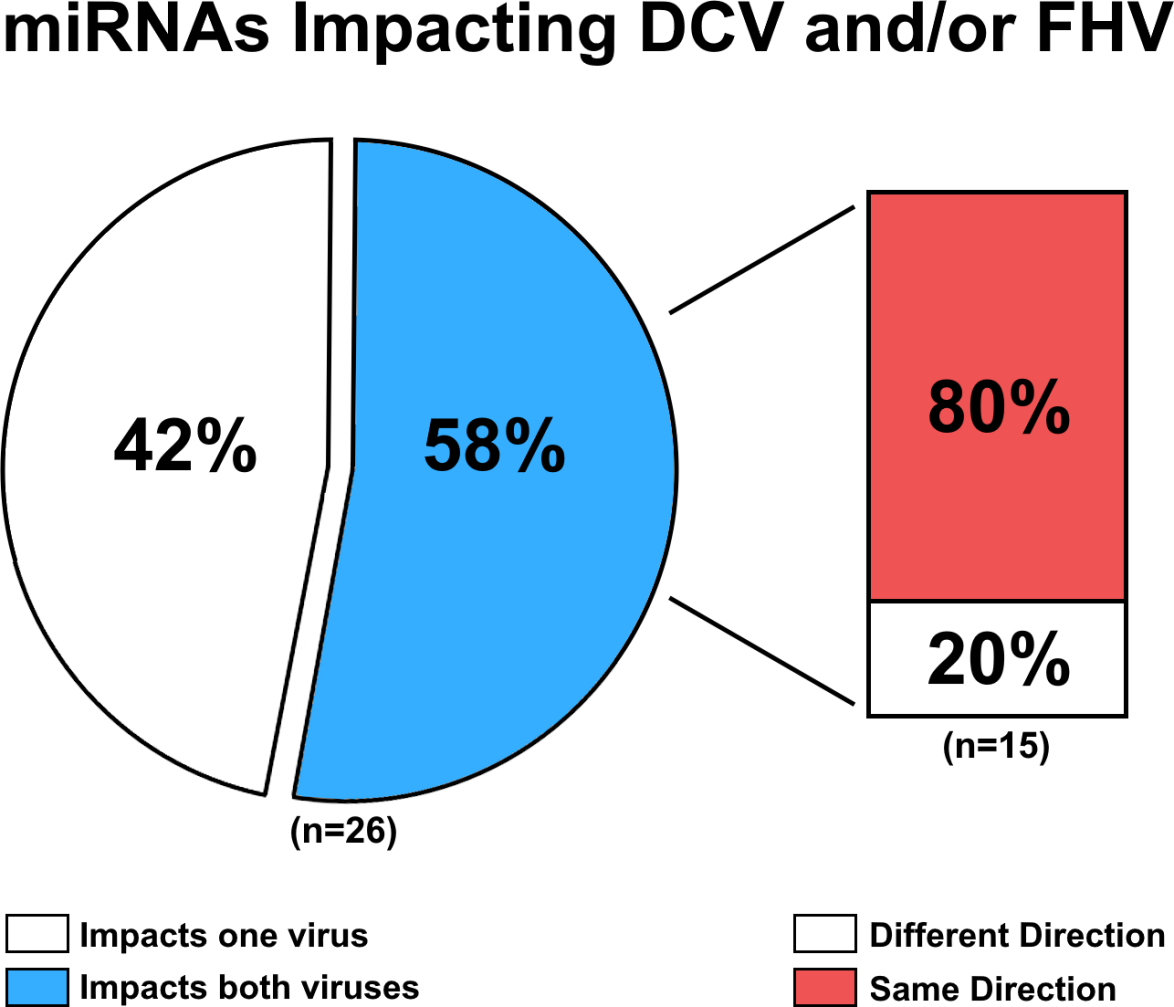
Over half of the miRNA mutant lines impacting DCV and FHV overlap. (Left) Pie chart comparing miRNA mutant lines that impacted one of either DCV or FHV, and those that impacted both viruses. (Right) Of the miRNAs that impacted DCV and FHV, the majority impacted both in the same direction. The directionality of impact refers to whether the miRNA mutant increased or decreased survival compared to paired control.

To analyze the similarities in miRNA functional impact on DCV and FHV infection, the overlap of miRNAs was considered. We identified that of the 26 miRNA lines that impact the outcome of infection with either DCV or FHV, 58% (15/26) impacted both viruses (Fig. 6). Of those miRNA lines that impacted both DCV- and FHV-induced mortality, 80% (12/15) impacted host mortality in the same direction (Fig. 6).

### No evidence for a link between miRNA-virus functional impact and miRNA conservation

The evolutionary conservation of mature miRNA sequences across different species and genera is well-established (29–31). Both the range and level of conservation vary greatly, with some miRNAs found only in *D. melanogaster*, while others are conserved across species as disparate as *D. melanogaster* and humans (31, 32). To evaluate the potential relationship between miRNA sequence conservation and functional impact on virus, we performed a series of conservation analyses. This analysis focused on conservation with three well-studied Dipterans: *Drosophila simulans*, *Aedes aegypti* (Yellow fever mosquito), and *Anopheles gambiae* (African malaria mosquito). We identified no clear relationship between miRNA conservation status and likelihood to impact virus infection (Output S2). Additionally, conserved miRNAs were no more likely to impact more/multiple viruses than those that were not conserved (Output S2).

## DISCUSSION

Animal miRNAs play a vital role in the regulation and fine-tuning of cellular processes and function (1, 32). This functionality extends to host-virus interactions, where miRNAs may influence how an organism responds to and battles infection (17, 23, 33, 34). Our screen identified that the overwhelming majority (82%) of miRNA mutants tested impacted at least one of the three viruses screened. Distinguishing by individual virus, we determined that a high proportion of mutant lines impacted infection of each (DCV: 71%, FHV: 50%, and SINV: 24%). Furthermore, we observed that close to half (47%) of the lines tested impacted at least two viruses. Taken together, our study utilized a randomly selected panel of miRNA mutant lines to establish that the majority of miRNA genes have a quantifiable impact on virus infection. This result is consistent with the consensus that individual miRNAs are diverse regulators, impacting many different genes and pathways (6, 12, 35). As such, it is logical that the dysregulation of many individual miRNAs would cause measurable impacts on virus infection, particularly when you consider that virus infection and replication rely intimately on the cellular environment (34, 36).

This functional screen utilized a panel of miRNA loss-of-function mutant fly lines originally generated in (37). This panel allowed the impact that individual miRNA gene absence had on virus infection to be quantified *in vivo*. miRNA genes generally encode for the biosynthesis of two complementary mature miRNA strands, each capable of silencing many downstream targets (12, 38). Therefore, the functional impact of a miRNA gene mutation includes the absence of both mature miRNA strands. The functional impact in this context refers to the sum or culmination of all interactions between host, virus, and miRNA, be they direct or indirect, positive, negative, or neutral. Therefore, the functional impact of a miRNA mutant on the outcome of virus infection could be the result of a single overpowering interaction, or the combination of many complex interactions. This functional screen was not designed to distinguish between individual mechanisms or interactions, instead focusing on the overall impact that the miRNA has on virus infection. This allows the screen to capture the holistic impact of host miRNAs on virus infection.

Conventionally, the impact of miRNAs on virus is studied in isolation, with a focus on individual mechanisms of action (17, 23, 26, 27). This screen presented a unique opportunity to directly compare and contrast the functional impacts that many miRNA mutants had on virus infection. We identified a wide range in impacts on survival for both DCV (ranging from +5.3 to −3.5 days) and FHV (from +3.1 to −1.9 days). This variability is consistent with the understanding that miRNAs themselves are diverse, variable, and contribute many individual interactions (39). Additionally, we identified a wider range of impact for DCV-infected lines, compared to FHV. One hypothesis that explains this difference is the natural disparity in how the host and virus interact between DCV and FHV, two viruses from distinct families (33). Consequently, DCV control flies experienced 100% mortality at 7.6 days on average, while FHV control flies experienced this at 8.8 days. Additionally, virus concentration inoculated (10^7^ infectious units per mL [IU/mL] for DCV and 10^8^ IU/mL for FHV) has also been shown to change accumulation dynamics within insect hosts (40). Finally, it is established that virus infection can modulate miRNA levels, with DCV and FHV having been shown to modulate the expression of many mature miRNAs tested (23, 25). Whether this pattern is consistent for other viruses, such as SINV, is largely unknown.

We have established that individual miRNA-virus interactions may contribute positively, negatively, or neutrally to virus infection, and that the functional screen observes the sum of these interactions. Provided this context, we observed that a majority of miRNA mutants that impacted DCV or FHV infection increased mortality (67%). One interpretation for this result is that miRNA loss-of-function disrupts relevant pathways, such as homeostasis or cellular environment, which negatively impacts the fly’s ability to restrict or tolerate virus infection. This supports the hypothesis that the majority of miRNAs, when functioning properly, contribute positively to the fly’s viral defense (be it directly or indirectly). Despite this, it is important to recognize that a third of miRNA mutants still resulted in a decrease in mortality (33%). These miRNAs may conversely regulate processes that the viruses rely on, such as metabolism or cellular machinery. Additionally, it is worth considering that certain miRNAs have been established to directly interact with viral RNA, modulating virus replication (16, 40, 41).

We identified that over half of the miRNA mutants that impacted either DCV or FHV impacted both (58%). One potential explanation for this overlap is that a proportion of miRNAs are involved in pathways or processes relevant to general virus infection or defense, such as immune response or cellular environment (33). Alternatively, this overlap could be a consequence of random chance. The remaining proportion, which did not overlap, indicates that many miRNAs impact pathways and processes that appear to be virus-specific. This is consistent with our understanding of diverse virus life cycles, which vary substantially in the host cells viruses target, how they enter those cells, and which cellular pathways they exploit (4, 33, 36). We identified that the majority of miRNA mutants that had shared impact on DCV and FHV shared the same directionality of impact (80%). This could be interpreted as evidence supporting generalized pathways or interactions. However, this result could also be a consequence of our observation that the majority of miRNA mutants increased mortality. Taken together, these results suggest that the functional impact of miRNAs on viruses is complex and highly diverse, made up of many types of interactions.

Accumulation of virus is commonly linked to virus-induced mortality; however, this is not always the case (33, 42). To follow up on the initial functional impact screen, we determined the impact of miRNA dysregulation on DCV and FHV RNA levels for six miRNA mutants of interest. We identified that some of these mutants had an impact on DCV and FHV viral RNA accumulation. For others, we found no evidence of a change in viral RNA levels using this experimental design, noting that accumulation dynamics are variable, and impacts could be divergent at alternate time points (42). Viral RNA accumulation results provide interesting insights into the potential mechanisms underpinning observed impacts on mortality (43). Changes in viral RNA may suggest the miRNA impacts virus replication dynamics, restricting or promoting virus replication (16, 18, 19). Conversely, no change in viral RNA can imply an impact on the fly’s tolerance to infection, withstanding or succumbing to stress more easily. Notably, miR-31b and miR-304 mutants were both identified to reduce viral RNA accumulation in all three viruses at 3 dpi, as well as reduce DCV- and FHV-induced mortality. This provides evidence to suggest that certain miRNAs can confer a generalized effect that impacts distinct RNA viruses similarly. Interestingly, the miR-263b mutant exhibited a reduction in FHV RNA, despite having an increased mortality rate when infected with the virus. This may demonstrate the variability of accumulation through time or alternatively point towards miRNAs having multiple conflicting impacts—both changing virus accumulation dynamics and tolerance to infection.

In this study, we identified the functional impacts of miRNAs on viral RNA accumulation and the outcome of infection. To understand the mechanisms that may underpin these observed impacts, insight into individual miRNA-gene-virus interactions is important. For example, miR-31b and miR-283 have been shown to modulate Wnt and Hedgehog signaling, respectively (44, 45), both highly conserved pathways with some implication in virus infection (46, 47). Interestingly, while miR-31b demonstrated impacts on DCV, FHV, and SINV, miR-31a impacted no viruses. While miR-31a and miR-31b share complete seed region overlap, they have otherwise divergent sequences and expression patterns, which likely explain the observed disparity (13, 31). Several of the miRNAs identified as impacting viruses in this study have known links to the insect immune system. miR-137, which impacted DCV and FHV survival, has been identified to modulate both *Myc* and *Ptp61F*, known regulators of the Immune deficiency and JAK/STAT pathways, respectively (48, 49). miR-274 is involved in the modulation of JAK/STAT and MAPK pathways, both implicated in immunity (50–52). miR-304 has been demonstrated to modulate muscle function (53)—muscle cells being sites of infection for many viruses, including DCV (54). miR-276b had no clear ties to immunity but has been identified to impact circadian rhythm, sleep, and dendrite development (55, 56). Overall, the available mechanistic literature provides useful insight to contextualize some of the observed miRNA impacts, with some clearly implicated in immunity.

The evolutionary conservation of miRNAs is complex and variable (31, 57, 58). The conservation of individual miRNAs reflects evolutionary pressure and fitness benefits for the organism (59, 60). Therefore, we investigated whether miRNAs may be subject to sequence conservation based on their impact on immunity, or more specifically, impact on virus. We attempted multiple parallel analyses (detailed in Output S2) but identified no evidence of a relationship between impact on virus and sequence conservation. Given how heavily viruses rely on the cellular environment for proliferation (15, 34, 36), this result may support the hypothesis that the impacts of miRNAs in virus infection are largely contributed to by disruptions in regular cell function. However, if the contributions of miRNA to virus infection are as diverse and variable as our screen suggests, it may greatly complicate the identification of such relationships between impact and conservation. One consideration which may contribute to the above results is the idea of evolutionary turnover, where certain conserved miRNAs do not maintain the same targets, expression profiles, or impacts between organisms (57, 61). Branching from this, it is unclear how natural infection with each of these viruses impacts evolutionary fitness (62–64). Additionally, it is important to identify that miRNA impact data for three viruses may not be robust enough to identify a clear correlation between conservation status and miRNA impact.

We used a miRNA loss-of-function mutant panel to investigate the extent to which miRNAs functionally impact virus infection *in vivo*. The results identify that most miRNA mutants impacted at least one of the three viruses, and that almost half of them impacted multiple viruses. Taken together, this study has allowed us to show that the dysregulation of miRNAs has broad and varying impacts on virus infection.

## MATERIALS AND METHODS

### Cell culture and fly husbandry

The miRNA^KO^ panel was obtained from the Bloomington Drosophila Stock Centre (Table 1), and the *w*^1118^ control line was an existing stock within the Johnson Lab. The panel was randomly selected from those that were available. Flies were maintained at 25 ± 0.5°C on standard dextrose-cornmeal media, with a 12-h light-dark cycle (65, 66). *Wolbachia*-free lines were generated using tetracycline treatment as described in reference 67. Virus-free lines were generated by dechorionation of embryos as previously described (23, 68). The absence of *Wolbachia* and contaminating virus was confirmed using previously described polymerase chain reaction (PCR) primers (67). Fly lines were also screened for *Pastrel* haplotype using PCR primers specific to the 2469 A/G loci as identified in reference 69 (methods provided in Output S1). All flies utilized were of the susceptible *Pst* haplotype. The generation of mutant fly lines can have unintended effects on fly fitness, making it vital to account for (70). To address this, we utilized the general fitness data provided in reference 37 and analyzed the impact of mock-injection on fly survival (Fig. S1 and S2; Tables S6 and S7). None of the mock-injected mutants had differential survival compared to control flies throughout the period of experimentation.

**TABLE 1:** *D. melanogaster* stocks used in this study

BDSC stock no.	Background	miRNA affected
58915	w*	miR-2b-1
58967	w*	miR-9c
58880	w*	miR-10
58890	w*	miR-11
58928	w*	miR-31a
58929	w*	miR-31b
58934	w*	miR-87
58937	w*	miR-92a
58892	w*	miR-133
58893	w*	miR-137
58900	w*	miR-219
58903	w*	miR-263b
58904	w*	miR-274
58907	w*	miR-276b
58909	w*	miR-278
58911	w*	miR-282
58912	w*	miR-283
58914	w*	miR-285
58918	w*	miR-304
58931	w*	miR-375
58939	w*	miR-932
58942	w*	miR-957
58943	w*	miR-958
58946	w*	miR-965
58947	w*	miR-966
58948	w*	miR-967
58952	w*	miR-971
58959	w*	miR-986
58961	w*	miR-988
58962	w*	miR-989
58963	w*	miR-990
58966	w*	miR-999
58885	w*	miR-1007
58888	w*	miR-1014

### Virus preparation

Stocks of DCV (isolate EB) and FHV were prepared by sucrose-gradient purification as described previously (71, 72). Stock titers were determined by the Reed-Muench method of 50% Tissue Culture Infectious Dose using *Drosophila* S2 cell culture, measured as IU/mL (73). DCV and FHV stocks were stored at −20°C.

Stocks of SINV (isolate AR339) were prepared by inoculating C6/36 monolayers with virus at a multiplicity of infection of 0.1. After a 2-h incubation at 28°C, the SINV inoculum was replaced with fresh growth media, supplemented with 2% fetal bovine serum (FBS). The infected cells were then left to grow at 28°C for 5 days, before being collected and clarified through centrifugation (1,500 × *g* at 4°C for 10 min). The clarified supernatant was then passed through a 0.45 µM filter to remove any cellular debris. Additional FBS was added to the filtered supernatant and stored at −80°C. SINV stock titers were determined by enzyme-linked immunosorbent assay.

Virus stocks were screened for contaminating virus using previously described PCR primers (66).

### Survival bioassay with DCV and FHV

Four to seven-day-old female *D. melanogaster* flies were challenged with either phosphate-buffered saline (PBS, injection control) or virus suspension (diluted to working concentration in PBS). The flies were individually challenged through microinjection with pulled borosilicate glass needles using the Nanoject II microinjector (Drummond Scientific). Flies were anesthetized with CO_2_, and inoculated with 50.6 nL of either PBS, DCV (10^7^ IU/mL), or FHV (10^8^ IU/mL) into the upper lateral part of the abdomen. Vials of 15 flies were used per technical replicate, while a total of three vials, one of PBS and two of virus, represented each independent biological replicate. When balancer chromosomes were present, only homozygous individuals were challenged. Flies were transferred onto fresh media every 3 days, with mortality scored daily until all virus-infected flies had died. Mortality within the first 24 h post-injection was considered related to needle-stick injury and censored. At least three independent biological replicates were performed for each tested line.

### Accumulation assay

Viral RNA was quantified by accumulation assay. Flies were challenged with DCV/FHV (as above), SINV suspension (10^5^ IU/mL), or PBS injection control using the same layout described for the above survival bioassay. The flies from each vial were collected at 3 dpi and stored at −20°C. Five flies per vial were pooled together and homogenized in 1 mL TRIzol Reagent (Invitrogen) using two glass beads and the TissueLyser II (Qiagen) for 1 min 30 s at 30 Hz. The samples were clarified by centrifugation at 21,000 × *g* for 10 min at 4°C, and RNA was extracted from the supernatant as per the manufacturer’s instructions. Extracted RNA was quantified using an Epoch spectrophotometer (Biotek). For each sample, 1 µg of total RNA was taken and treated with DNase (Promega) as per the manufacturer’s instructions. Complementary DNA (cDNA) was synthesized using SuperScript III Reverse Transcriptase (Invitrogen) and a Virus/*RpL32* reverse primer mix. Finally, qPCR was performed using Platinum SYBR Green qPCR SuperMix-UDG (Invitrogen) and the Rotor-Gene 6000 Thermocycler (Qiagen). Thermocycling profile was as follows: 50°C 2 min, 95°C 2 min, 40 cycles of (95°C 10 s, 58°C 20 s, and 72°C 20 s). Melt curve analysis was conducted for quality control. Virus primers targeted the SINV subgenomic E2 glycoprotein, DCV replicase polyprotein, and Flock House virus hypothetical protein gp1, and are provided in Table 2. Three to six independent replicates were performed for each tested line.

**TABLE 2:** Oligonucleotides used in this study

Name	Sequence (5′ → 3′)	Amplicon	Purpose	Target	RefSeq	Reference
SINV forward	CACAGTGTACGACCGTCTGAA	178	qPCR	JX682537.1	OK539682.1	
SINV reverse	AACGGTTCCGGTCTTGTAGTC					
DCV forward	AGCCCAGAATTACAACGCCA	203	qPCR	NP_044945.1	NC_001834.1	(40)
DCV reverse	CTTCAACGCCAATACCGCTG					
FHV forward	GGTCCGATCAGGTGTCCTCA	139	qPCR	NP_689442.1	NC_004144.1	(40)
FHV reverse	TGCAACCGGGAATTGCACAG					
*Rpl32* forward	GACGCTTCAAGGGACAGTATCTG	141	qPCR	FBgn0002626	NT_033777.3	(40)
*Rpl32* reverse	AAACGCGGTTCTGCATGAG					

### Statistical analyses

All analyses were conducted in R v4.5.1 (74), with data and scripts deposited in GitHub (https://github.com/tysongthomson/Thomson-JVI-2026). References for all R packages can be found in Table S8.

### Survival bioassays

Differences in survival between miRNA mutant and control were analyzed using restricted mean survival time (RMST) for DCV and FHV (75, 76). Given that a major subset of the data violated the proportional hazards assumption, RMST was identified as an informative alternative to more typical survival analyses (40, 77, 78). RMST analysis was achieved in R using the *survRM2* package and *rmst2* function (79). Truncation time was identified as the higher of the maximum survival times between mutant and control, and biological replicate was included as a covariate. This ensured RMST estimated the approximate mean survival time difference of mutant vs control.

### RNA accumulation

Differences in viral RNA accumulation between mutant and control were analyzed using mean normalized expression. Viral RNA was normalized to the reference gene *RpL32* using the Q-Gene formula in R (80). Expression data were log_2_ transformed for analysis. The transformed mean normalized expression values for each miRNA mutant line were compared to control using a paired two-tailed *t*-test as established in reference 23. Additionally, 95% confidence intervals were estimated for plotting using bootstrapping. Significance values for the SINV analysis were adjusted using a multiple comparisons correction (81, 82).

### Conservation analysis

Screened miRNA sequences were extracted from miRBase (31) using R, yielding 68 mature miRNA sequences. These sequences were compared to the sequences for three well-studied *Dipterans* to establish conservation level: *Drosophila simulans* (another species within the observed DCV host range [83]), *Aedes aegypti*, and *Anopheles gambiae* (medically significant mosquito species). Conservation analyses were performed to address two questions. (i) Are miRNAs that impact virus infection more likely to be conserved? (ii) Are conserved miRNAs more likely to target more/multiple viruses?

Detailed methods, outputs, and interpretation are provided in Output S2.

## Data Availability

Data and R scripts for all analyses have been deposited in GitHub (https://github.com/tysongthomson/Thomson-JVI-2026).

## SUPPLEMENTAL MATERIAL

Output S1 jvi.00850-26-s0001.docx Methods for Pst screen.

Output S2 jvi.00850-26-s0002.docx Conservation analysis.

Fig. S1 jvi.00850-26-s0003.pdf DCV survival curves.

Fig. S2 jvi.00850-26-s0004.pdf FHV survival curves.

Tables S1-S7 jvi.00850-26-s0005.xlsx Statistical analysis results (RMST and t test).

Table S8 jvi.00850-26-s0006.docx R packages used in this study.

ASM does not own the copyrights to Supplemental Material that may be linked to, or accessed through, an article. The authors have granted ASM a non-exclusive, world-wide license to publish the Supplemental Material files. Please contact the corresponding author directly for reuse.
