# Supplementary material for: Dysregulation of miRNAs has broad impacts on virus infection in Drosophila
Source: J Virol. 2026 Jul 2;100(7):e00850-26. doi: 10.1128/jvi.00850-26 (PMC13386962; doi:10.1128/jvi.00850-26)
Supplement: Output S1 — Methods for Pst screen. [file jvi.00850-26-s0001.docx]

## **Supplementary Methods: Pst Screen**

**Background and Rationale**

*Pastrel* (*Pst*) is a gene which contributes to the susceptibility of *Drosophila melanogaster* individuals to certain virus infections, including Drosophila C virus (1). Variations in susceptibility to infection are based on haplotype, with important single nucleotide polymorphisms (SNP) at several loci within *Pst* (1). An A/G SNP at *Pst* location 2469 has been identified as the most significant contributor to virus susceptible and resistant individuals. Therefore, we utilised the following assay to screen this locus.

**Methods**

To screen for the impact of *Pst* on survival differences observed for our miRNA mutants, we screened the 2469 A/G loci using two primer pairs (Table 1). Genomic DNA was extracted from each fly line using the DNeasy Blood & Tissue Kit (Qiagen) according to manufacturer’s instruction, with modifications to follow. Five female *D. melanogaster* individuals were collected and suspended in 200 µL Phosphate-Buffered Saline. The solution was homogenised using 2 glass beads and a TissueLyser II (Qiagen) for 1 min 30 s at 30 Hz. 20 µL Proteinase K was added to the solution and protocol proceeded according to manufacturer’s procedure. With genomic DNA as the template, PCR was performed using ½ Reactions of the MyTaq DNA Polymerase Kit (Bioline), using 0.25 µL MyTaq DNA polymerase and manufacturer’s instruction. To assist in efficient amplification, the PCR reactions were supplemented to a final MgCl_2_ concentration of 25mM. PCR Products were run on a 2% Agarose Gel and visualised. The primers were designed as such The *Pst*-2469-A primers would produce a product if the A SNP was present, while the *Pst*-2469-G primers would produce a product if the G SNP was present.

**Table 1: *Pst* Primer Pairs.**

| **Name** | **F/R** | **Sequence (5' → 3')** |
| --- | --- | --- |
| *Pst*-2469-A | F | GCATGGTGTCCATGAAGAC |
| *Pst*-2469-A | R | TCCTCGACAGGAACCCAGTA |
| *Pst*-2469-G | F | GCATGGTGTCCATGAAGAT |
| *Pst*-2469-G | R | CCGATGGCAAAGGATTTTT |

**References**

1. Magwire MM, Fabian DK, Schweyen H, Cao C, Longdon B, Bayer F, Jiggins FM. 2012. Genome-wide association studies reveal a simple genetic basis of resistance to naturally coevolving viruses in *Drosophila melanogaster*. PLoS Genet 8:e1003057.
