# Supplementary material for: Dysregulation of miRNAs has broad impacts on virus infection in Drosophila
Source: J Virol. 2026 Jul 2;100(7):e00850-26. doi: 10.1128/jvi.00850-26 (PMC13386962; doi:10.1128/jvi.00850-26)
Supplement: Output S2 — Conservation analysis. [file jvi.00850-26-s0002.docx]

**Output S2. Conservation Analysis.**

Data and scripts for this analysis are deposited on GitHub (<https://github.com/tysongthomson/Thomson-JVI-2026>).

**Identification of Conserved miRNAs.** To be identified as conserved, the matching miRNA required a 100% seed region match and ≥80% nucleotide similarity. For each miRNA gene, the most conserved mature miRNA arm was selected for each (e.g. when a miRNA -3p strand was not conserved, and a -5p strand was conserved, the miRNA was considered conserved). All of the mature *D. melanogaster* miRNAs from screened genes were matched to three species: *Drosophila simulans (Dsi), Aedes aeygpti (Aae)* and *Anopheles gambiae (Aga)*.

**We identified the following conservation levels:**

*D. simulans*: 26/34 or 76% of miRNAs were considered conserved.

*A. aegypti*: 25/34 or 74% of miRNAs were considered conserved.

*A. gambiae*: 20/34 or 59% of miRNAs were considered conserved.

**Rationale.** The dynamics of miRNA conservation is complex (2, 3), and our study provides a unique opportunity to query the potential correlation between miRNA impact on virus and sequence conservation. There is limited information available surrounding this sort of analysis, and as such, there are multiple ways of approaching it. Therefore, in an attempt to be as robust as possible, we performed a series of analyses as reported below.

**We sought to address two main questions:**

1. Are miRNAs which impact virus infection more likely to be conserved (than no-impact)?
2. Are conserved miRNAs more likely to target multiple viruses (than non-conserved)?

**Considerations.** It is important to identify that a sample size of 34 miRNAs over 3 viruses may not be large enough for certain analyses. Additionally, given the fact that each miRNA mutant results in the dysregulation of both -3p and -5p strands, it is not possible to identify which strand is contributing to viral impact. Therefore, our analyses combined -3p and -5p strands for both impact and conservation. Finally, this analysis assumes all miRNAs could have the capacity to impact immunity.

**Approach:**

To address Question 1:

1. Fisher's exact test: Comparing Conservation and Impact on virus (binary).
2. Linear Model: Comparing Conservation and Impact on virus (continuous).
3. Wilcoxon rank-sum test: Comparing Conservation and Impact on virus (continuous).
4. Fisher's exact test: Comparing Conservation and Impact on virus (categorical).
5. Linear Model: Comparing Conservation and Impact on virus (continuous, absolute).

To address Question 2:

1. Ordinal Logistic Model: Comparing Conservation and Number of Viruses impacted (categorical).
2. Wilcoxon rank-sum test: Comparing Conservation and Number of Viruses impacted (categorical).

**1a: Fisher's exact test: Comparing Conservation and Impact on virus (binary).**

**Data Structure**

Conservation: TRUE/FALSE binary for each of Dsi, Aae and Aga

Virus Impact: TRUE/FALSE binary for each of DCV, FHV and SINV

**Null Hypothesis:** There is no correlation between whether miRNA impacts virus and conservation status.

| test | p_value |
| --- | --- |
| DCV_dsi | 1 |
| DCV_aae | 0.43 |
| DCV_aga | 1 |
| FHV_dsi | 1 |
| FHV_aae | 0.71 |
| FHV_aga | 1 |
| SINV_dsi | 1 |
| SINV_aae | 0.39 |
| SINV_aga | 1 |

**Interpretation**: P-value is above 0.05, cannot refute Null hypothesis. Therefore, there is no evidence that conservation level and miRNA impact on virus is correlated. This was consistent for all virus and species combinations.

**1b: Linear Model: Comparing Conservation and Impact on virus (continuous).**

**Data Structure**

Conservation: TRUE/FALSE binary for each of Dsi, Aae and Aga

Virus Impact: Continuous RMST diff/Log_2_FC for each of DCV, FHV and SINV

**Null Hypothesis:** There is no correlation between conservation status and magnitude of virus impact.

**Note:** This analysis does not distinguish between both positive/negative Virus impacts.

| test | estimate | p_value |
| --- | --- | --- |
| DCV_dsi | 0.34 | 0.60 |
| DCV_aae | 0.93 | 0.13 |
| DCV_aga | 0.39 | 0.50 |
| FHV_dsi | 0.30 | 0.44 |
| FHV_aae | 0.35 | 0.36 |
| FHV_aga | 0.02 | 0.97 |
| SINV_dsi | -0.96 | 0.32 |
| SINV_aae | -1.00 | 0.28 |
| SINV_aga | -0.05 | 0.95 |

Estimate >0: conserved miRNAs have higher impact on virus

Estimate <0: conserved miRNAs have lower impact on virus

**Interpretation**: P-value is above 0.05, cannot refute Null hypothesis. Therefore, there is no evidence that conservation and magnitude of miRNA impact on virus is correlated. This was consistent for all virus and species combinations.

**1c: Wilcoxon rank-sum test: Comparing Conservation and Impact on virus distribution (continuous).**

**Data Structure**

Conservation: TRUE/FALSE binary for each of Dsi, Aae and Aga

Virus Impact: Continuous RMST diff/Log_2_FC for each of DCV, FHV and SINV

**Null Hypothesis:** Conservation status does not impact distribution of virus impacts.

| test | p_value |
| --- | --- |
| DCV_dsi | 0.97 |
| DCV_aae | 0.25 |
| DCV_aga | 0.60 |
| FHV_dsi | 0.46 |
| FHV_aae | 0.46 |
| FHV_aga | 0.73 |
| SINV_dsi | 0.43 |
| SINV_aae | 0.47 |
| SINV_aga | 0.99 |

**Interpretation**: P-value is above 0.05, cannot refute Null hypothesis. Therefore, there is no evidence that conservation status changes distribution of virus impacts. This was consistent for all virus and species combinations.

**1d: Fisher's exact test: Comparing Conservation and Impact on virus (categorical).**

**Data Structure**

Conservation: TRUE/FALSE binary for each of Dsi, Aae and Aga

Virus Impact: POS/NEG/FALSE categorical impact for each of DCV, FHV and SINV

**Null Hypothesis:** There is no correlation between whether miRNA impacts virus and conservation status.

| test | p_value |
| --- | --- |
| DCV_dsi | 1 |
| DCV_aae | 0.49 |
| DCV_aga | 0.82 |
| FHV_dsi | 1 |
| FHV_aae | 0.88 |
| FHV_aga | 0.81 |
| SINV_dsi | 0.80 |
| SINV_aae | 0.28 |
| SINV_aga | 1 |

**Interpretation**: P-value is above 0.05, cannot refute Null hypothesis. Therefore, there is no evidence that conservation status has an effect on whether miRNA impacts virus. This was consistent for all virus and species combinations.

**1e: Linear Model: Comparing Conservation and Impact on virus (continuous, absolute).**

**Data Structure**

Conservation: TRUE/FALSE binary for each of Dsi, Aae and Aga

Virus Impact: Continuous RMST diff/Log2FC for each of DCV, FHV and SINV (absolute value)

**Null Hypothesis:** Conservation status has no impact on magnitude of virus impact.

**Note:** This analysis utilised absolute impact of miRNA on virus, not distinguishing between pos/neg.

| test | estimate | p_value |
| --- | --- | --- |
| DCV_dsi | 0.45 | 0.35 |
| DCV_aae | 0.07 | 0.88 |
| DCV_aga | 0.12 | 0.79 |
| FHV_dsi | 0.17 | 0.54 |
| FHV_aae | 0.13 | 0.64 |
| FHV_aga | 0.17 | 0.50 |
| SINV_dsi | 0.33 | 0.65 |
| SINV_aae | 0.58 | 0.42 |
| SINV_aga | 0.01 | 0.99 |

Estimate >0: conserved miRNAs have higher impact on virus

Estimate <0: conserved miRNAs have lower impact on virus

**Interpretation**: P-value is above 0.05, cannot refute Null hypothesis. Therefore, there is no evidence that conservation level and magnitude of miRNA impact on virus is correlated. This was consistent for all virus and species combinations.

**2a: Ordinal Logistic Model: Comparing Conservation and Number of Viruses impacted (categorical).**

**Data Structure**

Conservation: TRUE/FALSE binary for each of Dsi, Aae and Aga

Virus Impact: Number of viruses impacted, Categorical (0, 1, 2, 3).

**Null Hypothesis:** Conservation status has no effect on number of viruses a miRNA impacts.

|  | estimate | p_value |
| --- | --- | --- |
| viruses_impacted_dsi | 0.26 | 0.71 |
| viruses_impacted_aae | 0.85 | 0.22 |
| viruses_impacted_aga | 0.13 | 0.84 |

Estimate >0: conserved miRNAs impact more viruses

Estimate <0: conserved miRNAs impact less viruses

**Interpretation**: P-value is above 0.05, cannot refute Null hypothesis. Therefore, there is no evidence that conservation status has an effect on number of viruses a miRNA impacts. This was consistent for all species tested.

**2b: Wilcoxon rank-sum test: Comparing Conservation and Number of Viruses impacted (categorical).**

**Data Structure**

Conservation: TRUE/FALSE binary for each of Dsi, Aae and Aga

Virus Impact: Number of viruses impacted, Categorical (0, 1, 2, 3).

**Null Hypothesis:** Conservation status has no effect on distribution of number of viruses impacted.

|  | p_value |
| --- | --- |
| viruses_impacted_dsi | 0.73 |
| viruses_impacted_aae | 0.23 |
| viruses_impacted_aga | 0.86 |

**Interpretation**: P-value is above 0.05, cannot refute Null hypothesis. Therefore, there is no evidence that conservation status has an effect on distribution of number of viruses impacted. This was consistent for all species tested.

**References**

1. Kozomara A, Birgaoanu M, Griffiths-Jones S. 2019. miRBase: from microRNA sequences to function. Nucleic Acids Research 47:D155–D162.

2. Zhao Y, Shen X, Tang T, Wu C-I. 2017. Weak regulation of many targets Is cumulatively powerful—An evolutionary perspective on microRNA functionality. Molecular Biology and Evolution 34:3041–3046.

3. Ha M, Pang M, Agarwal V, Chen Z. 2008. Interspecies regulation of microRNAs and their targets. Biochimica et Biophysica Acta (BBA) - Gene Regulatory Mechanisms 1779:735–742.
