# Supplementary material for: Dysregulation of miRNAs has broad impacts on virus infection in Drosophila
Source: J Virol. 2026 Jul 2;100(7):e00850-26. doi: 10.1128/jvi.00850-26 (PMC13386962; doi:10.1128/jvi.00850-26)
Supplement: Fig. S1 — DCV survival curves. [file jvi.00850-26-s0003.pdf]

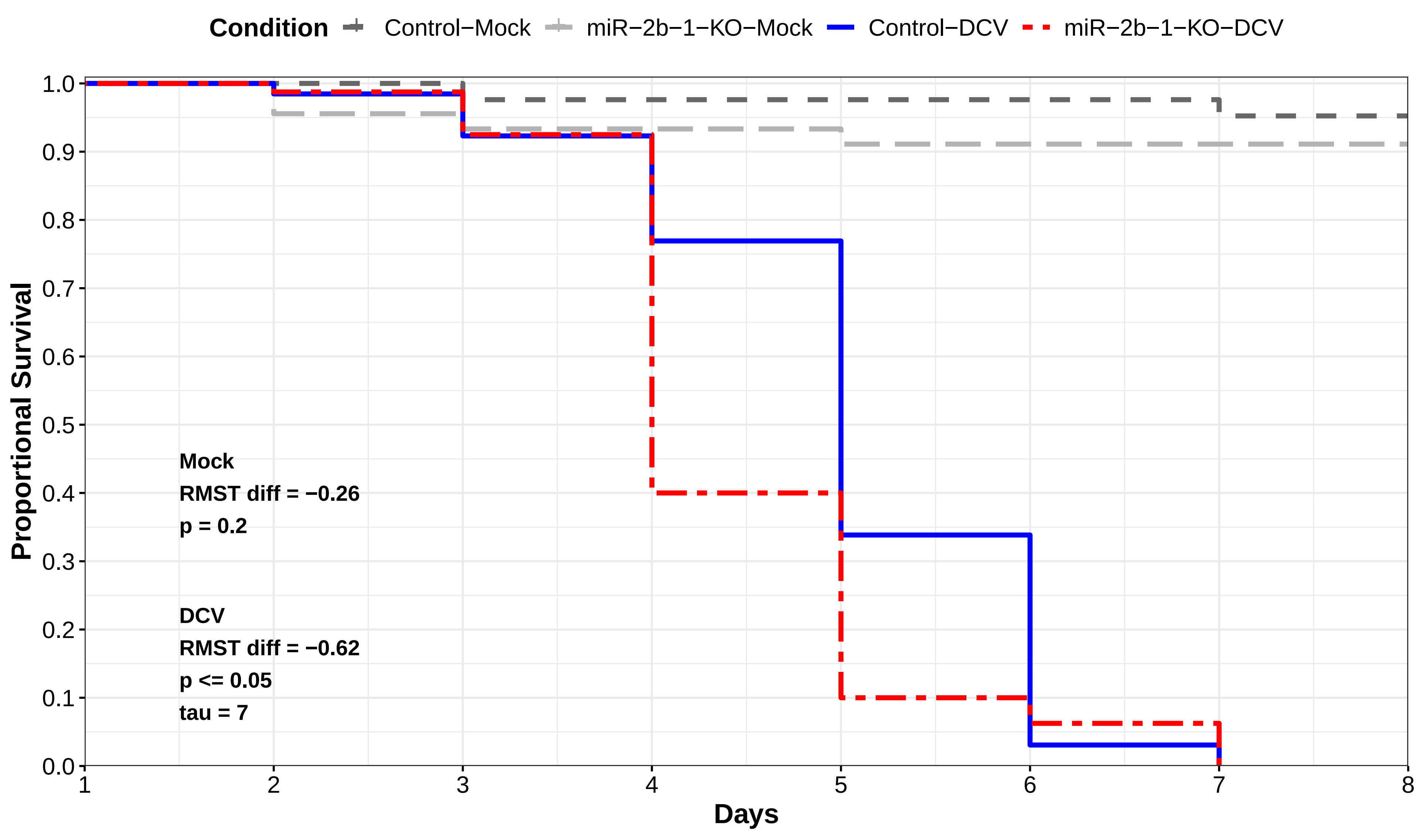

Condition
Control−Mock
miR−2b−1−KO−Mock
Control−DCV
miR−2b−1−KO−DCV
Proportional Survival
1.0
0.9
0.8
0.7
0.6
0.5
0.4
0.3
0.2
0.1
0.0
Mock
RMST diff = −0.26
p = 0.2
DCV
RMST diff = −0.62
p <= 0.05
tau = 7
1
2
3
4
5
6
7
8
Days

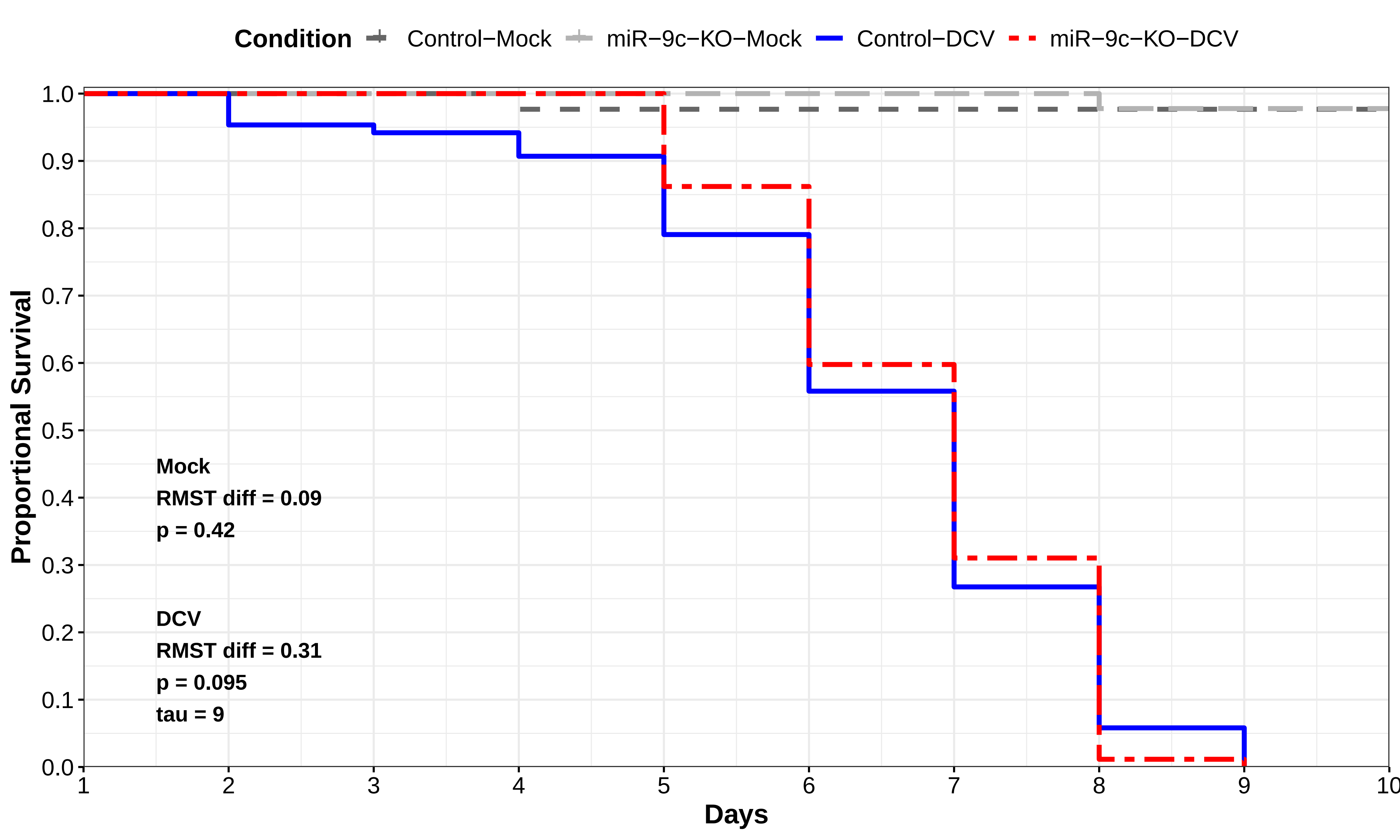

Condition
Control−Mock
miR−9c−KO−Mock
Control−DCV
miR−9c−KO−DCV
Proportional Survival
1.0
0.9
0.8
0.7
0.6
0.5
0.4
0.3
0.2
0.1
0.0
Mock
RMST diff = 0.09
p = 0.42
DCV
RMST diff = 0.31
p = 0.095
tau = 9
1
2
3
4
5
6
7
8
9
10
Days

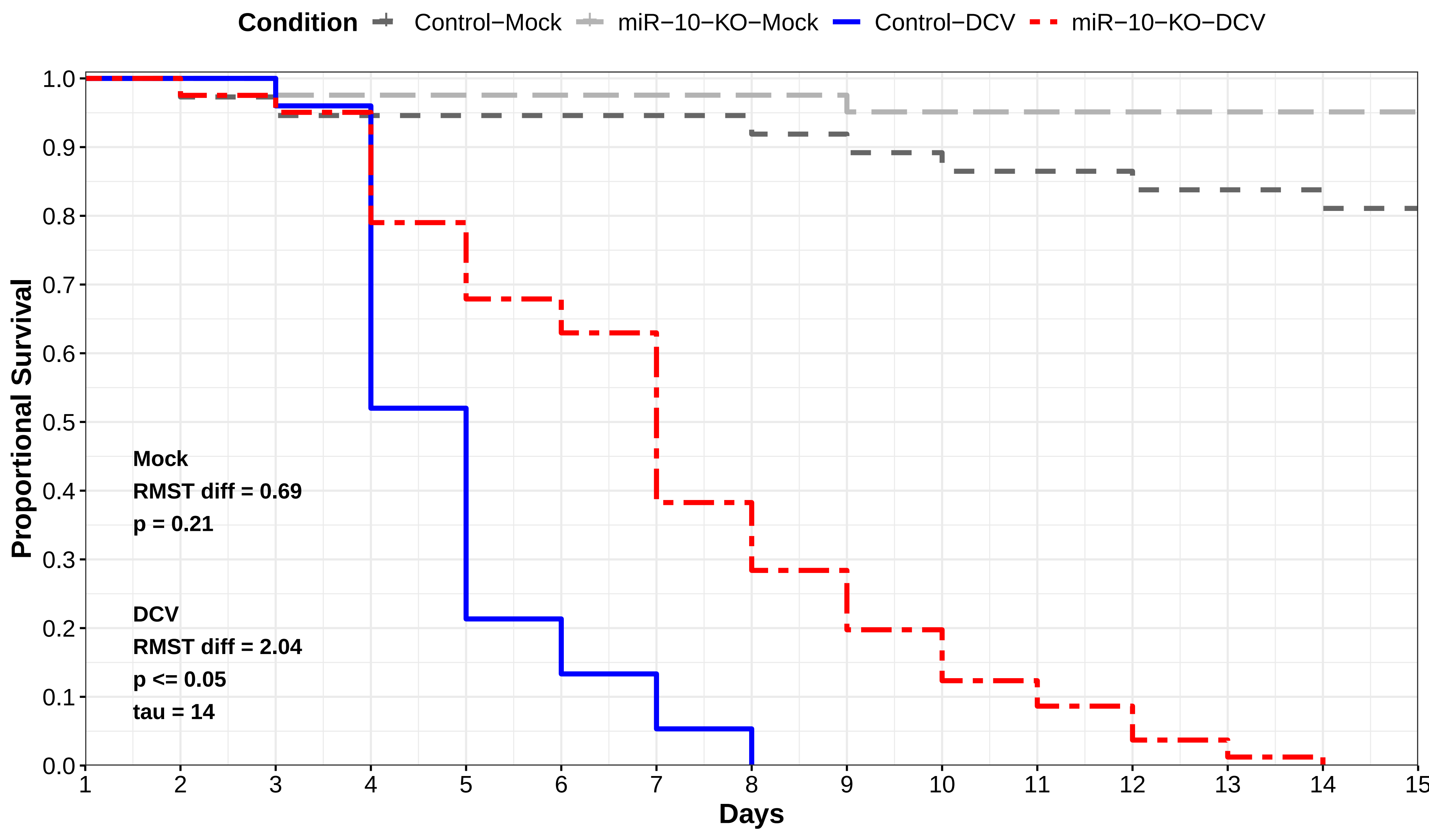

Condition
Control−Mock
miR−10−KO−Mock
Control−DCV
miR−10−KO−DCV
Proportional Survival
1.0
0.9
0.8
0.7
0.6
0.5
0.4
0.3
0.2
0.1
0.0
Mock
RMST diff = 0.69
p = 0.21
DCV
RMST diff = 2.04
p <= 0.05
tau = 14
1
2
3
4
5
6
7
8
9
10
11
12
13
14
15
Days

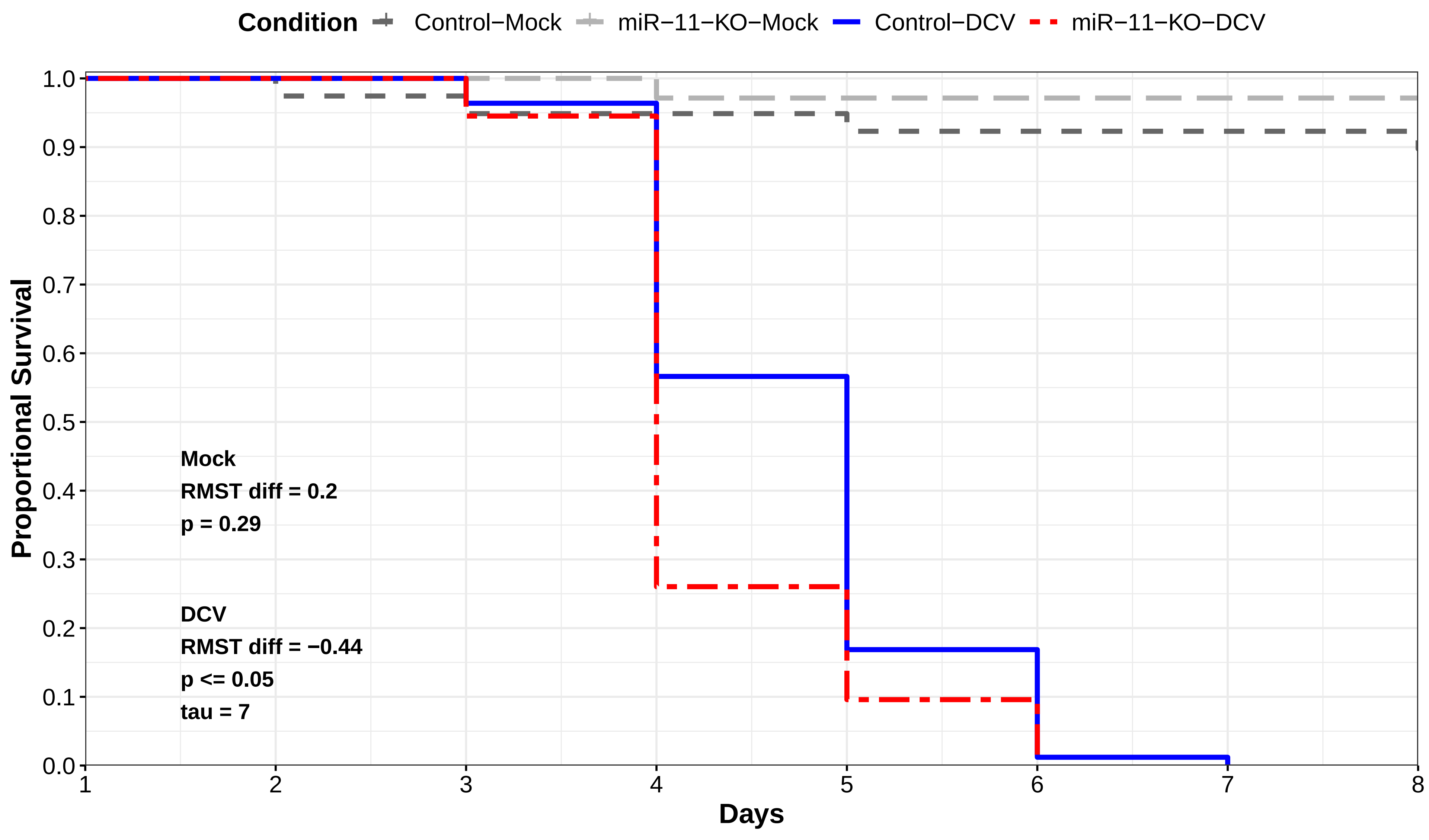

Condition
Control−Mock
miR−11−KO−Mock
Control−DCV
miR−11−KO−DCV
Proportional Survival
Days
1.0
0.9
0.8
0.7
0.6
0.5
0.4
0.3
0.2
0.1
0.0
1
2
3
4
5
6
7
8
Mock
RMST diff = 0.2
p = 0.29
DCV
RMST diff = −0.44
p <= 0.05
tau = 7

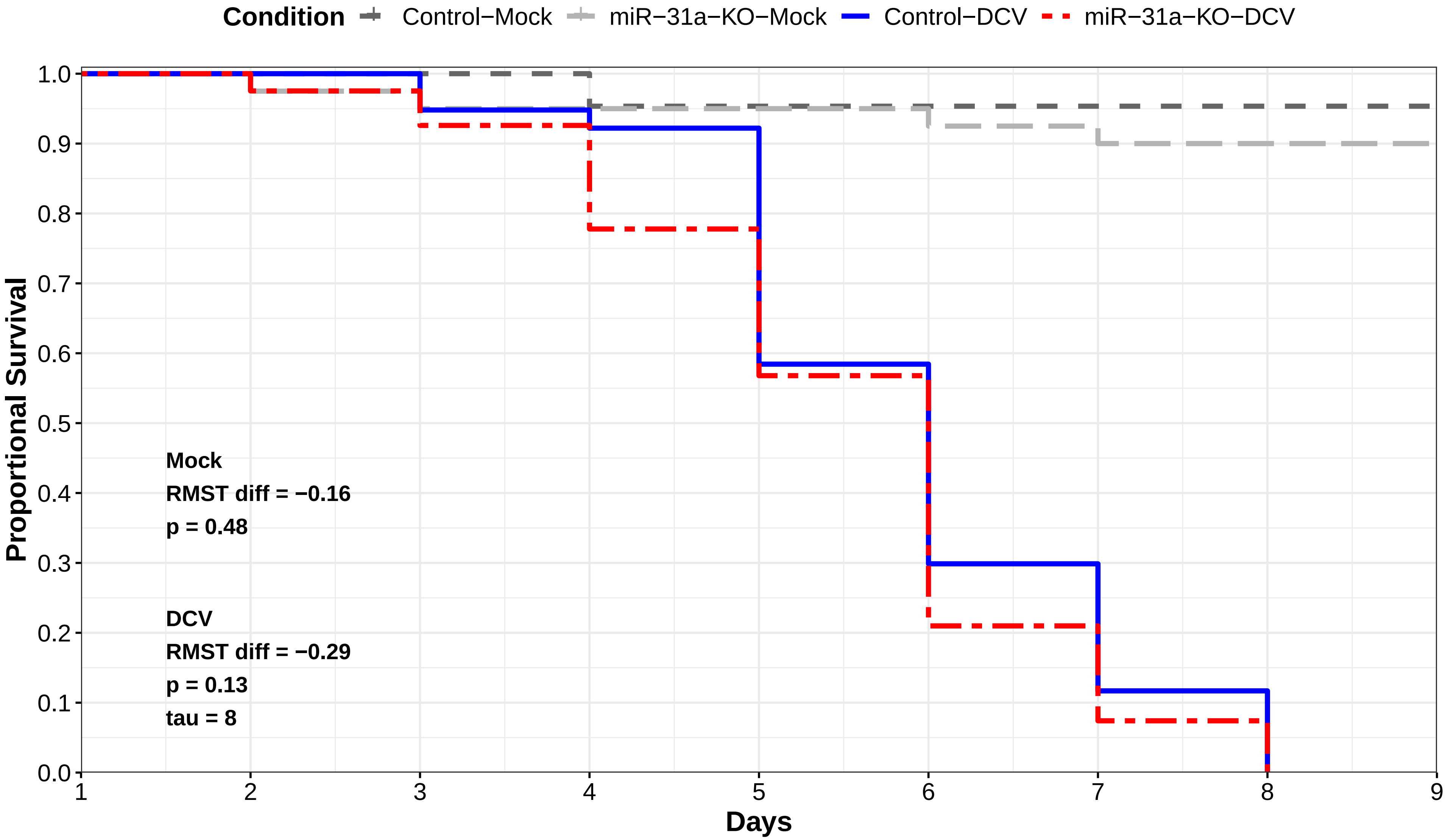

Condition
Control−Mock
miR−31a−KO−Mock
Control−DCV
miR−31a−KO−DCV
Proportional Survival
1.0
0.9
0.8
0.7
0.6
0.5
0.4
0.3
0.2
0.1
0.0
Mock
RMST diff = −0.16
p = 0.48
DCV
RMST diff = −0.29
p = 0.13
tau = 8
1
2
3
4
5
6
7
8
9
Days

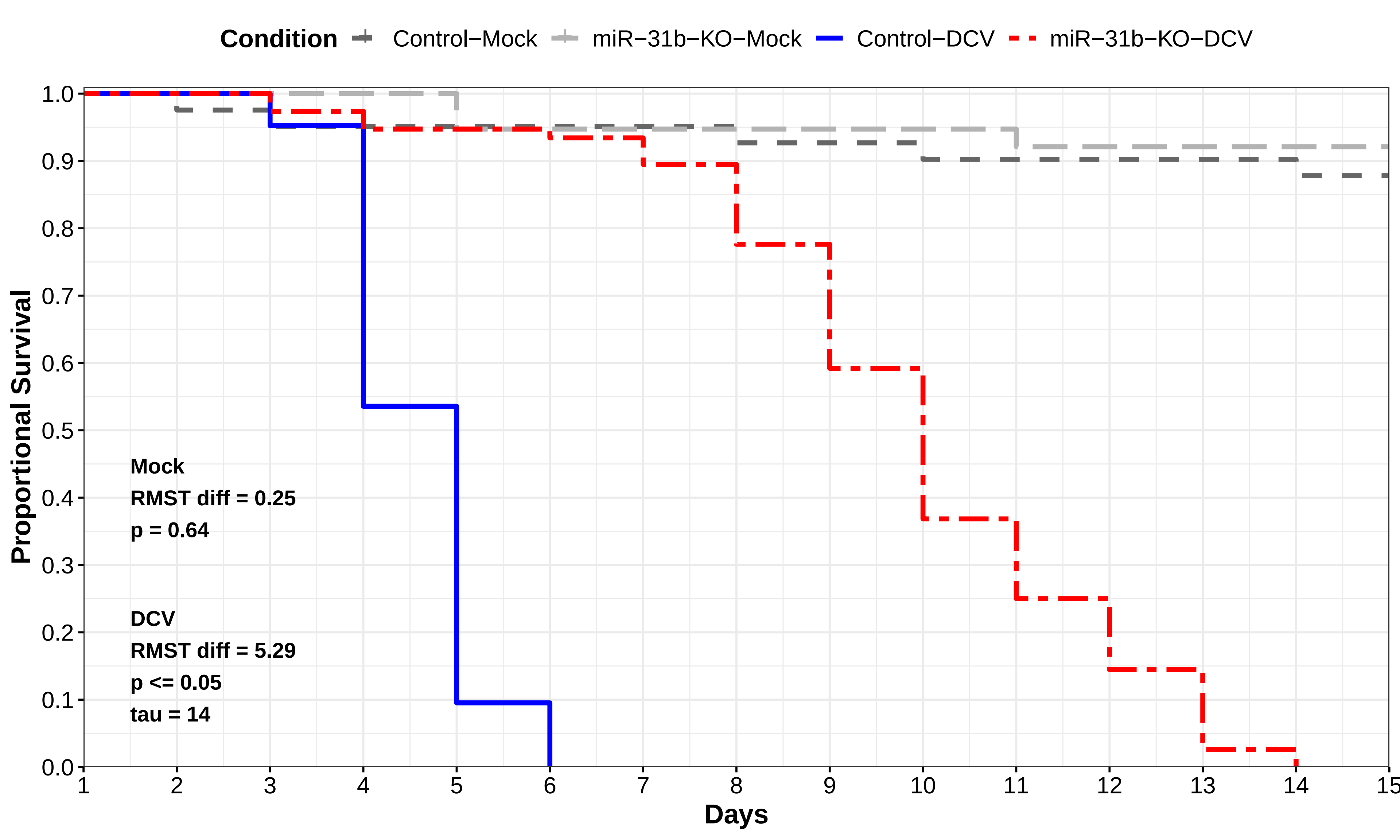

Condition
Control−Mock
miR−31b−KO−Mock
Control−DCV
miR−31b−KO−DCV
Proportional Survival
1.0
0.9
0.8
0.7
0.6
0.5
0.4
0.3
0.2
0.1
0.0
Days
1
2
3
4
5
6
7
8
9
10
11
12
13
14
15
Mock
RMST diff = 0.25
p = 0.64
DCV
RMST diff = 5.29
p <= 0.05
tau = 14

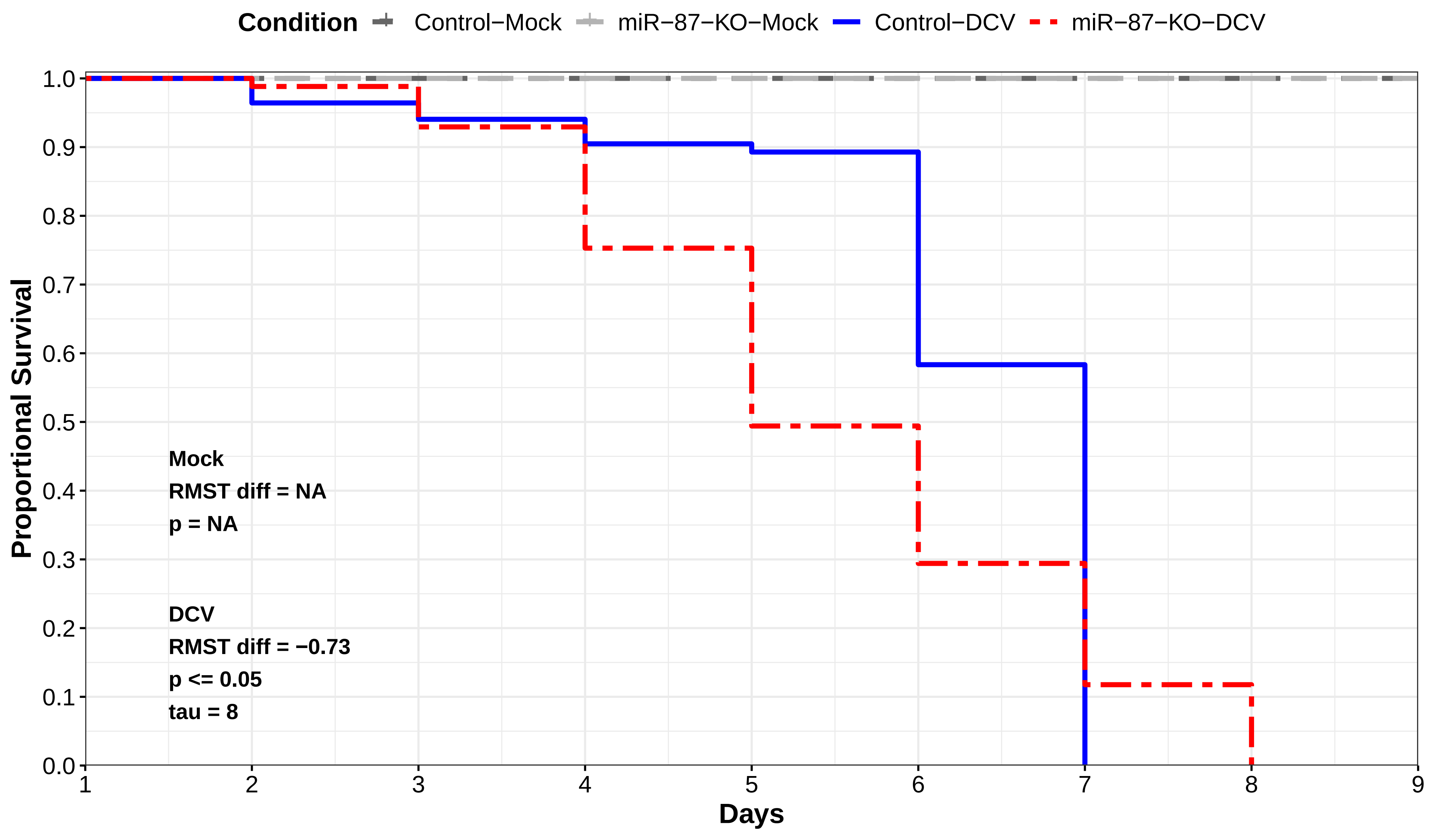

Condition
Control−Mock
miR−87−KO−Mock
Control−DCV
miR−87−KO−DCV
Proportional Survival
1.0
0.9
0.8
0.7
0.6
0.5
0.4
0.3
0.2
0.1
0.0
Mock
RMST diff = NA
p = NA
DCV
RMST diff = −0.73
p <= 0.05
tau = 8
1
2
3
4
5
6
7
8
9
Days

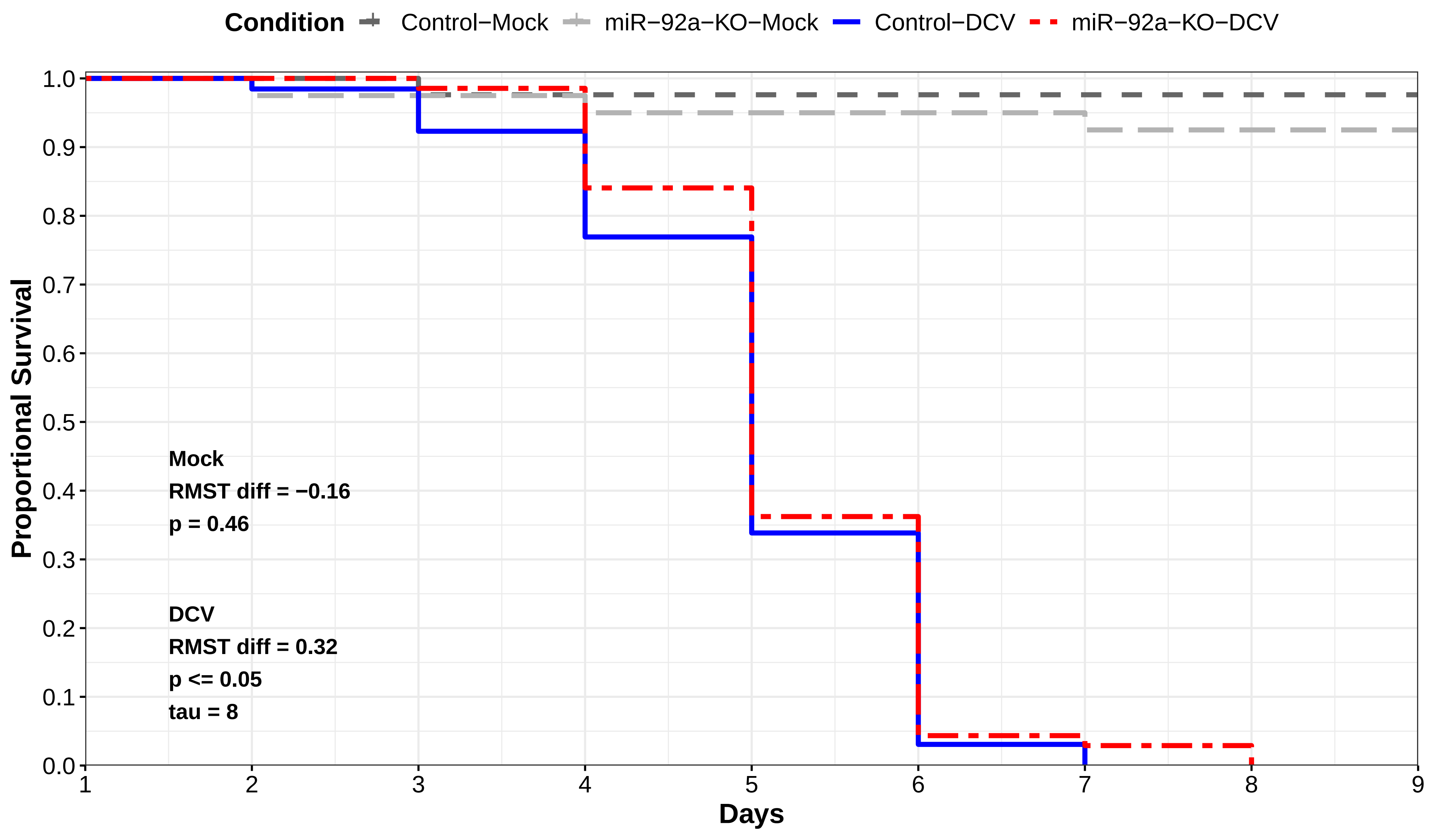

Condition
Control−Mock
miR−92a−KO−Mock
Control−DCV
miR−92a−KO−DCV
Proportional Survival
1.0
0.9
0.8
0.7
0.6
0.5
0.4
0.3
0.2
0.1
0.0
Mock
RMST diff = −0.16
p = 0.46
DCV
RMST diff = 0.32
p <= 0.05
tau = 8
1
2
3
4
5
6
7
8
9
Days

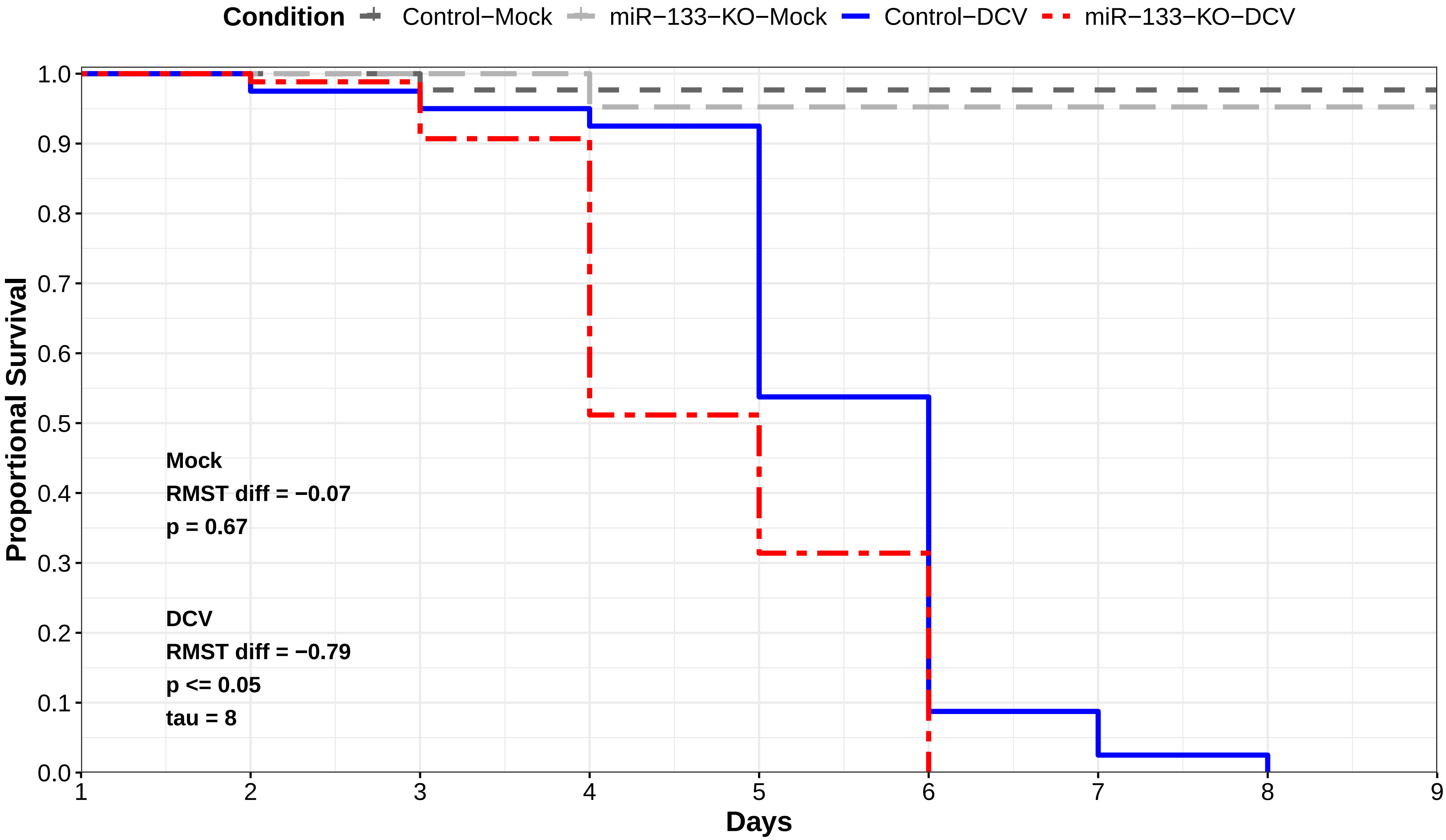

Condition
Control−Mock
miR−133−KO−Mock
Control−DCV
miR−133−KO−DCV
Proportional Survival
1.0
0.9
0.8
0.7
0.6
0.5
0.4
0.3
0.2
0.1
0.0
Mock
RMST diff = −0.07
p = 0.67
DCV
RMST diff = −0.79
p <= 0.05
tau = 8
1
2
3
4
5
6
7
8
9
Days

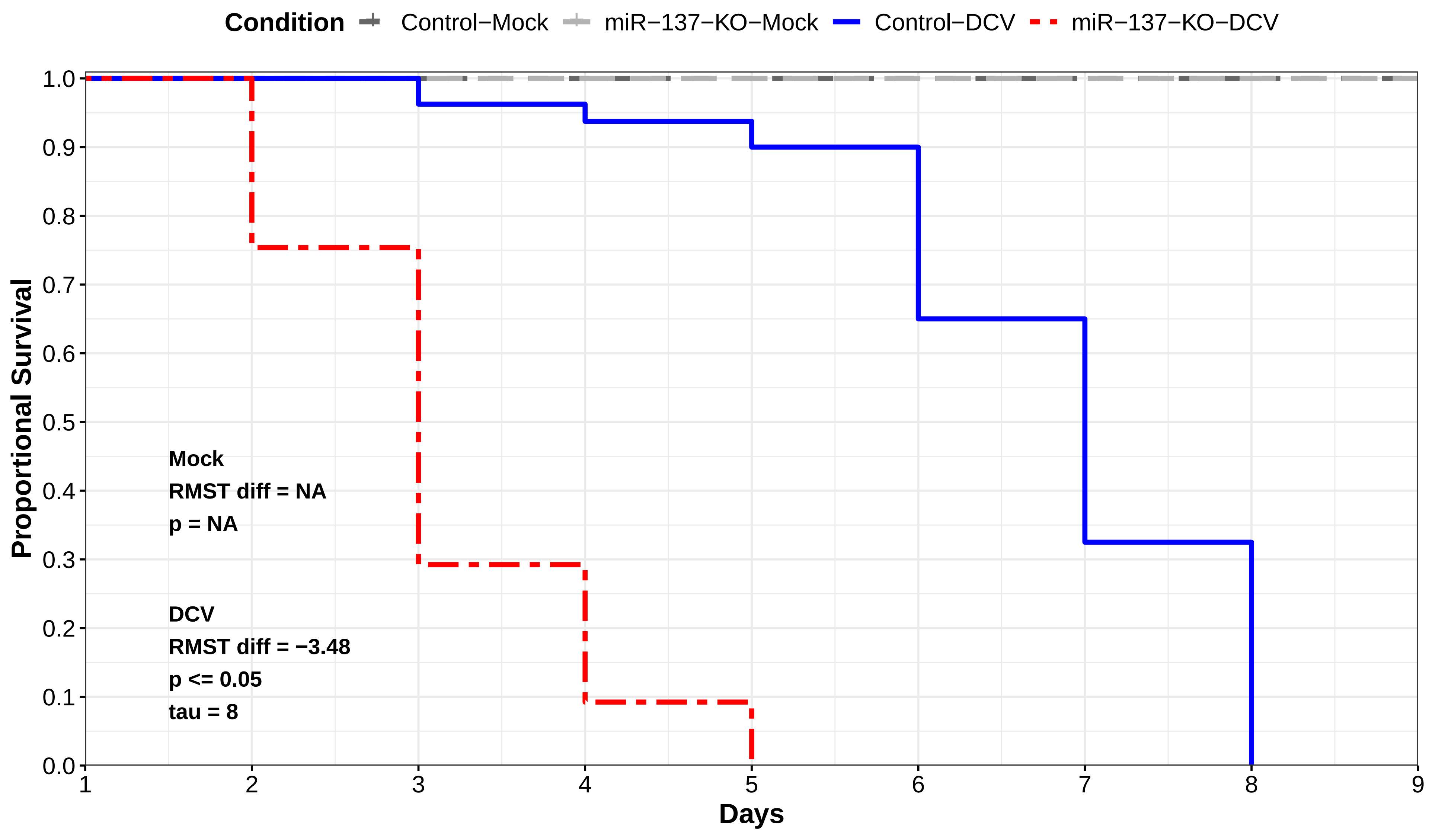

Condition
Control−Mock
miR−137−KO−Mock
Control−DCV
miR−137−KO−DCV
Proportional Survival
1.0
0.9
0.8
0.7
0.6
0.5
0.4
0.3
0.2
0.1
0.0
Mock
RMST diff = NA
p = NA
DCV
RMST diff = −3.48
p <= 0.05
tau = 8
1
2
3
4
5
6
7
8
9
Days

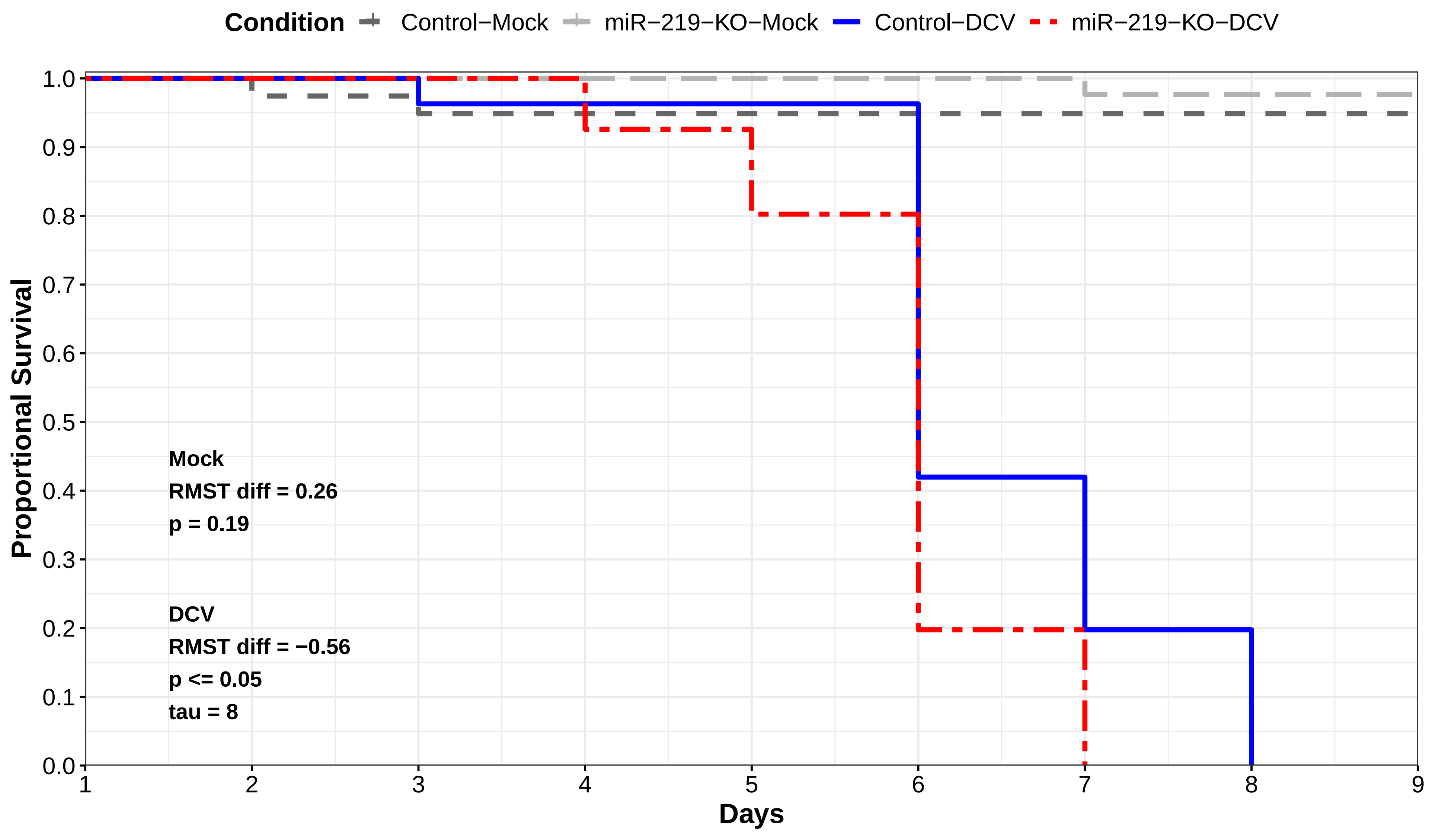

Condition
Control−Mock
miR−219−KO−Mock
Control−DCV
miR−219−KO−DCV
Proportional Survival
1.0
0.9
0.8
0.7
0.6
0.5
0.4
0.3
0.2
0.1
0.0
Mock
RMST diff = 0.26
p = 0.19
DCV
RMST diff = −0.56
p <= 0.05
tau = 8
1
2
3
4
5
6
7
8
9
Days

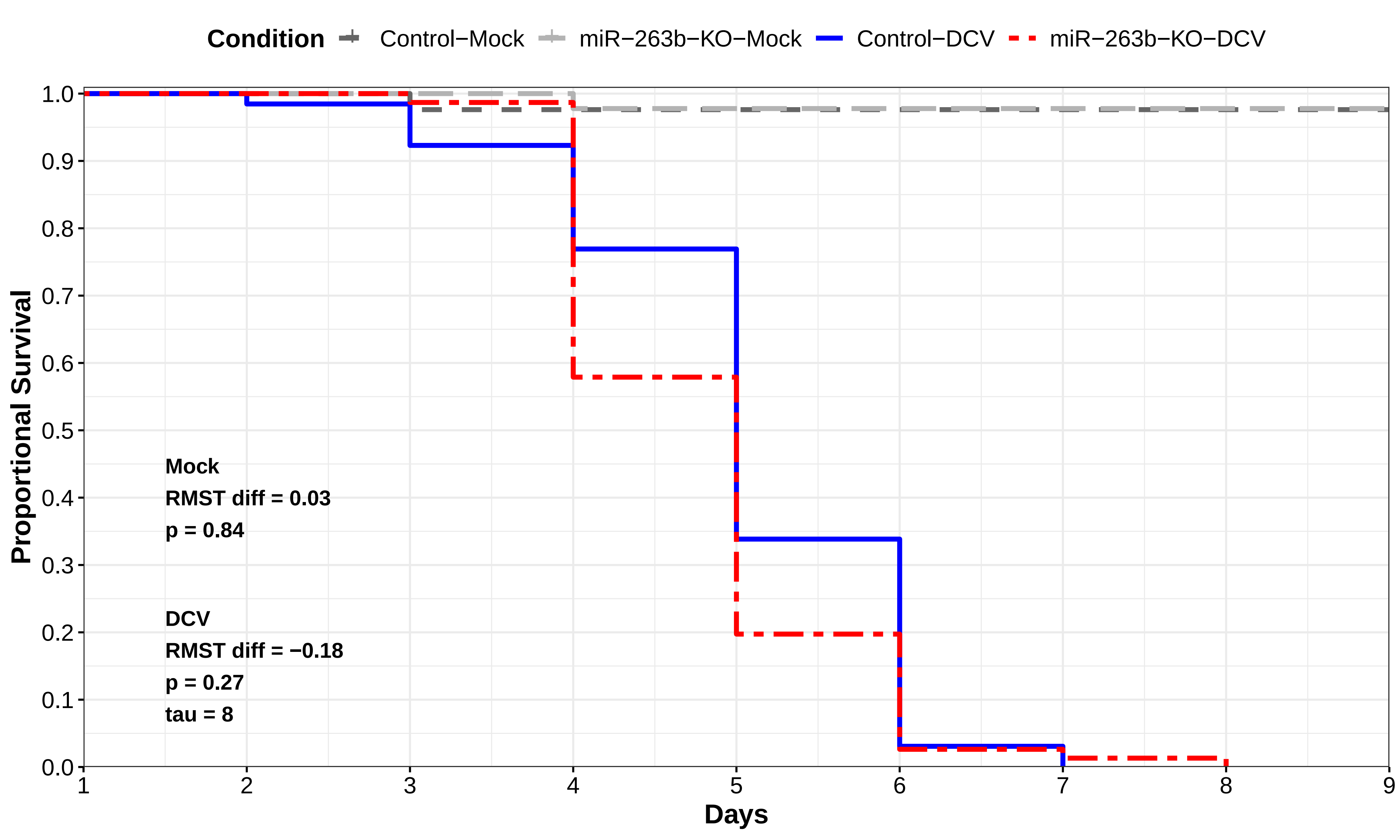
Condition
Control−Mock
miR−263b−KO−Mock
Control−DCV
miR−263b−KO−DCV
Proportional Survival
1.0
0.9
0.8
0.7
0.6
0.5
0.4
0.3
0.2
0.1
0.0
Mock
RMST diff = 0.03
p = 0.84
DCV
RMST diff = −0.18
p = 0.27
tau = 8
1
2
3
4
5
6
7
8
9
Days

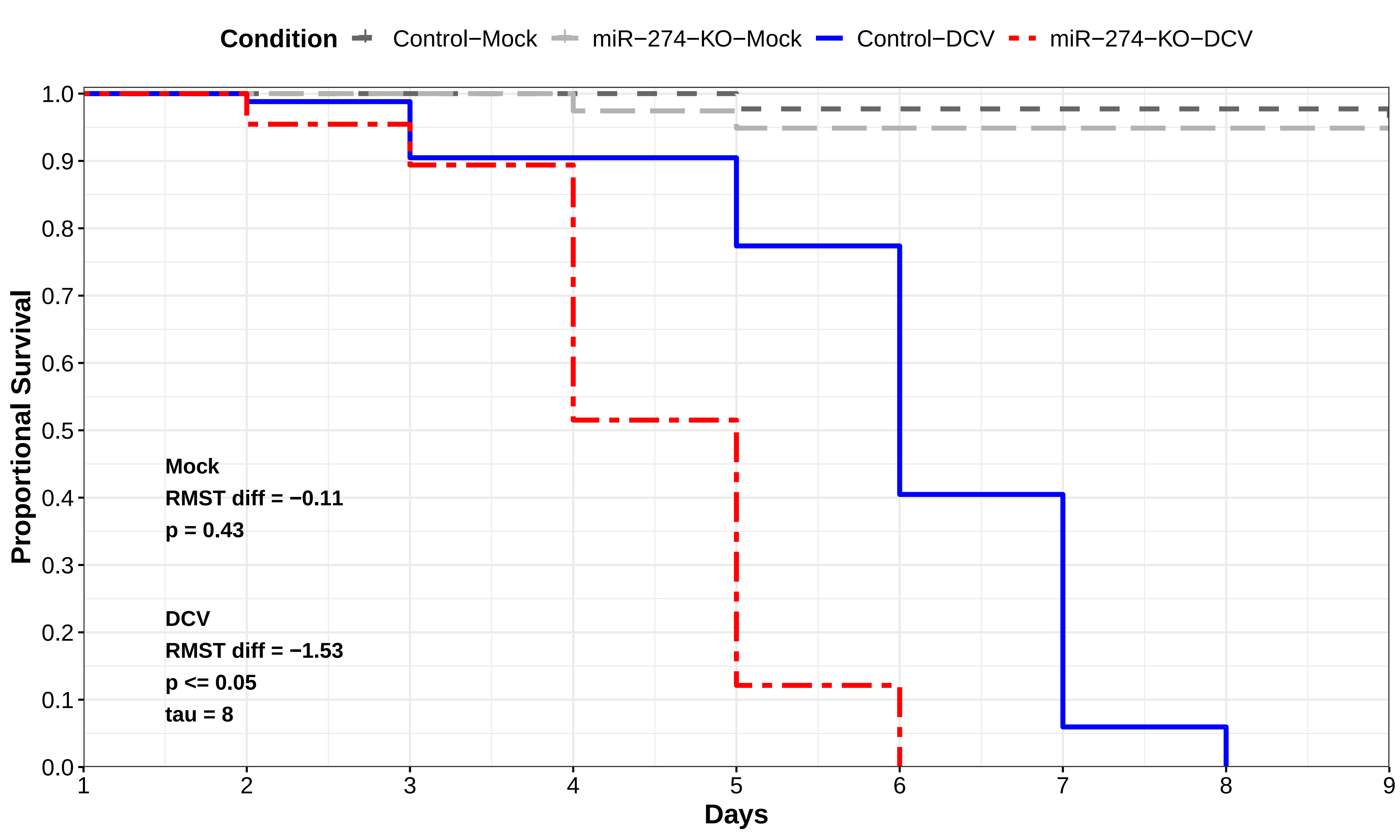

Condition
Control−Mock
miR−274−KO−Mock
Control−DCV
miR−274−KO−DCV
Proportional Survival
1.0
0.9
0.8
0.7
0.6
0.5
0.4
0.3
0.2
0.1
0.0
Mock
RMST diff = −0.11
p = 0.43
DCV
RMST diff = −1.53
p <= 0.05
tau = 8
1
2
3
4
5
6
7
8
9
Days

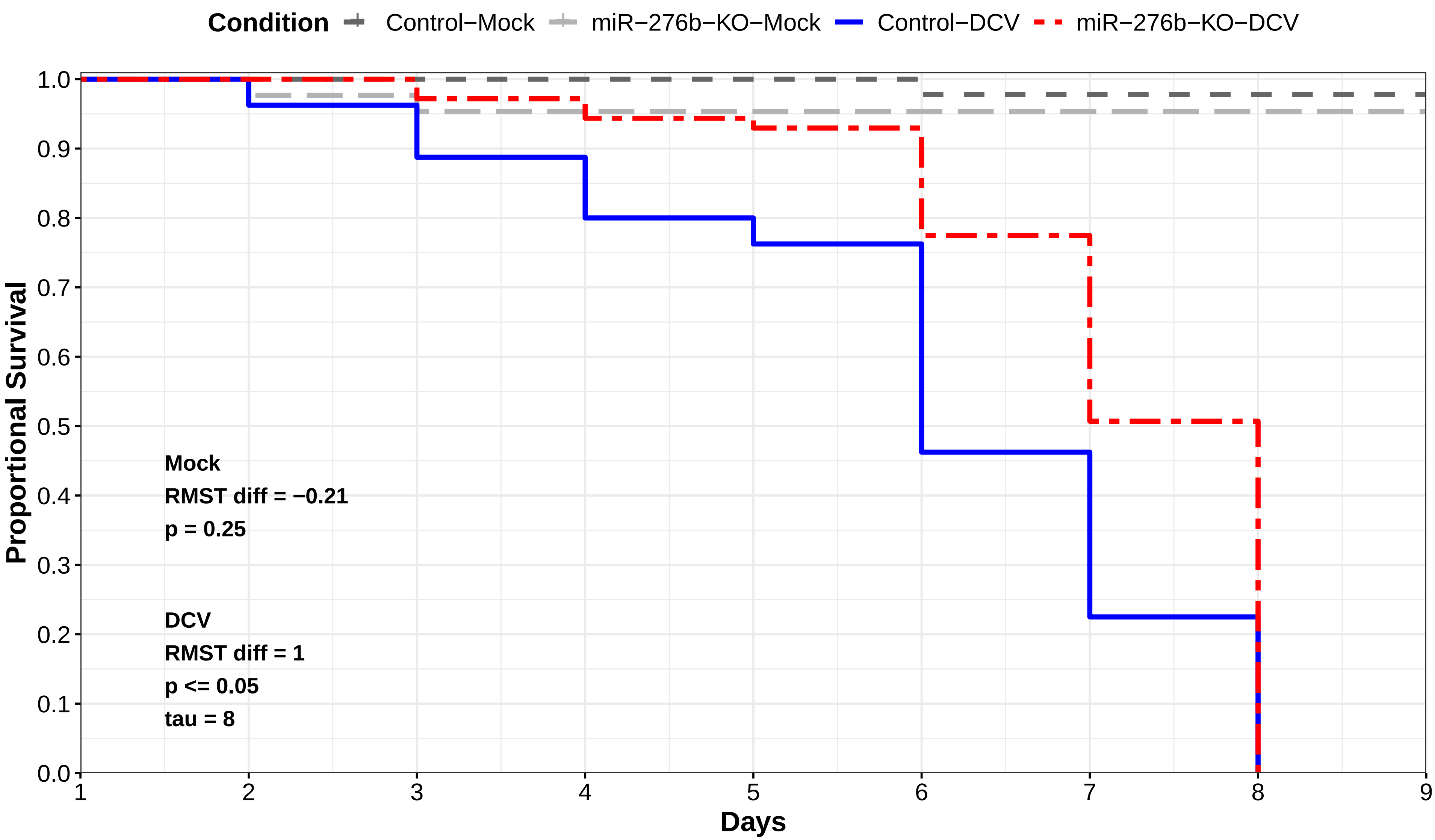
Condition
Control−Mock
miR−276b−KO−Mock
Control−DCV
miR−276b−KO−DCV
Proportional Survival
1.0
0.9
0.8
0.7
0.6
0.5
0.4
0.3
0.2
0.1
0.0
Mock
RMST diff = −0.21
p = 0.25
DCV
RMST diff = 1
p <= 0.05
tau = 8
1
2
3
4
5
6
7
8
9
Days

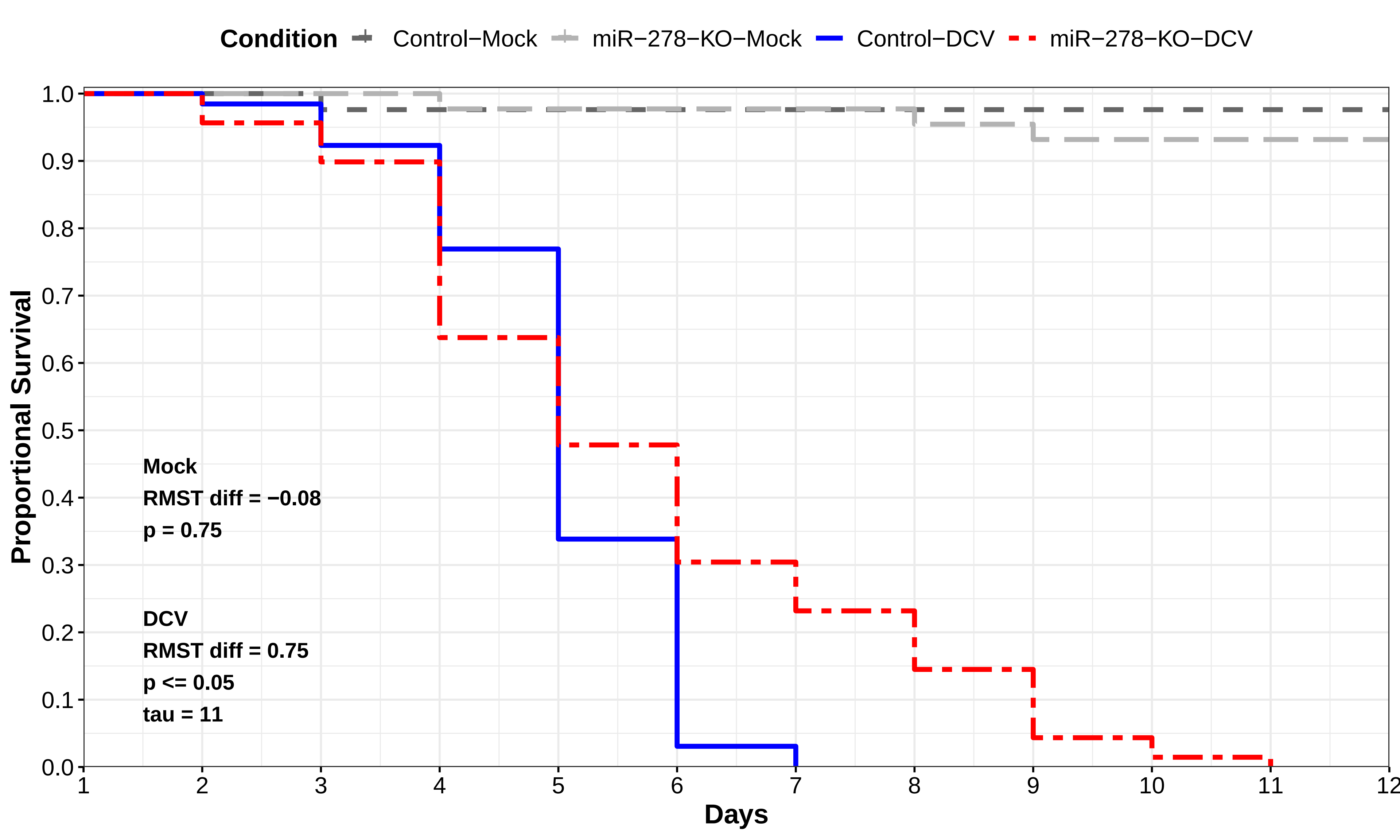
Condition
Control−Mock
miR−278−KO−Mock
Control−DCV
miR−278−KO−DCV
Proportional Survival
1.0
0.9
0.8
0.7
0.6
0.5
0.4
0.3
0.2
0.1
0.0
Mock
RMST diff = −0.08
p = 0.75
DCV
RMST diff = 0.75
p <= 0.05
tau = 11
1
2
3
4
5
6
7
8
9
10
11
12
Days

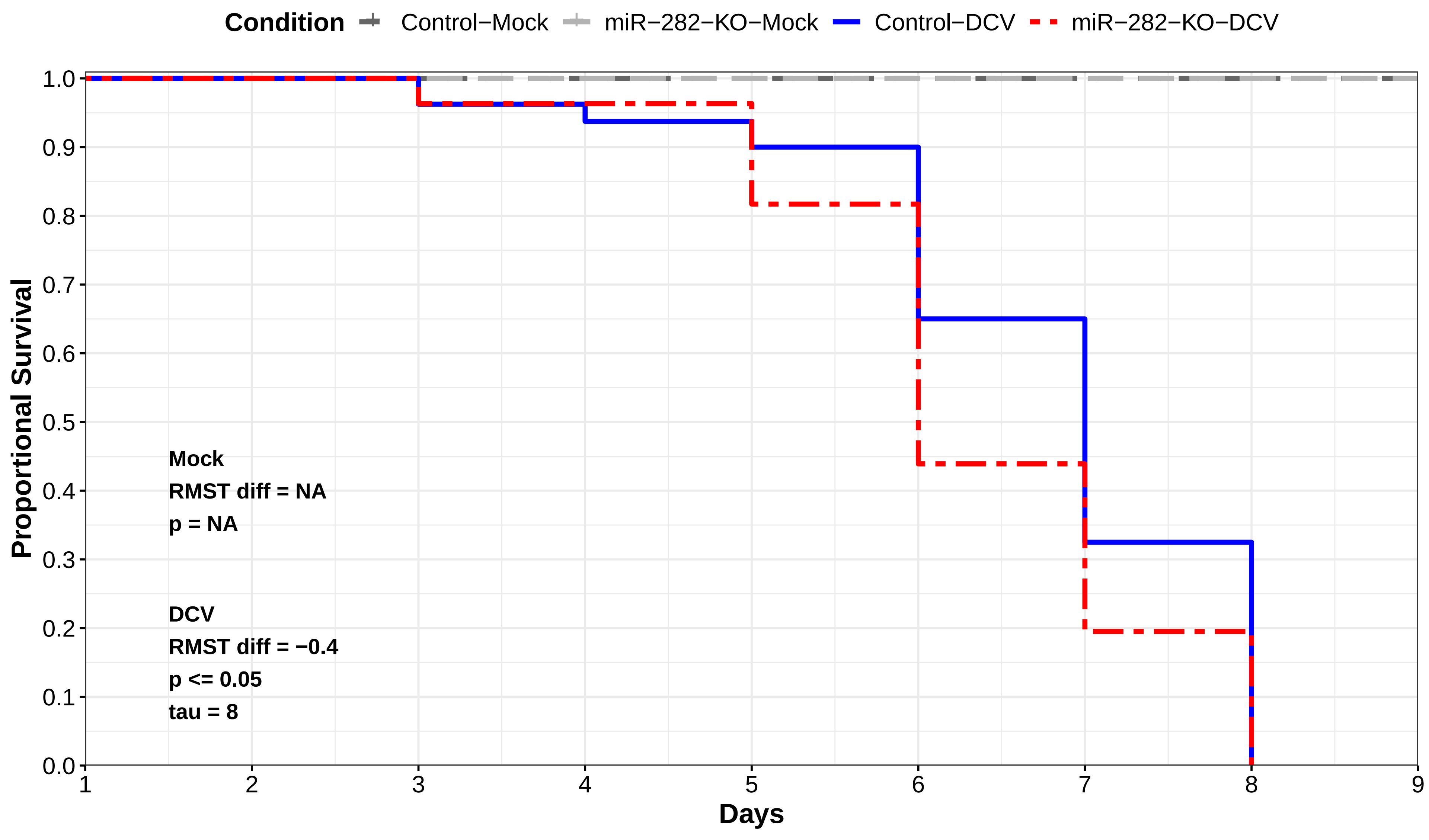

Condition
Control−Mock
miR−282−KO−Mock
Control−DCV
miR−282−KO−DCV
Proportional Survival
1.0
0.9
0.8
0.7
0.6
0.5
0.4
0.3
0.2
0.1
0.0
Mock
RMST diff = NA
p = NA
DCV
RMST diff = −0.4
p <= 0.05
tau = 8
1
2
3
4
5
6
7
8
9
Days

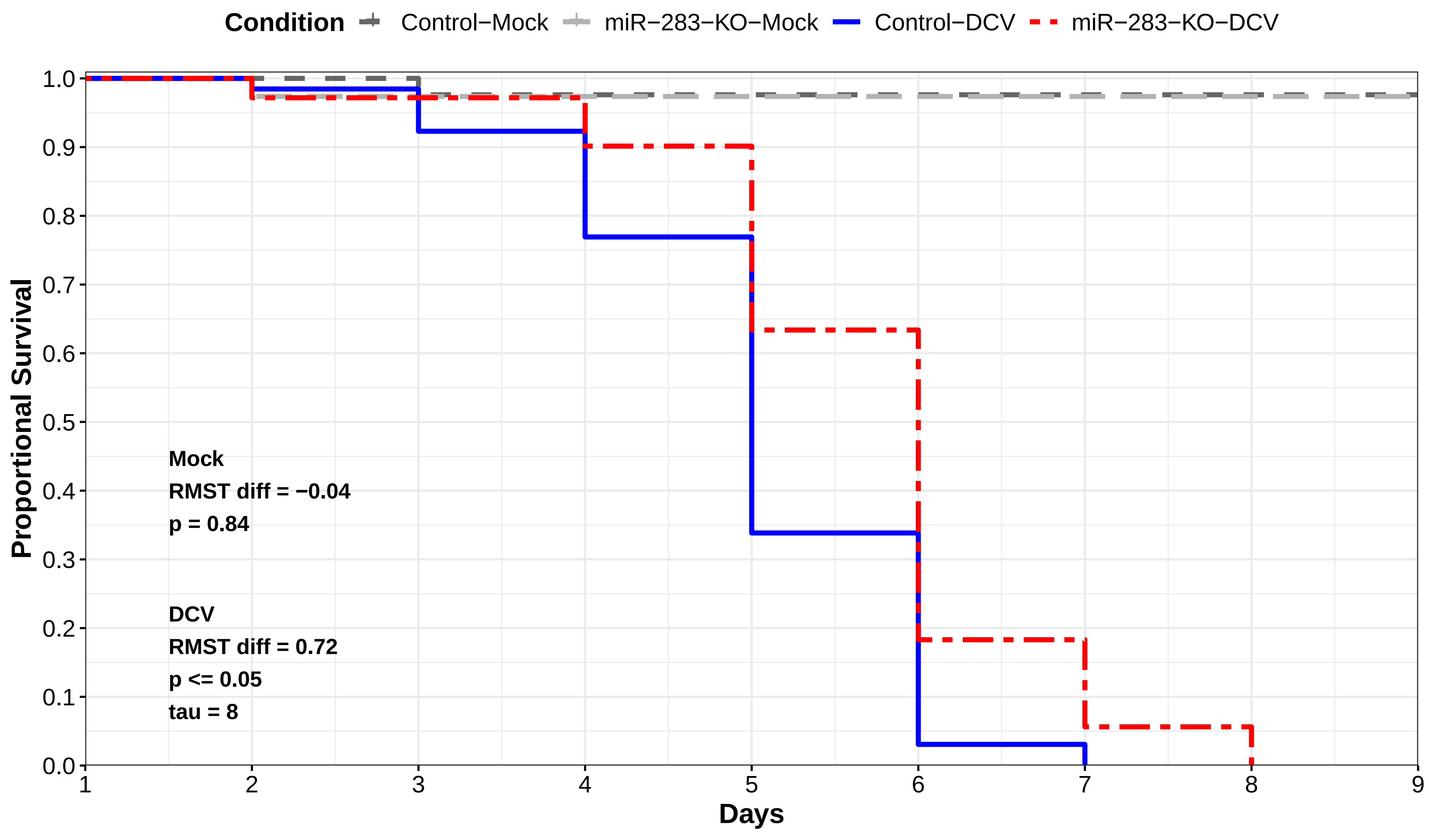

Condition
Control−Mock
miR−283−KO−Mock
Control−DCV
miR−283−KO−DCV
Proportional Survival
1.0
0.9
0.8
0.7
0.6
0.5
0.4
0.3
0.2
0.1
0.0
Mock
RMST diff = −0.04
p = 0.84
DCV
RMST diff = 0.72
p <= 0.05
tau = 8
1
2
3
4
5
6
7
8
9
Days

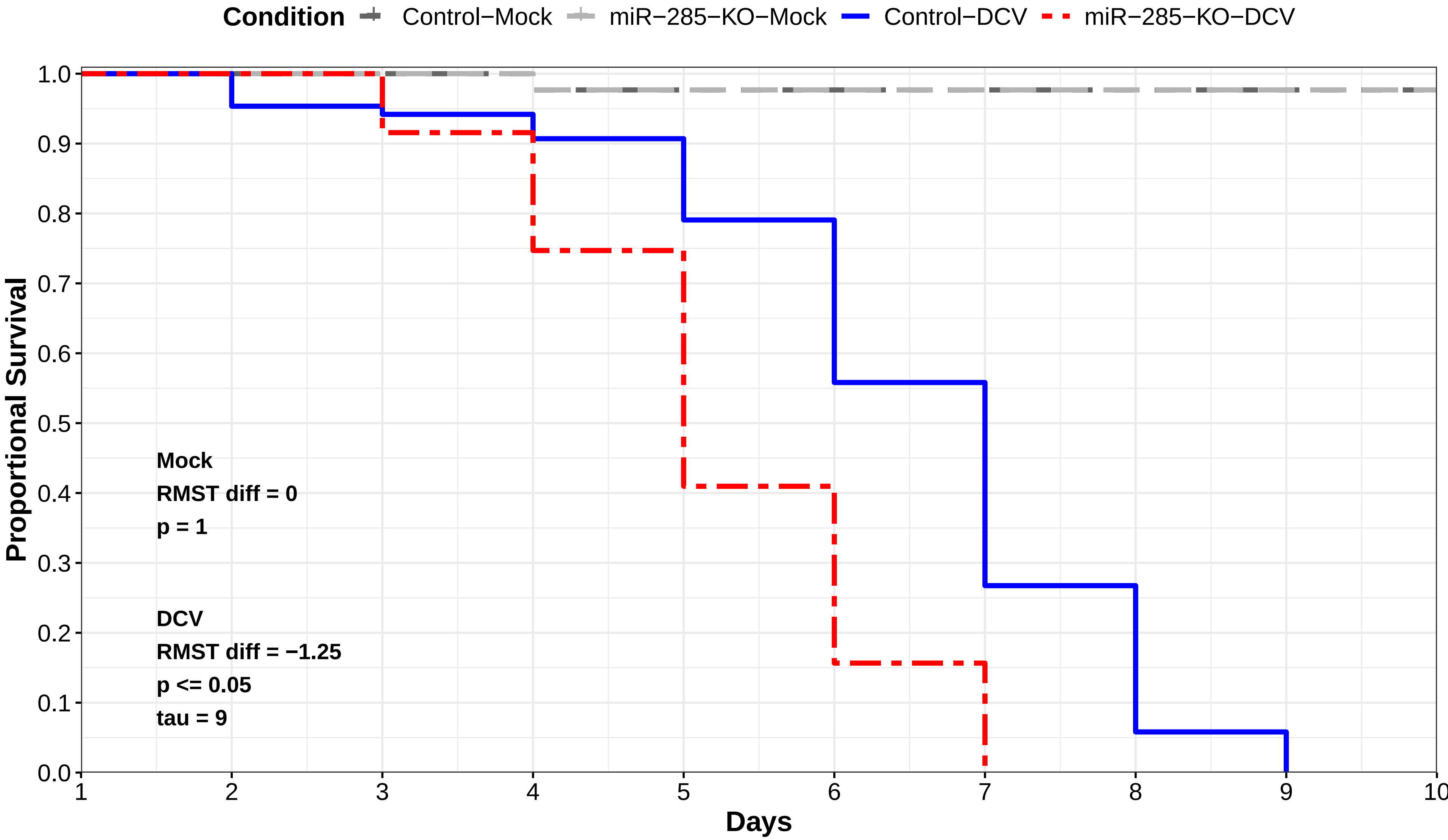
Condition
Control−Mock
miR−285−KO−Mock
Control−DCV
miR−285−KO−DCV
Proportional Survival
1.0
0.9
0.8
0.7
0.6
0.5
0.4
0.3
0.2
0.1
0.0
Mock
RMST diff = 0
p = 1
DCV
RMST diff = −1.25
p <= 0.05
tau = 9
1
2
3
4
5
6
7
8
9
10
Days

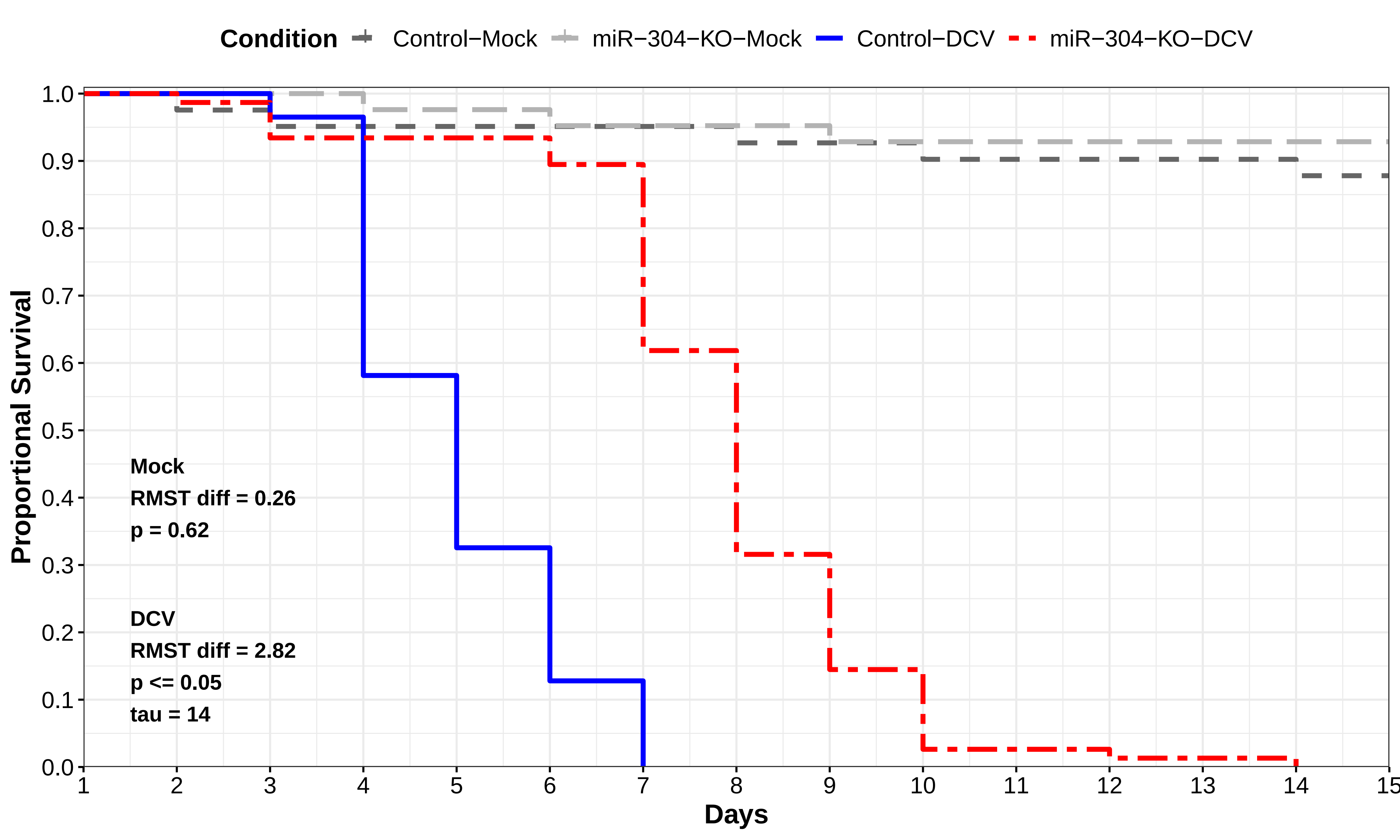

Condition
Control−Mock
miR−304−KO−Mock
Control−DCV
miR−304−KO−DCV
Proportional Survival
Days
Mock
RMST diff = 0.26
p = 0.62
DCV
RMST diff = 2.82
p <= 0.05
tau = 14

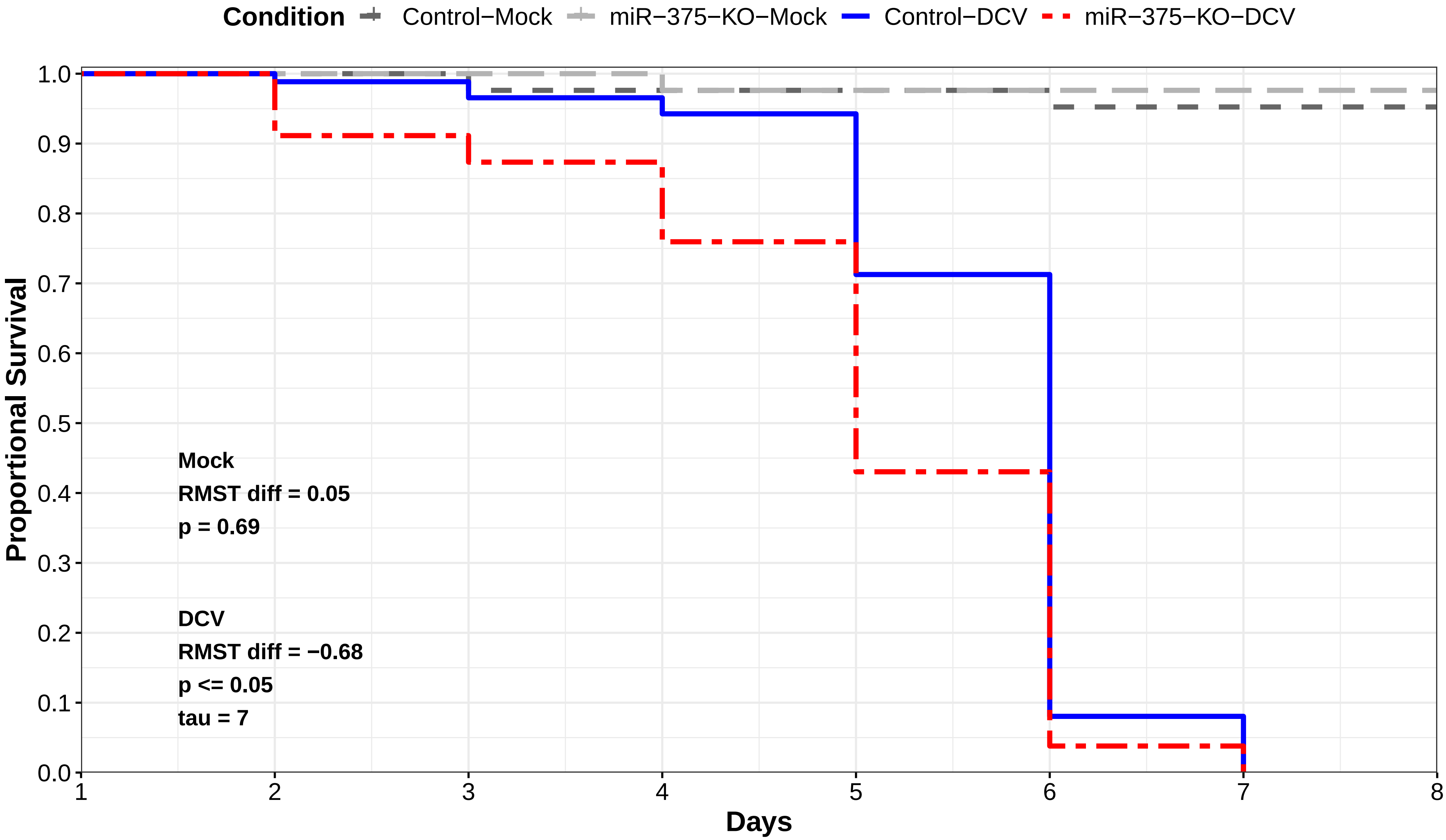
Condition
Control−Mock
miR−375−KO−Mock
Control−DCV
miR−375−KO−DCV
Proportional Survival
1.0
0.9
0.8
0.7
0.6
0.5
0.4
0.3
0.2
0.1
0.0
Days
1
2
3
4
5
6
7
8
Mock
RMST diff = 0.05
p = 0.69
DCV
RMST diff = −0.68
p <= 0.05
tau = 7

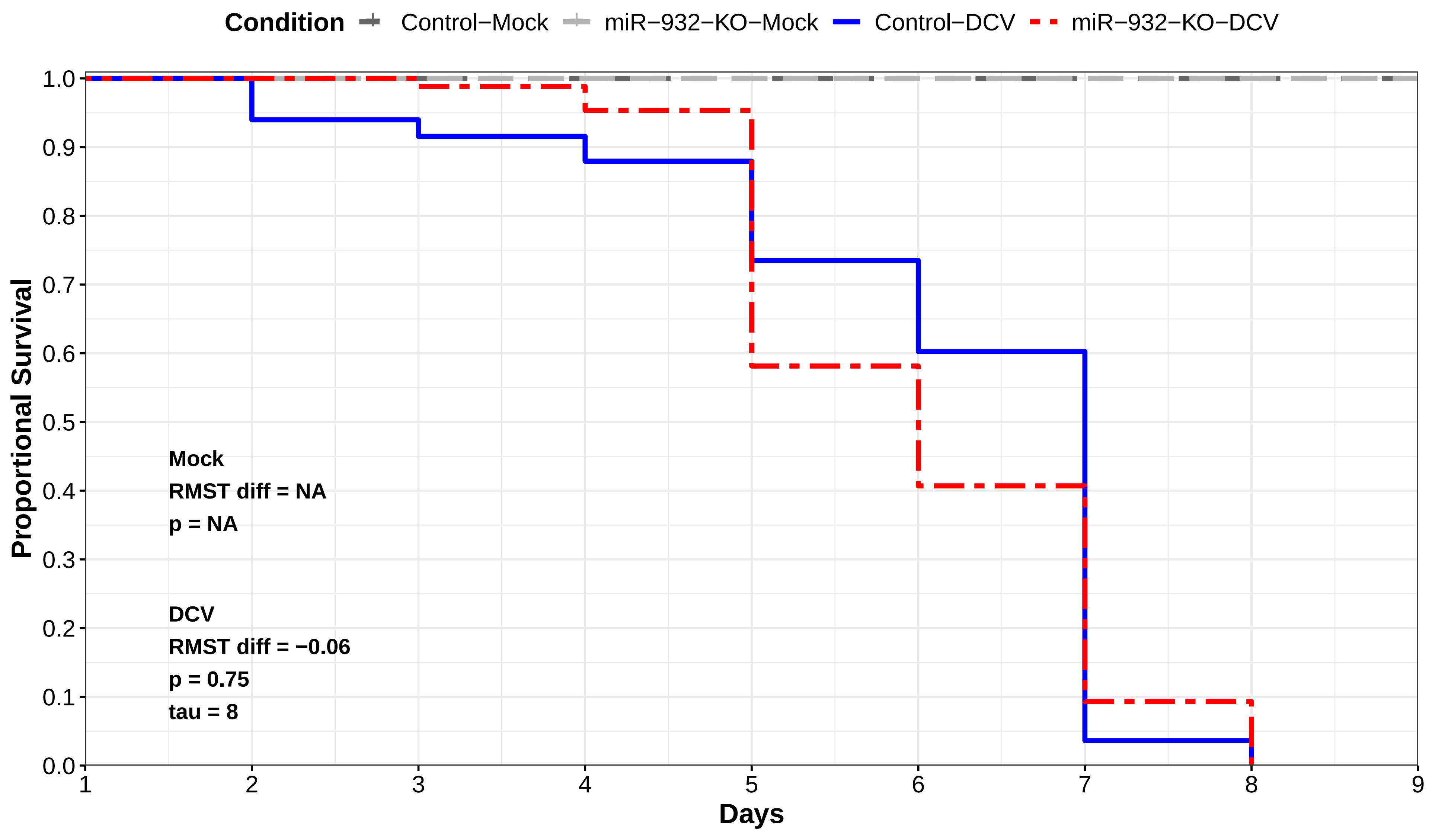

Condition
Control−Mock
miR−932−KO−Mock
Control−DCV
miR−932−KO−DCV
Proportional Survival
1.0
0.9
0.8
0.7
0.6
0.5
0.4
0.3
0.2
0.1
0.0
Mock
RMST diff = NA
p = NA
DCV
RMST diff = −0.06
p = 0.75
tau = 8
1
2
3
4
5
6
7
8
9
Days

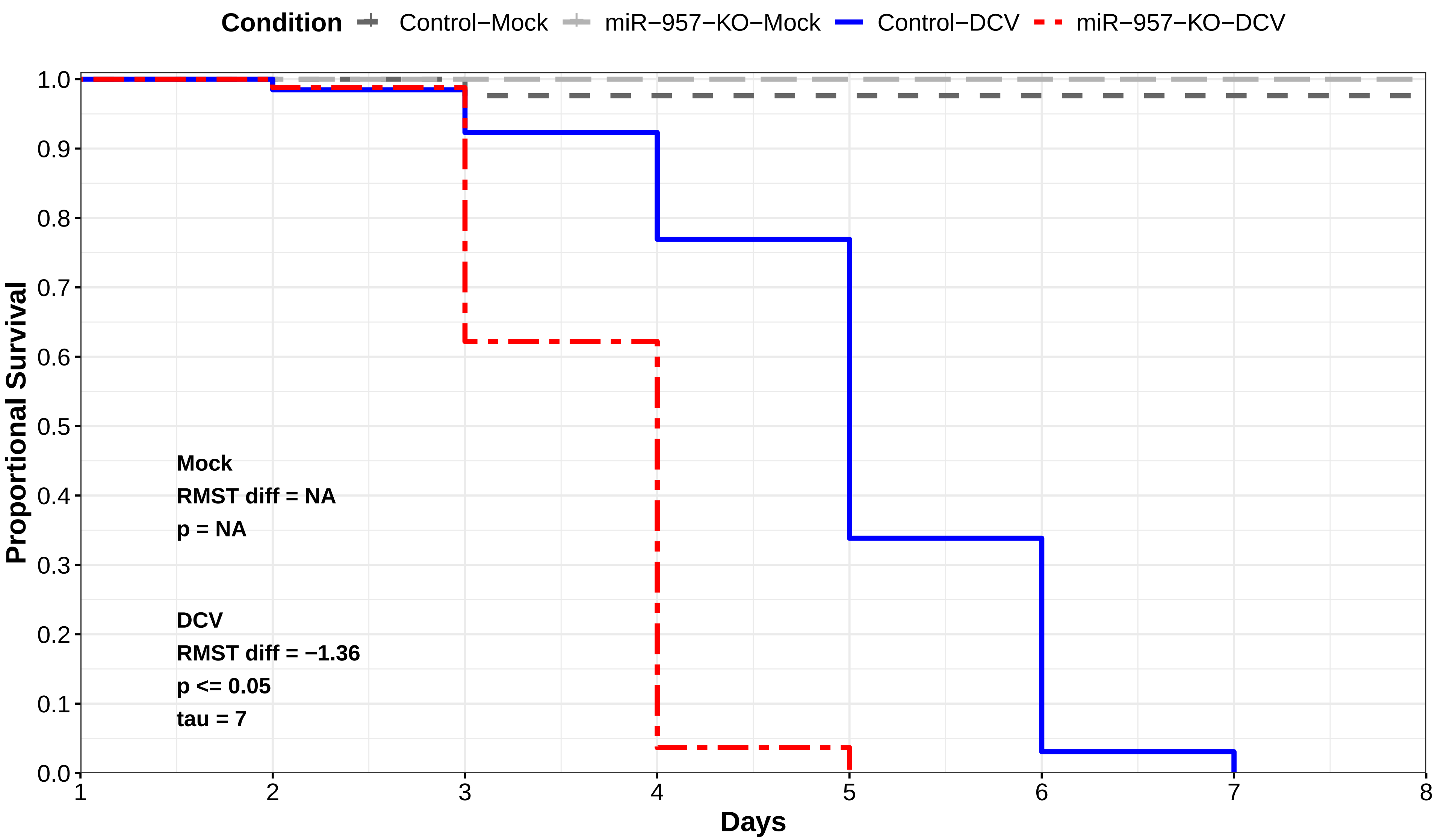
Condition
Control−Mock
miR−957−KO−Mock
Control−DCV
miR−957−KO−DCV
Proportional Survival
1.0
0.9
0.8
0.7
0.6
0.5
0.4
0.3
0.2
0.1
0.0
Mock
RMST diff = NA
p = NA
DCV
RMST diff = −1.36
p <= 0.05
tau = 7
1
2
3
4
5
6
7
8
Days

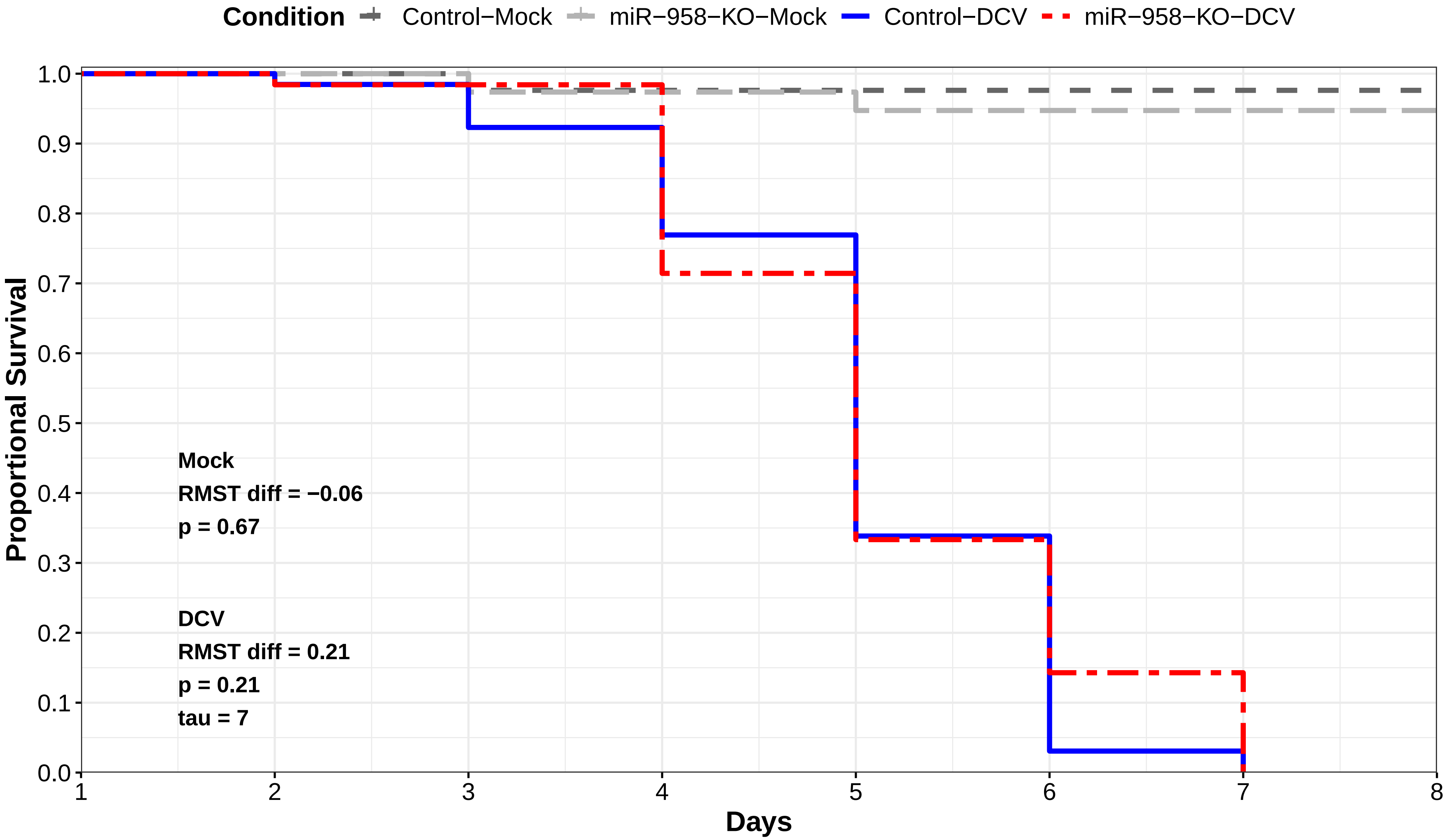

Condition
Control−Mock
miR−958−KO−Mock
Control−DCV
miR−958−KO−DCV
Proportional Survival
1.0
0.9
0.8
0.7
0.6
0.5
0.4
0.3
0.2
0.1
0.0
Mock
RMST diff = −0.06
p = 0.67
DCV
RMST diff = 0.21
p = 0.21
tau = 7
1
2
3
4
5
6
7
8
Days

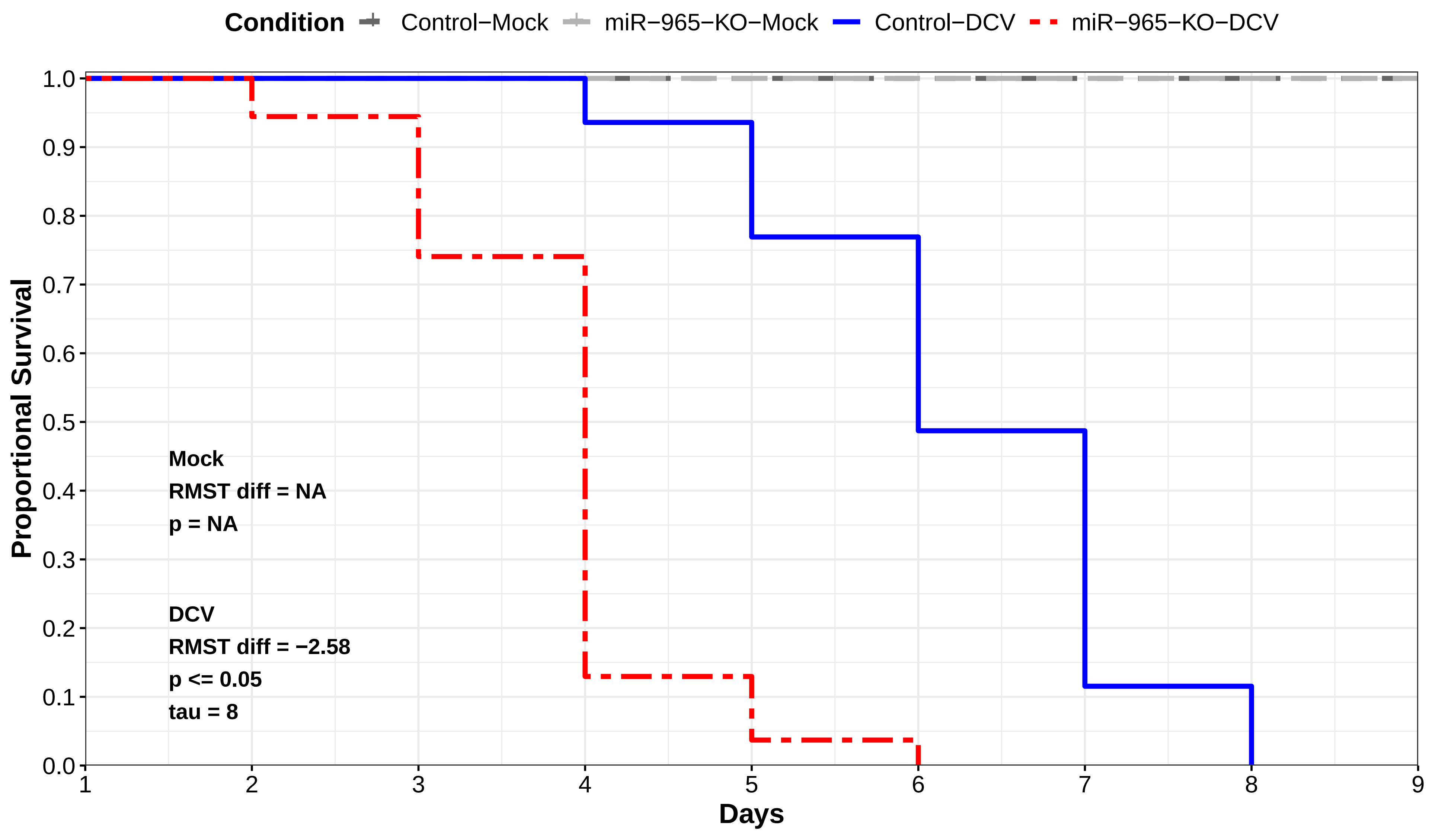

Condition
Control−Mock
miR−965−KO−Mock
Control−DCV
miR−965−KO−DCV
Proportional Survival
1.0
0.9
0.8
0.7
0.6
0.5
0.4
0.3
0.2
0.1
0.0
Mock
RMST diff = NA
p = NA
DCV
RMST diff = −2.58
p <= 0.05
tau = 8
1
2
3
4
5
6
7
8
9
Days

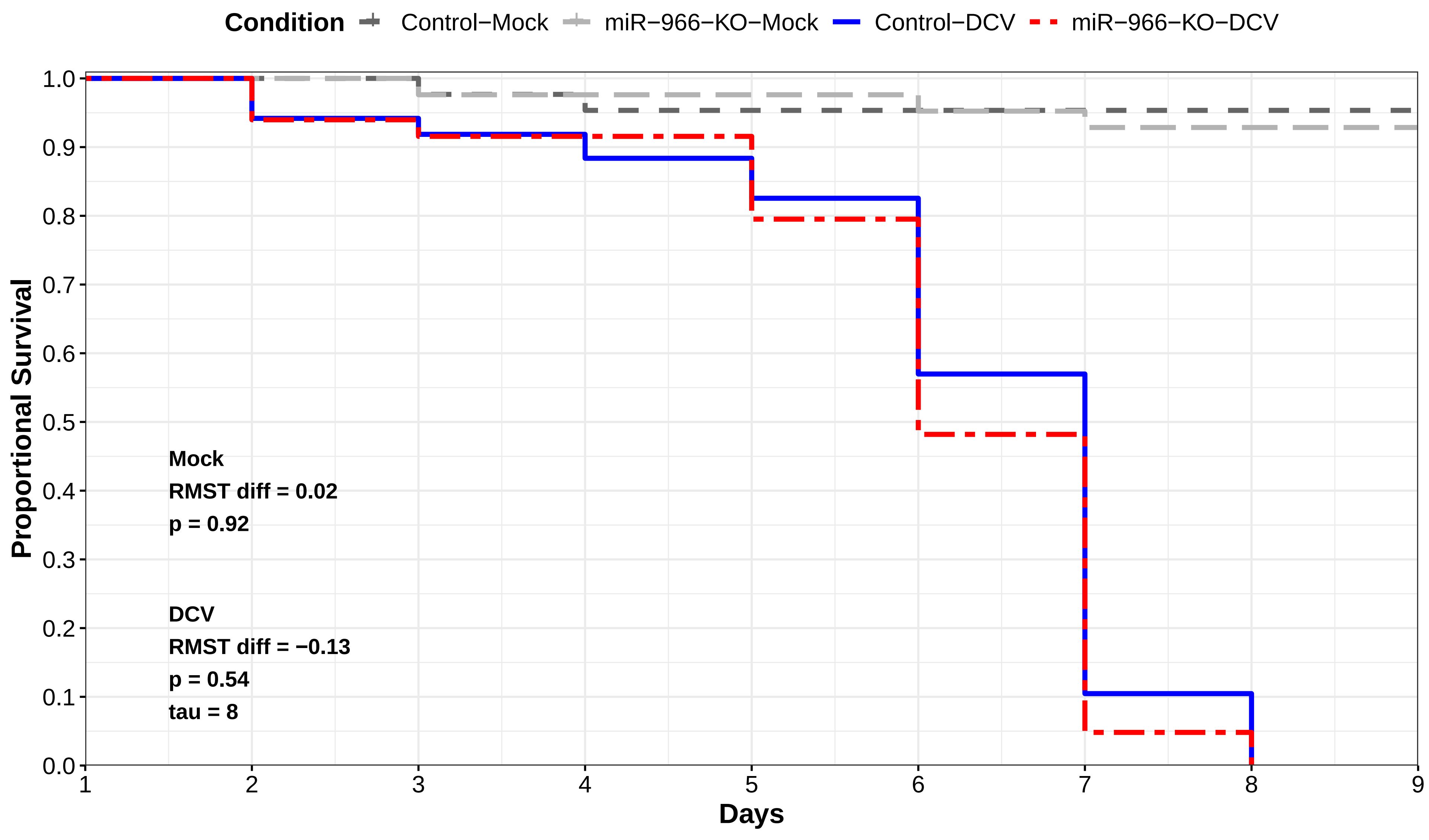

Condition
Control−Mock
miR−966−KO−Mock
Control−DCV
miR−966−KO−DCV
Proportional Survival
Days
Mock
RMST diff = 0.02
p = 0.92
DCV
RMST diff = −0.13
p = 0.54
tau = 8

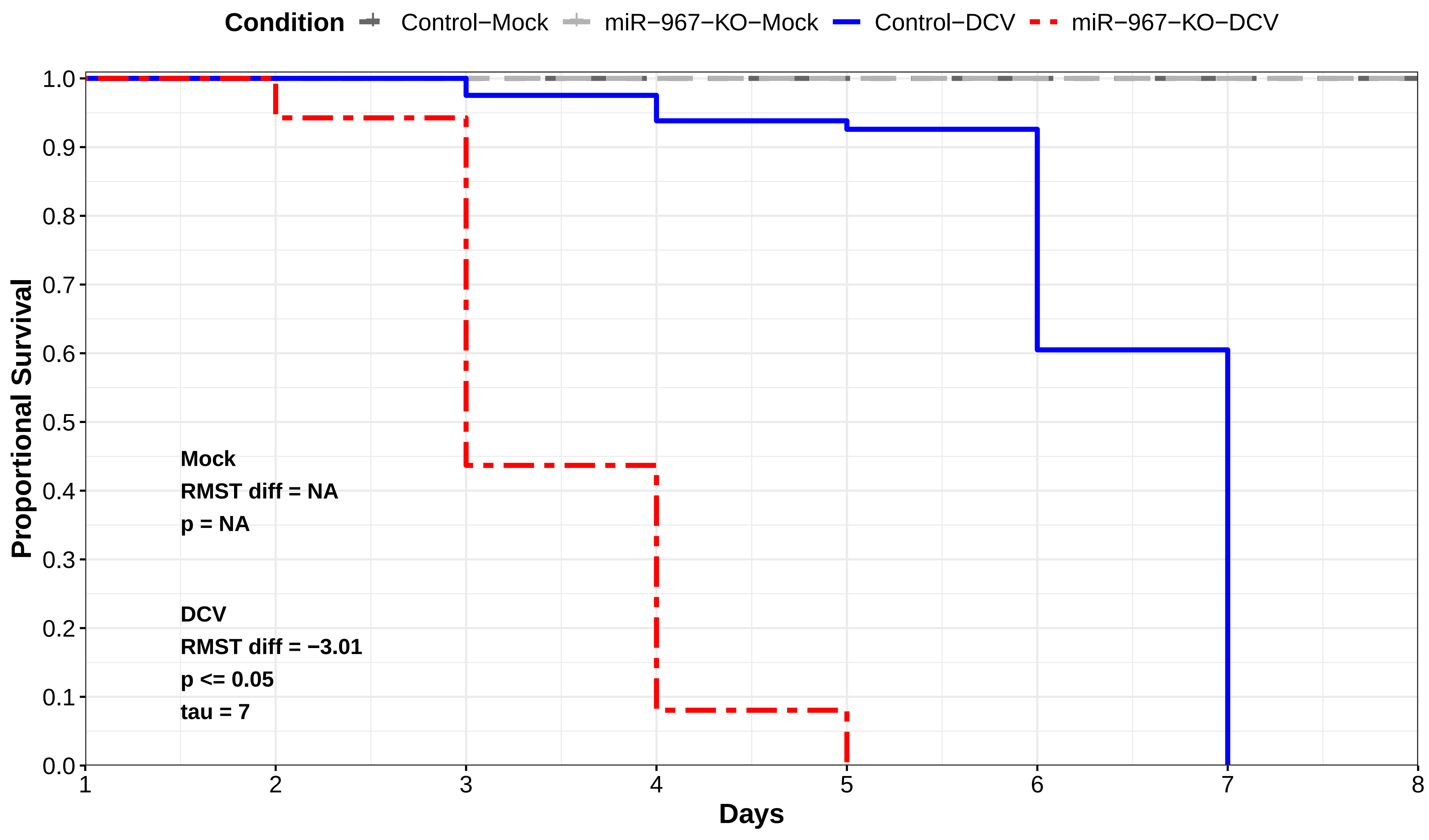

Condition
Control−Mock
miR−967−KO−Mock
Control−DCV
miR−967−KO−DCV
Proportional Survival
1.0
0.9
0.8
0.7
0.6
0.5
0.4
0.3
0.2
0.1
0.0
Mock
RMST diff = NA
p = NA
DCV
RMST diff = −3.01
p <= 0.05
tau = 7
1
2
3
4
5
6
7
8
Days

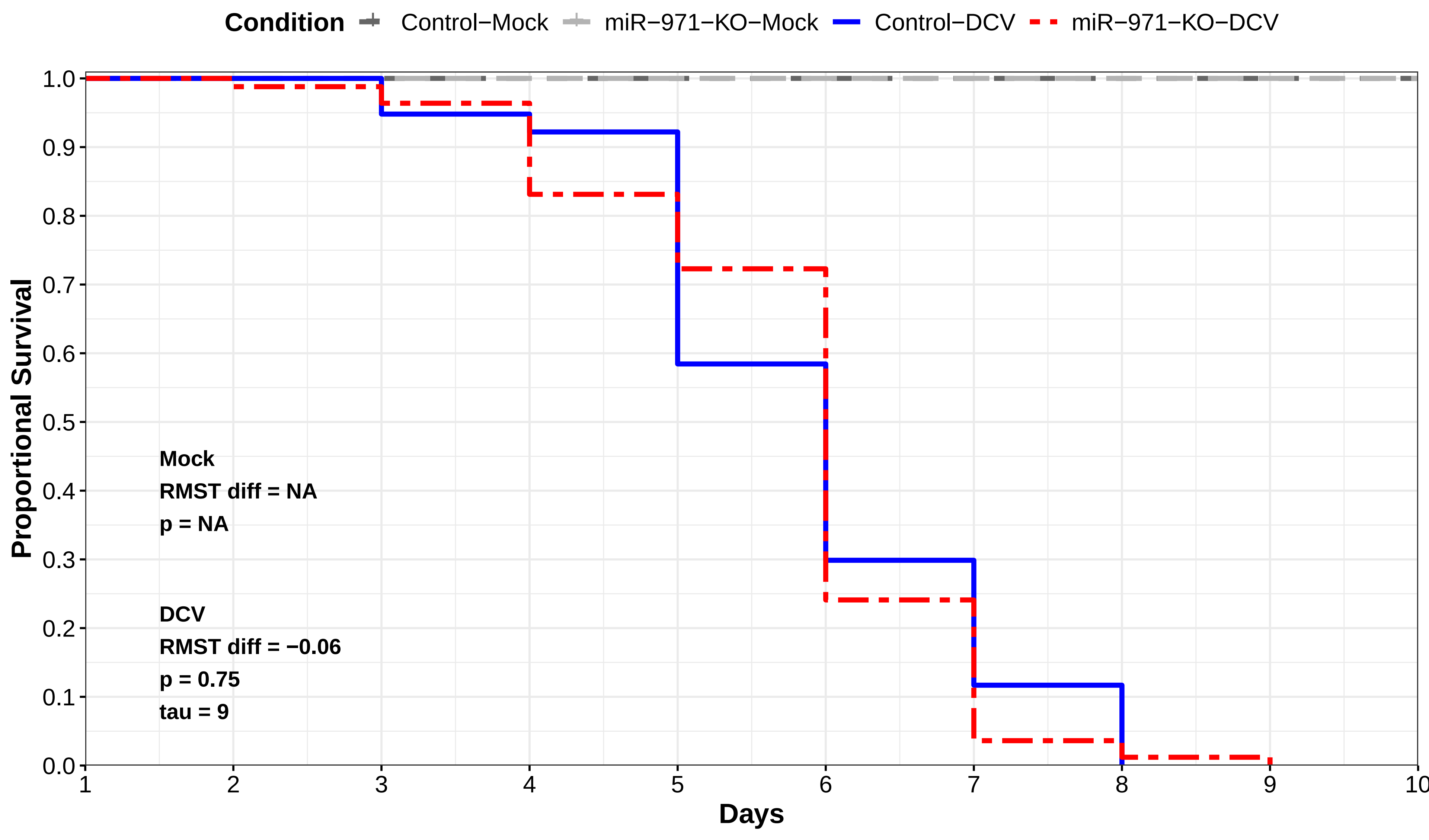

Condition
Control−Mock
miR−971−KO−Mock
Control−DCV
miR−971−KO−DCV
Proportional Survival
1.0
0.9
0.8
0.7
0.6
0.5
0.4
0.3
0.2
0.1
0.0
Mock
RMST diff = NA
p = NA
DCV
RMST diff = −0.06
p = 0.75
tau = 9
1
2
3
4
5
6
7
8
9
10
Days

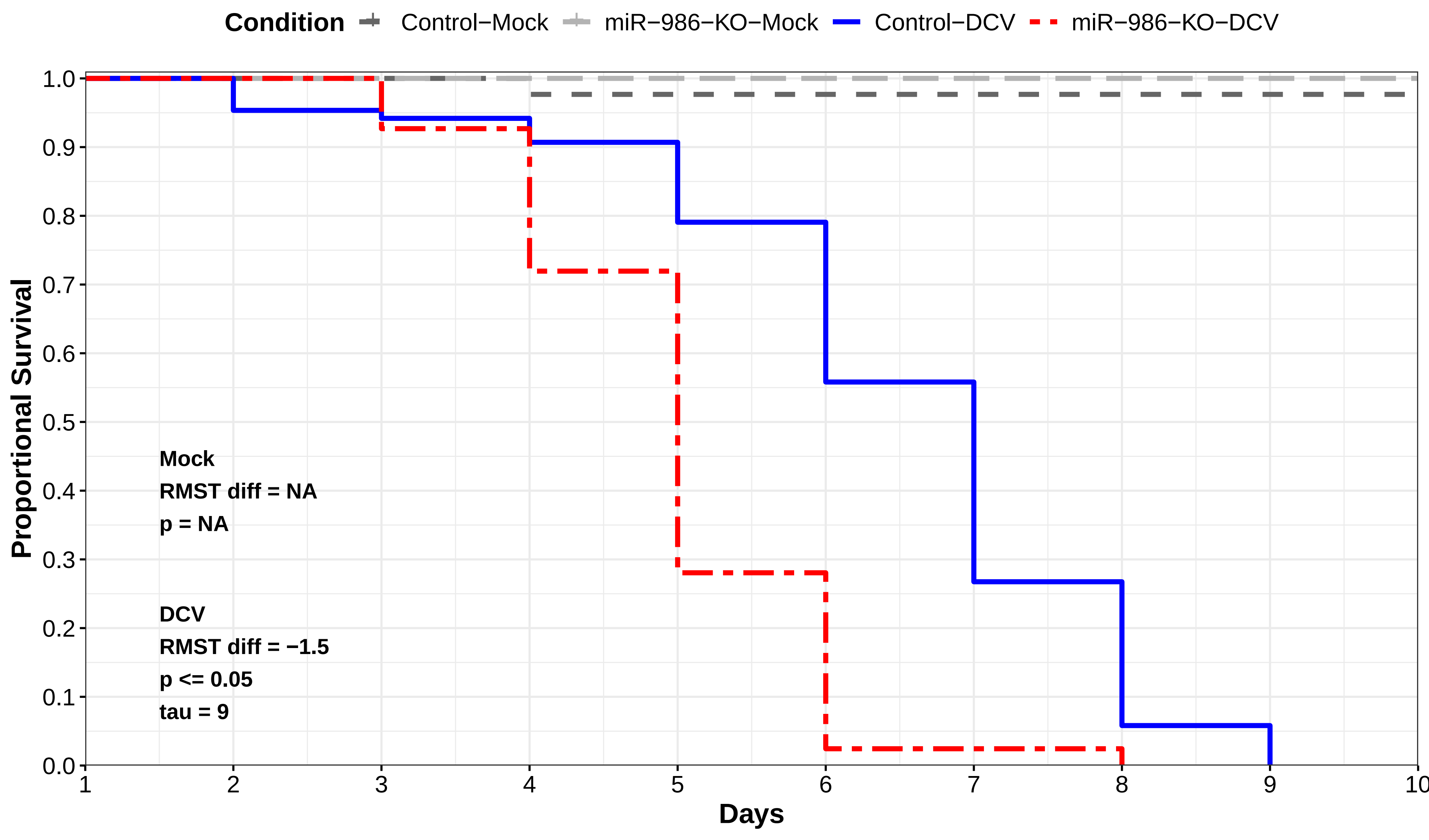
Condition
Control−Mock
miR−986−KO−Mock
Control−DCV
miR−986−KO−DCV
Proportional Survival
1.0
0.9
0.8
0.7
0.6
0.5
0.4
0.3
0.2
0.1
0.0
Mock
RMST diff = NA
p = NA
DCV
RMST diff = −1.5
p <= 0.05
tau = 9
1
2
3
4
5
6
7
8
9
10
Days

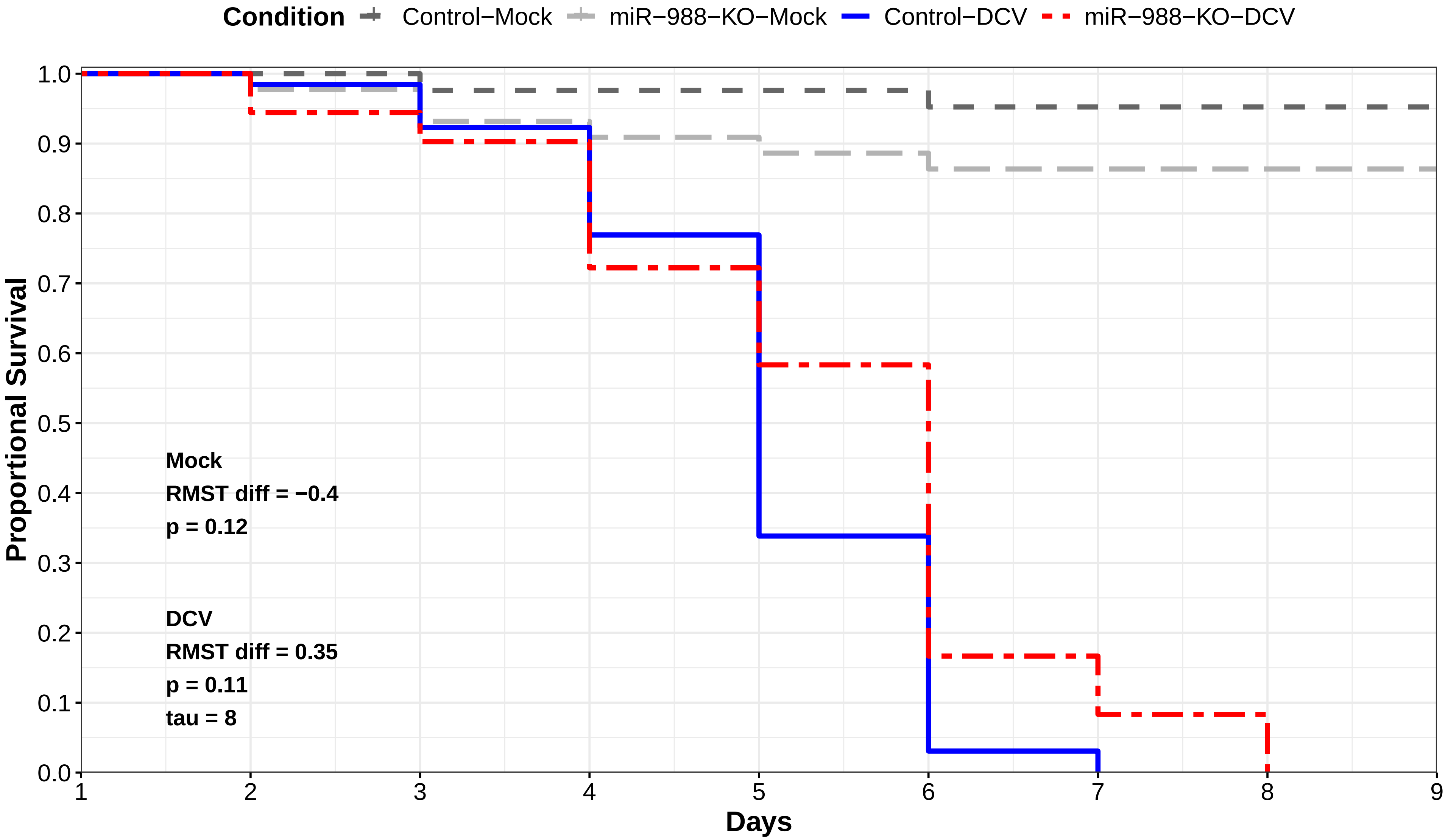
Condition
Control−Mock
miR−988−KO−Mock
Control−DCV
miR−988−KO−DCV
Proportional Survival
1.0
0.9
0.8
0.7
0.6
0.5
0.4
0.3
0.2
0.1
0.0
Mock
RMST diff = −0.4
p = 0.12
DCV
RMST diff = 0.35
p = 0.11
tau = 8
1
2
3
4
5
6
7
8
9
Days

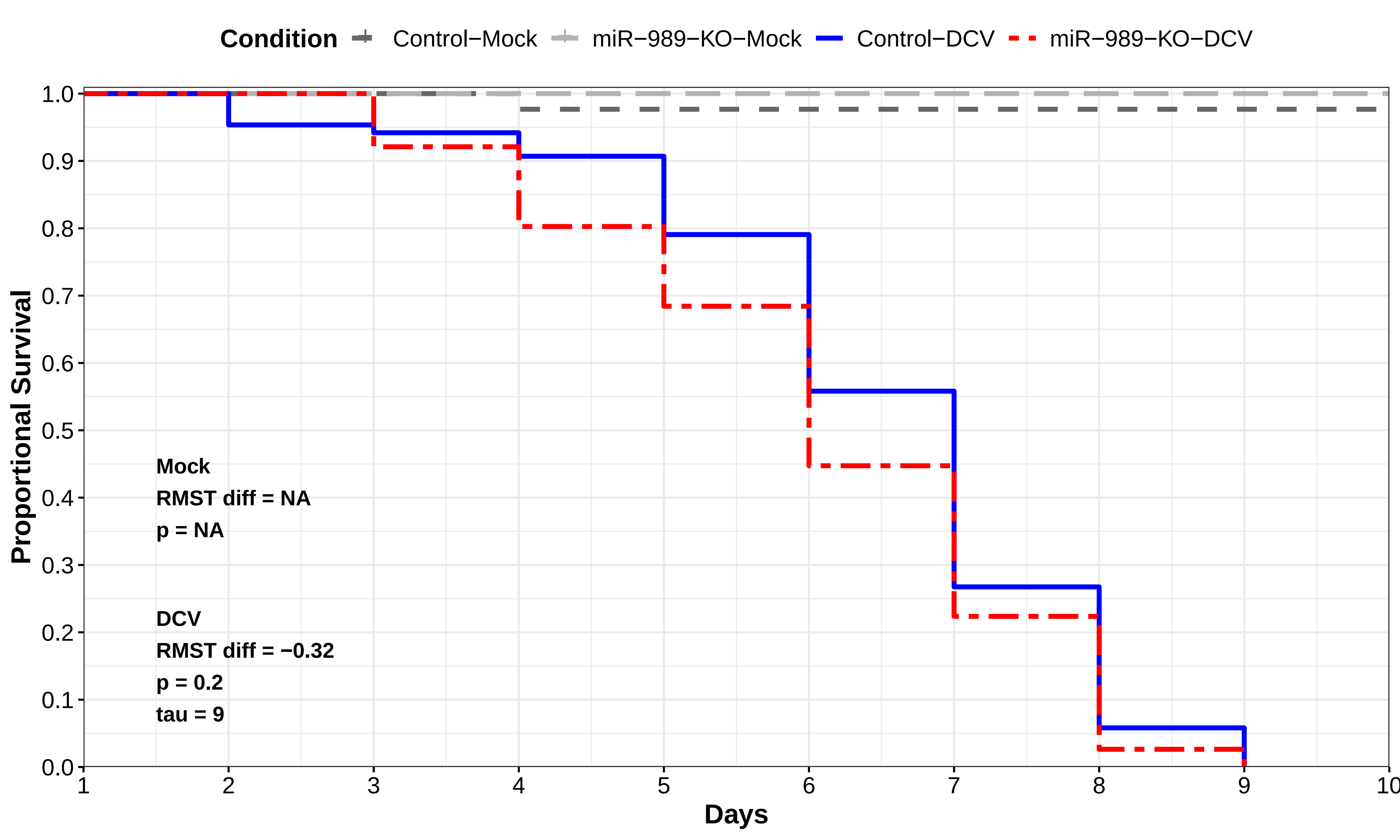
Condition
Control−Mock
miR−989−KO−Mock
Control−DCV
miR−989−KO−DCV
Proportional Survival
1.0
0.9
0.8
0.7
0.6
0.5
0.4
0.3
0.2
0.1
0.0
Mock
RMST diff = NA
p = NA
DCV
RMST diff = −0.32
p = 0.2
tau = 9
1
2
3
4
5
6
7
8
9
10
Days

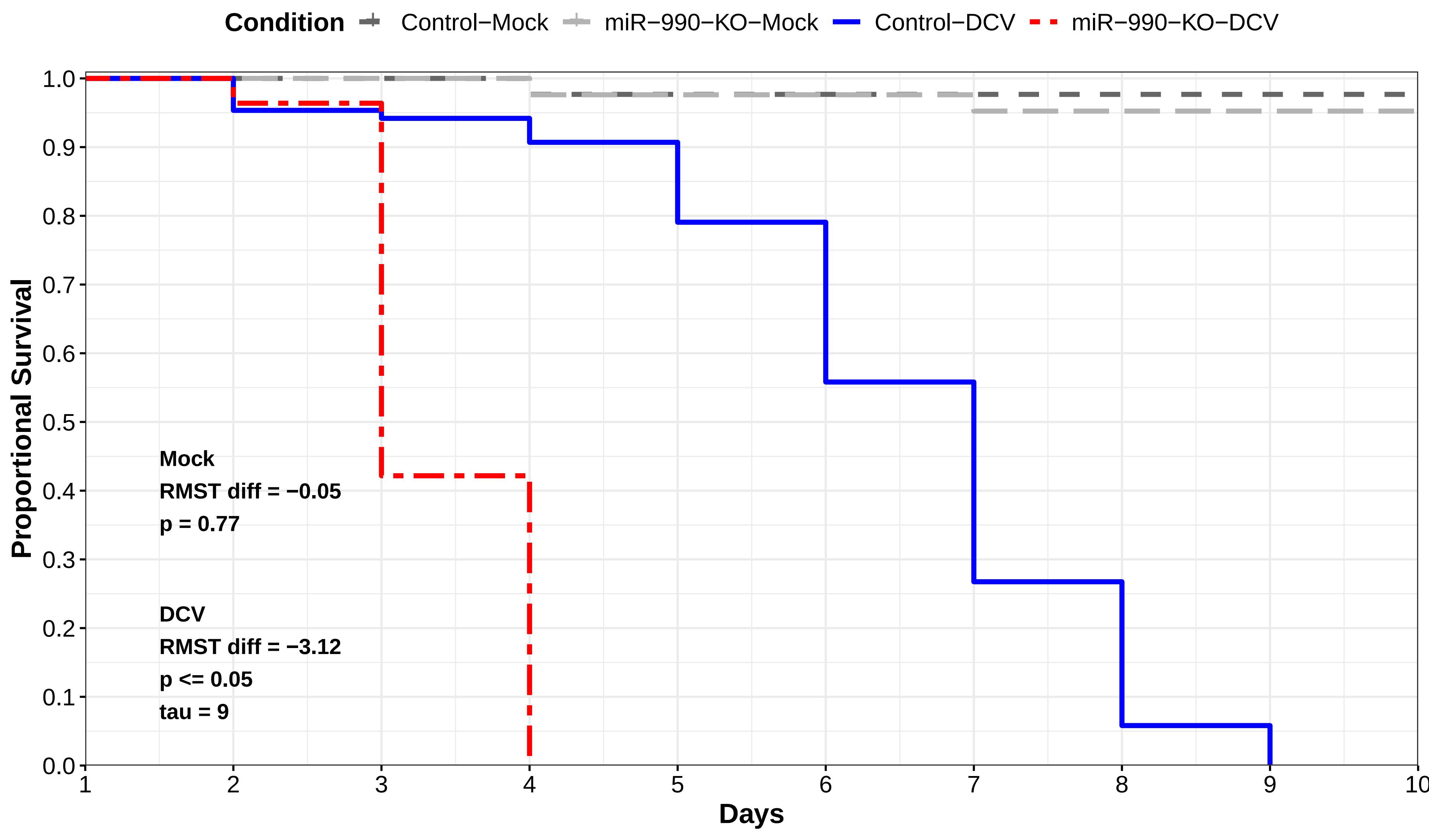

Condition
Control−Mock
miR−990−KO−Mock
Control−DCV
miR−990−KO−DCV
Proportional Survival
1.0
0.9
0.8
0.7
0.6
0.5
0.4
0.3
0.2
0.1
0.0
Mock
RMST diff = −0.05
p = 0.77
DCV
RMST diff = −3.12
p <= 0.05
tau = 9
1
2
3
4
5
6
7
8
9
10
Days

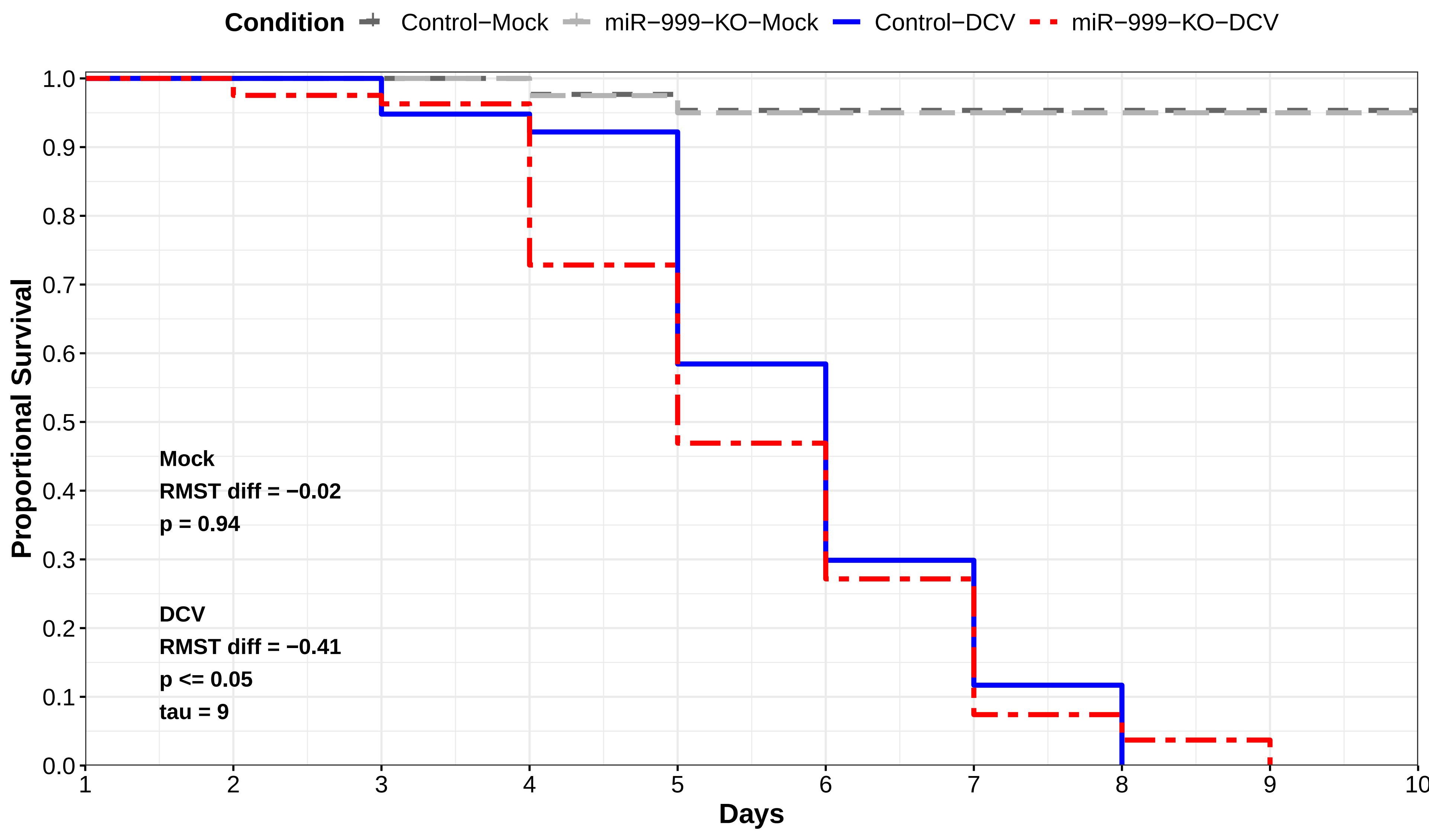
Condition
Control−Mock
miR−999−KO−Mock
Control−DCV
miR−999−KO−DCV
Proportional Survival
1.0
0.9
0.8
0.7
0.6
0.5
0.4
0.3
0.2
0.1
0.0
Days
1
2
3
4
5
6
7
8
9
10
Mock
RMST diff = −0.02
p = 0.94
DCV
RMST diff = −0.41
p <= 0.05
tau = 9

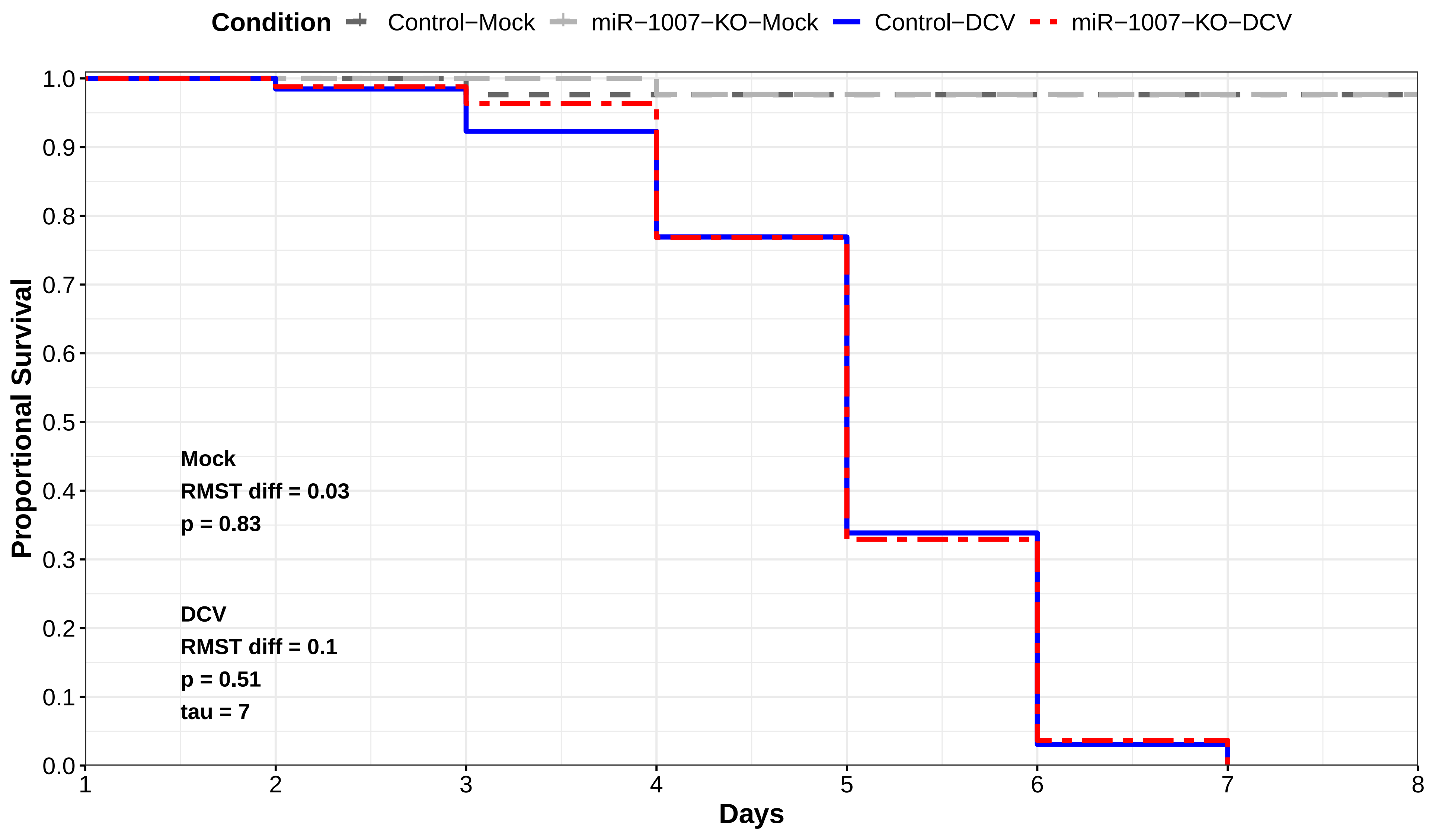

Condition
Control−Mock
miR−1007−KO−Mock
Control−DCV
miR−1007−KO−DCV
Proportional Survival
1.0
0.9
0.8
0.7
0.6
0.5
0.4
0.3
0.2
0.1
0.0
Days
1
2
3
4
5
6
7
8
Mock
RMST diff = 0.03
p = 0.83
DCV
RMST diff = 0.1
p = 0.51
tau = 7

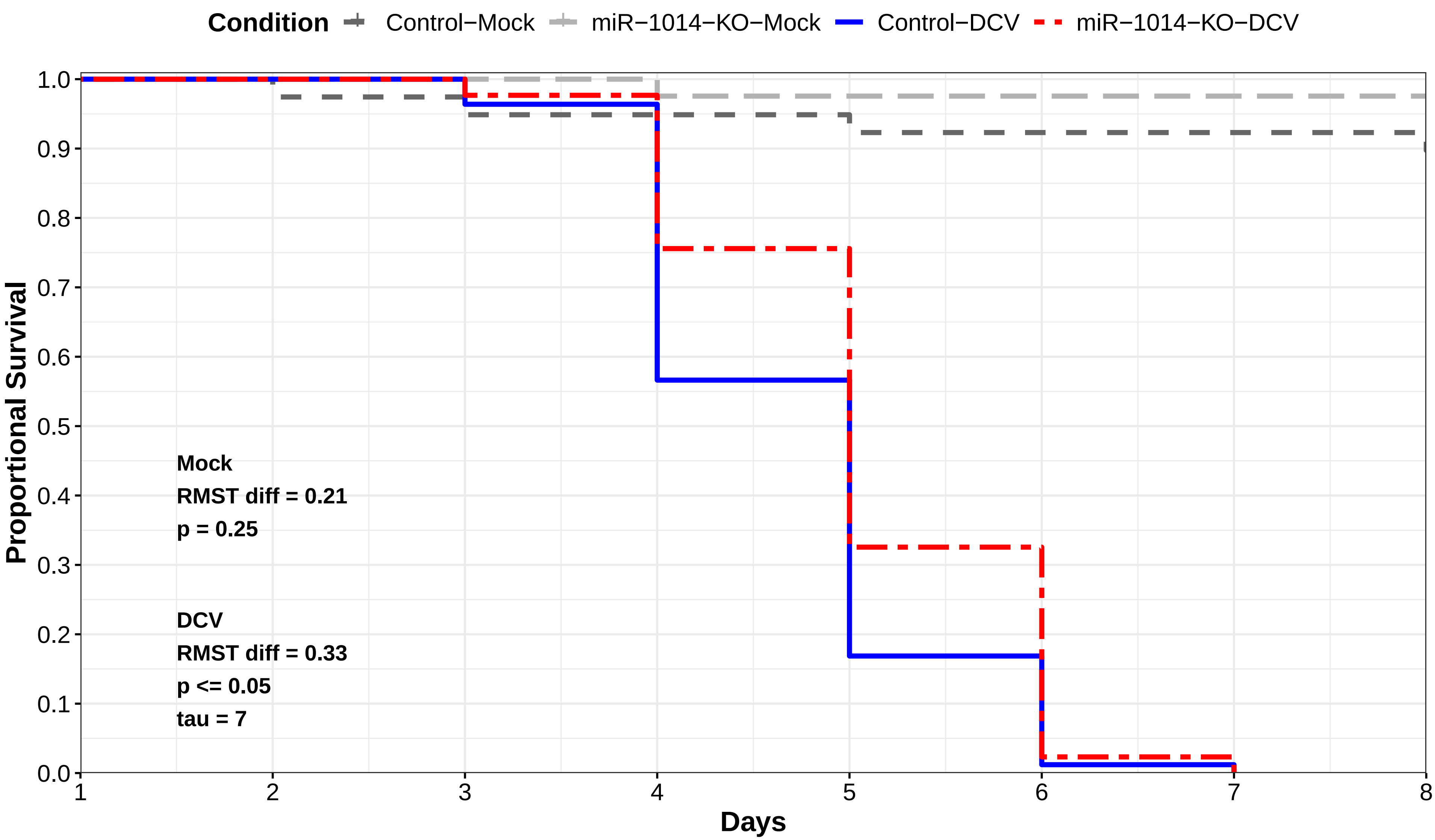
Condition
Control−Mock
miR−1014−KO−Mock
Control−DCV
miR−1014−KO−DCV
Proportional Survival
1.0
0.9
0.8
0.7
0.6
0.5
0.4
0.3
0.2
0.1
0.0
Mock
RMST diff = 0.21
p = 0.25
DCV
RMST diff = 0.33
p <= 0.05
tau = 7
1
2
3
4
5
6
7
8
Days

Figure S1: The impact of miRNA dysregulation on the outcome of DCV infection. A panel of 34 miRNA loss of function mutants were independently injected with DCV (10^7 IU/mL). Survival was monitored daily for each mutant (red, dash-dot) and compared to a paired control (blue, solid). Mock-injected mortality is also included for each line: mutant (light grey, long-dash) and control (dark grey, short-dash). Mortality occurring within the first 24 h was considered needle-stick injury and censored. Statistical analyses were conducted using Restricted Mean Survival Time (RMST) in R (v4.5.1). In this context, RMST difference is the approximate difference in survival between groups. A negative RMST difference can be interpreted as days decrease in survival, while a positive RMST difference as days increase in survival versus control (e.g. miR-31b has a RMST difference of 5.3, thus on average flies of the miR-31b loss of function line lived 5.3 days longer than control). Truncation time (tau) for both analyses was identified as the higher of the maximum survival times between DCV-injected mutant and control. A p-value < 0.05 indicates a statistically significant difference between the mutant line and control. For the DCV-injected populations, biological replicate was included as a covariate. Three independent biological replicates were performed for each line. Presented survival curves are the composite of all replicates.
