# Supplementary material for: Dysregulation of miRNAs has broad impacts on virus infection in Drosophila
Source: J Virol. 2026 Jul 2;100(7):e00850-26. doi: 10.1128/jvi.00850-26 (PMC13386962; doi:10.1128/jvi.00850-26)
Supplement: Fig. S2 — FHV survival curves. [file jvi.00850-26-s0004.pdf]

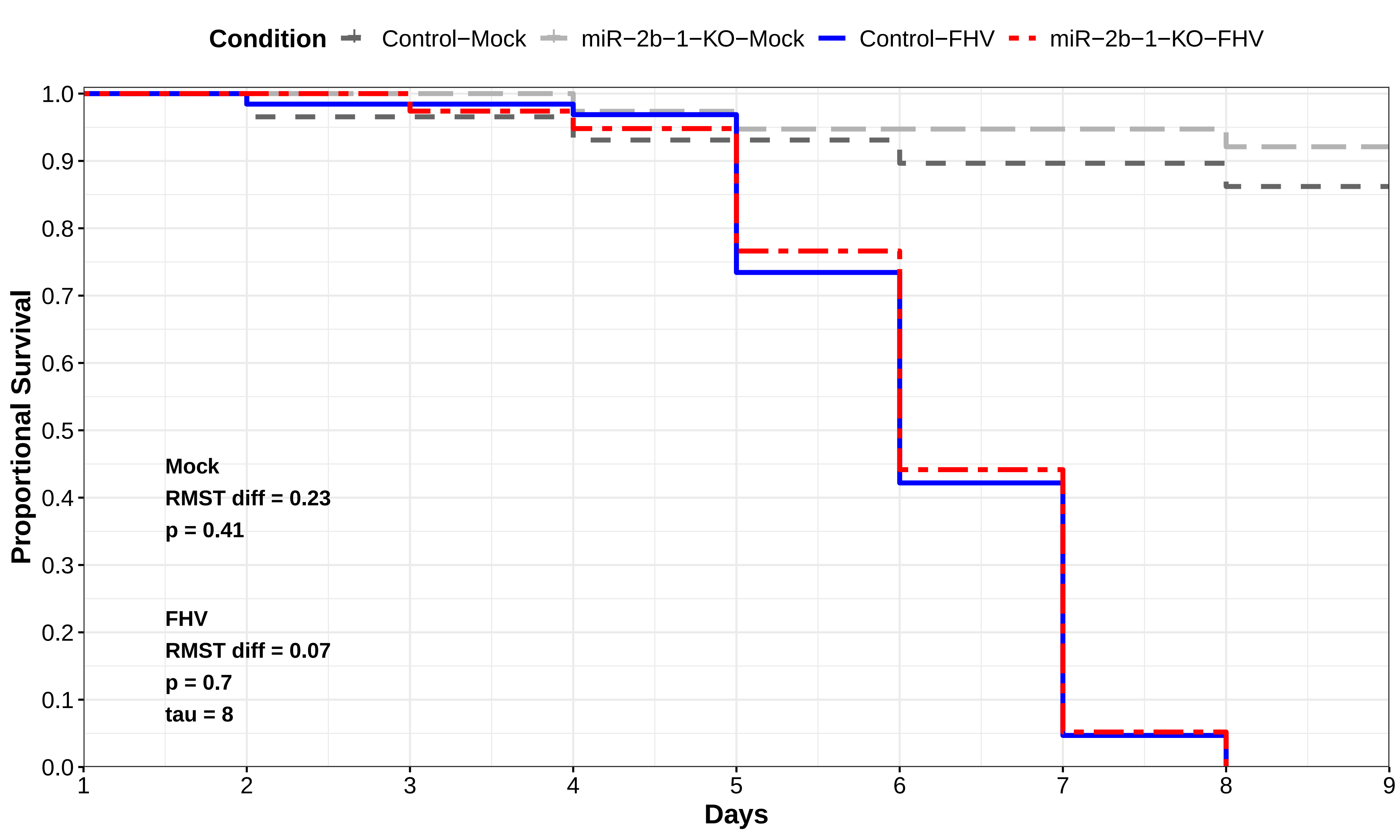
Condition
Control−Mock
miR−2b−1−KO−Mock
Control−FHV
miR−2b−1−KO−FHV
Proportional Survival
1.0
0.9
0.8
0.7
0.6
0.5
0.4
0.3
0.2
0.1
0.0
Mock
RMST diff = 0.23
p = 0.41
FHV
RMST diff = 0.07
p = 0.7
tau = 8
1
2
3
4
5
6
7
8
9
Days

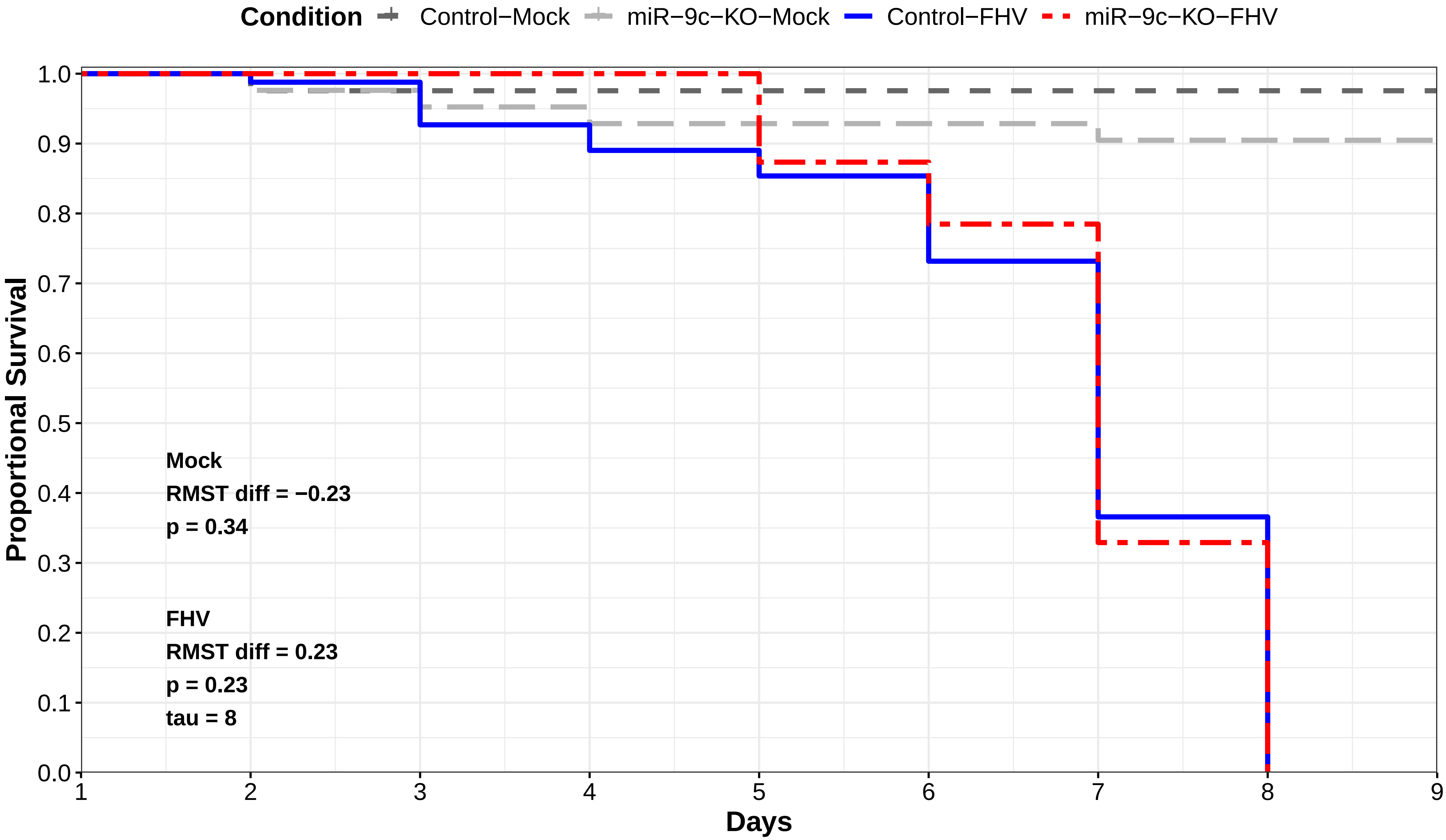
Condition
Control−Mock
miR−9c−KO−Mock
Control−FHV
miR−9c−KO−FHV
1.0
0.9
0.8
0.7
0.6
0.5
0.4
0.3
0.2
0.1
0.0
Proportional Survival
Mock
RMST diff = −0.23
p = 0.34
FHV
RMST diff = 0.23
p = 0.23
tau = 8
1
2
3
4
5
6
7
8
9
Days

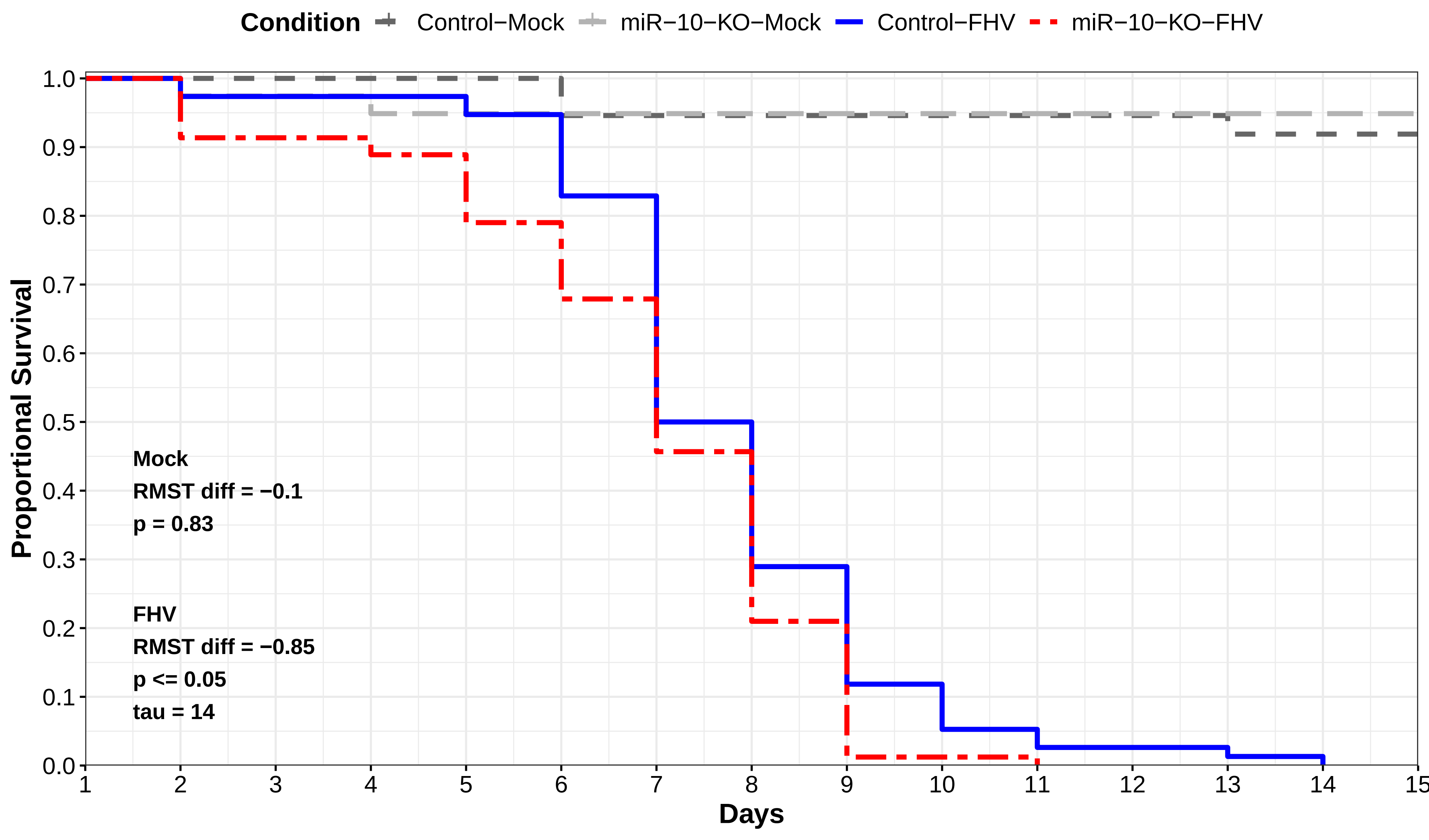

Condition
Control−Mock
miR−10−KO−Mock
Control−FHV
miR−10−KO−FHV
Proportional Survival
1.0
0.9
0.8
0.7
0.6
0.5
0.4
0.3
0.2
0.1
0.0
Mock
RMST diff = −0.1
p = 0.83
FHV
RMST diff = −0.85
p <= 0.05
tau = 14
1
2
3
4
5
6
7
8
9
10
11
12
13
14
15
Days

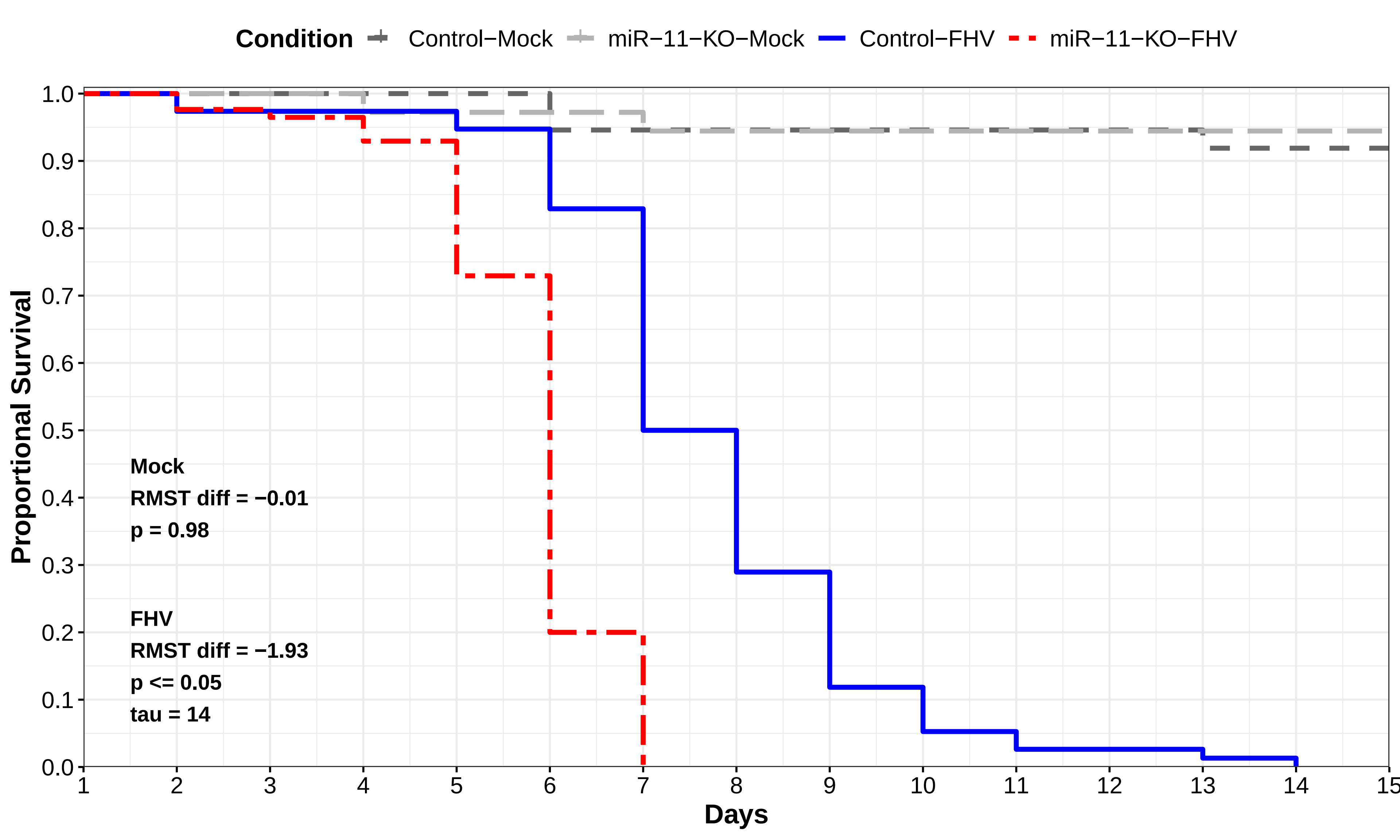

Condition
Control−Mock
miR−11−KO−Mock
Control−FHV
miR−11−KO−FHV
Proportional Survival
1.0
0.9
0.8
0.7
0.6
0.5
0.4
0.3
0.2
0.1
0.0
Mock
RMST diff = −0.01
p = 0.98
FHV
RMST diff = −1.93
p <= 0.05
tau = 14
1
2
3
4
5
6
7
8
9
10
11
12
13
14
15
Days

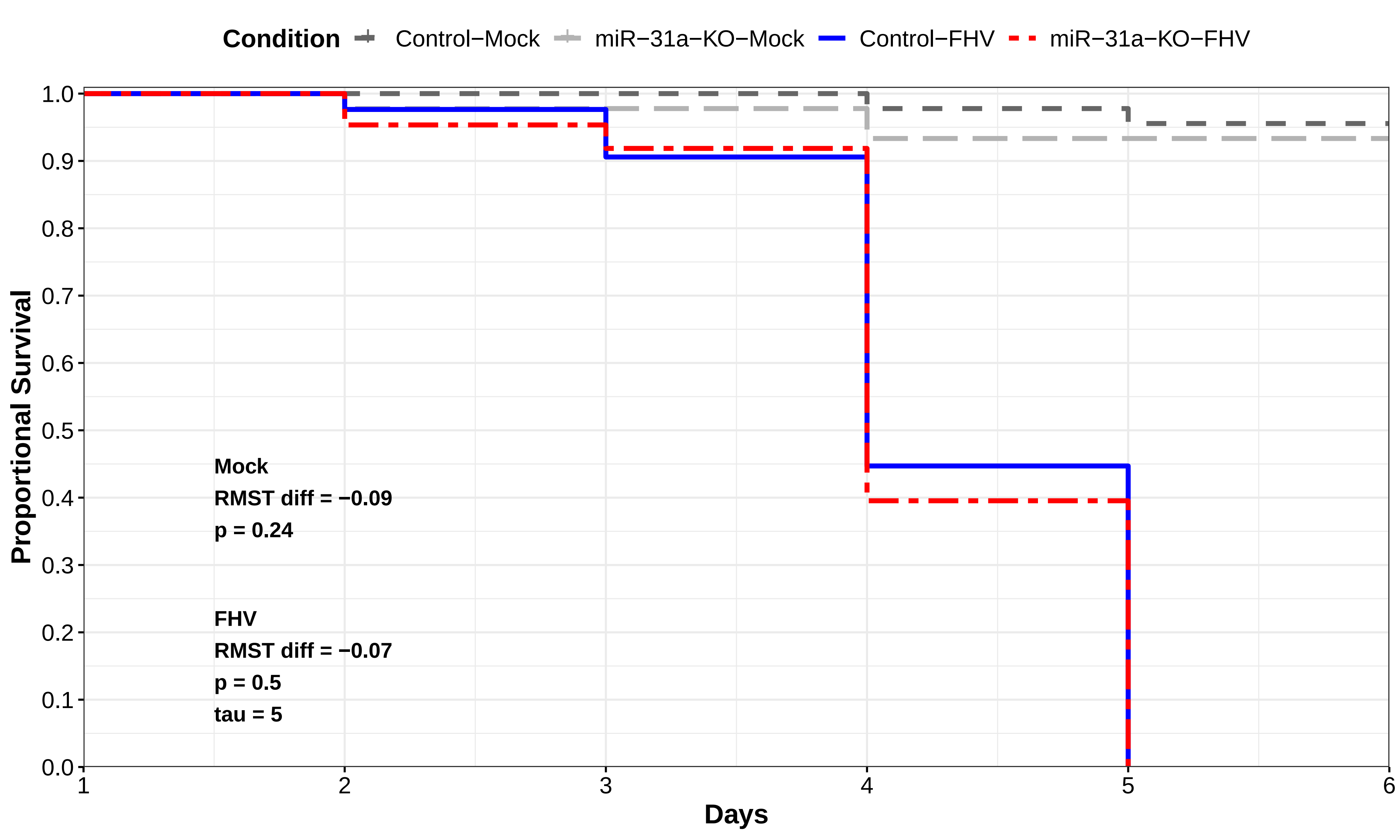

Condition
Control−Mock
miR−31a−KO−Mock
Control−FHV
miR−31a−KO−FHV
Proportional Survival
1.0
0.9
0.8
0.7
0.6
0.5
0.4
0.3
0.2
0.1
0.0
Mock
RMST diff = −0.09
p = 0.24
FHV
RMST diff = −0.07
p = 0.5
tau = 5
1
2
3
4
5
6
Days

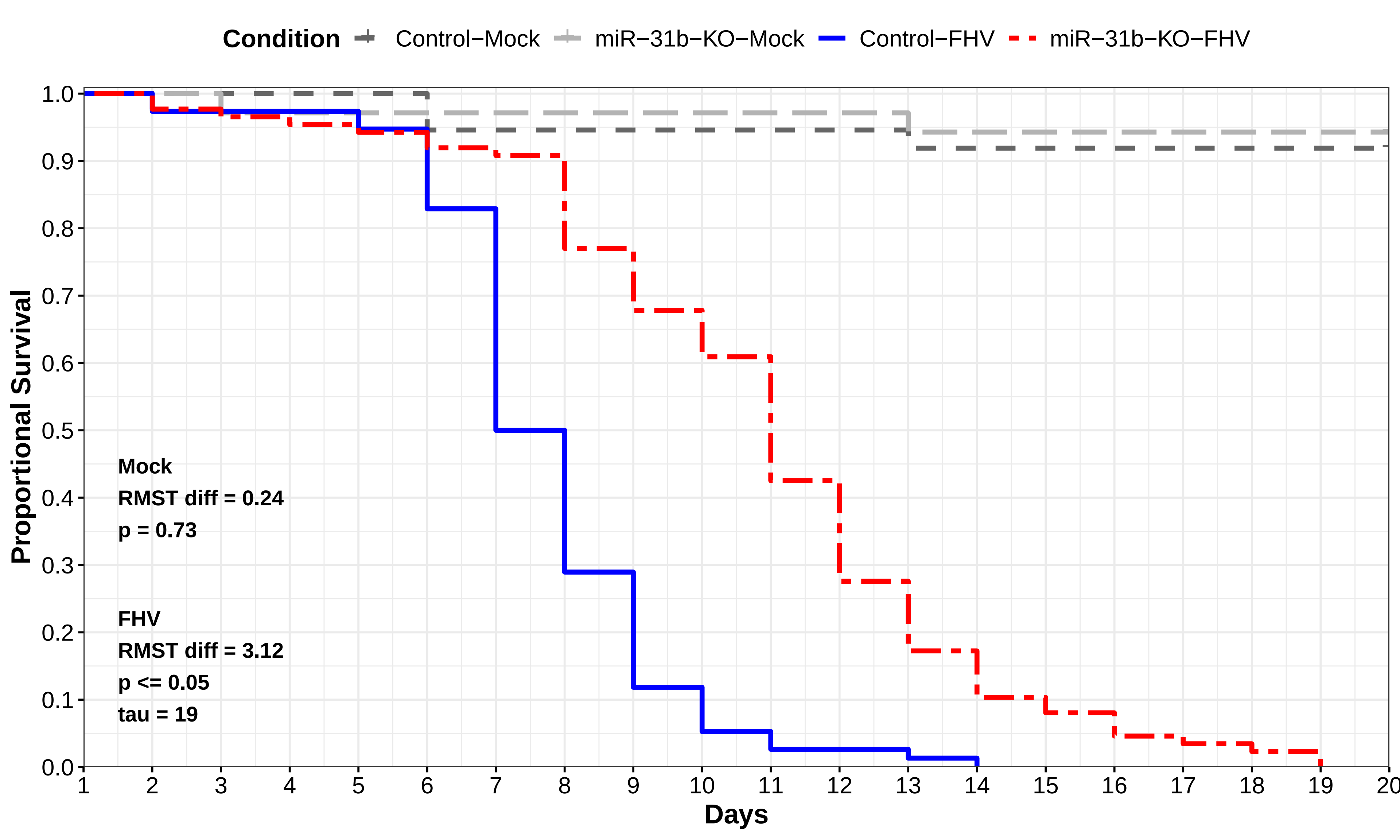

Condition
Control−Mock
miR−31b−KO−Mock
Control−FHV
miR−31b−KO−FHV
Proportional Survival
1.0
0.9
0.8
0.7
0.6
0.5
0.4
0.3
0.2
0.1
0.0
Mock
RMST diff = 0.24
p = 0.73
FHV
RMST diff = 3.12
p <= 0.05
tau = 19
1
2
3
4
5
6
7
8
9
10
11
12
13
14
15
16
17
18
19
20
Days

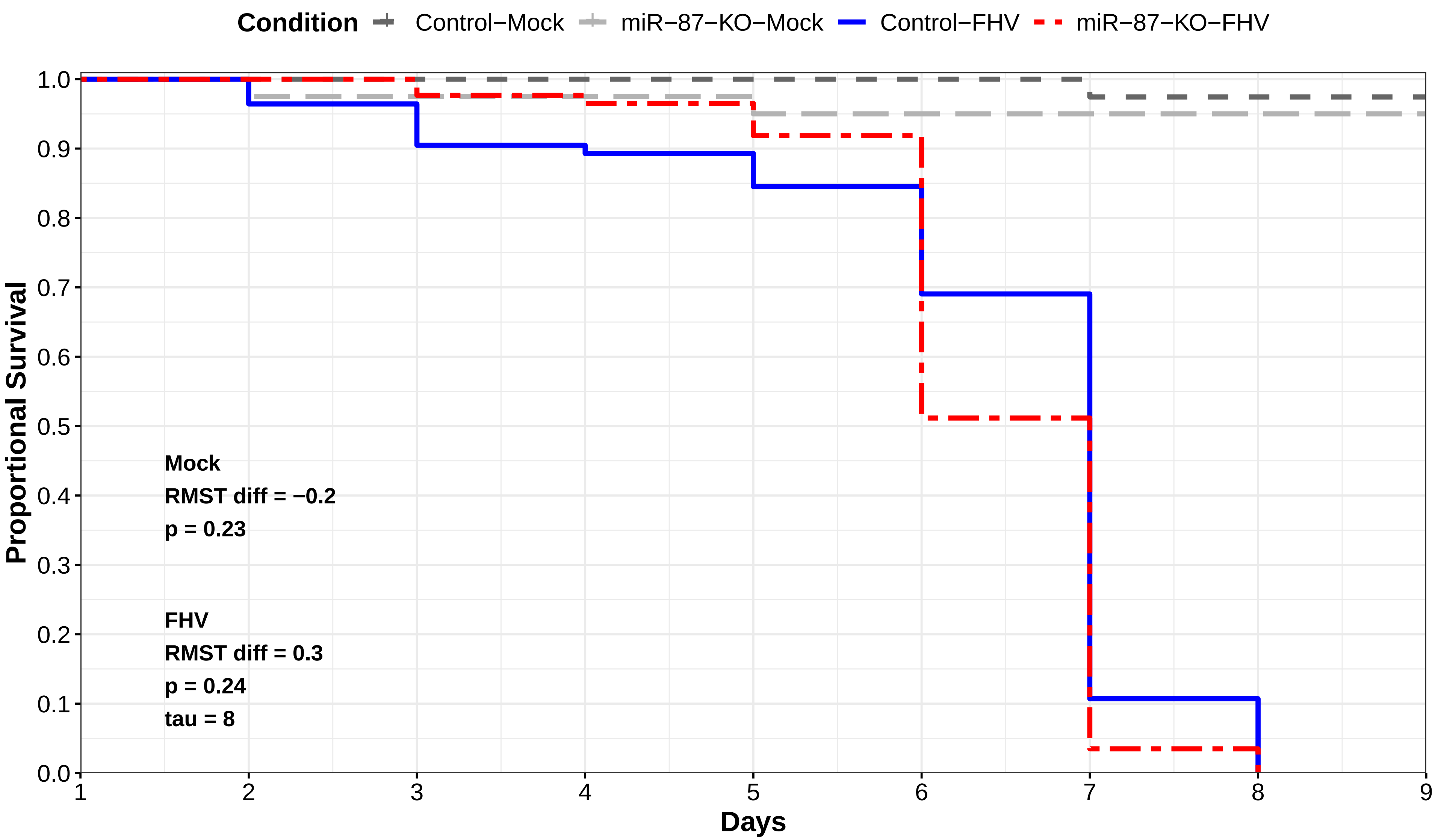

Condition
Control−Mock
miR−87−KO−Mock
Control−FHV
miR−87−KO−FHV
Proportional Survival
1.0
0.9
0.8
0.7
0.6
0.5
0.4
0.3
0.2
0.1
0.0
Mock
RMST diff = −0.2
p = 0.23
FHV
RMST diff = 0.3
p = 0.24
tau = 8
1
2
3
4
5
6
7
8
9
Days

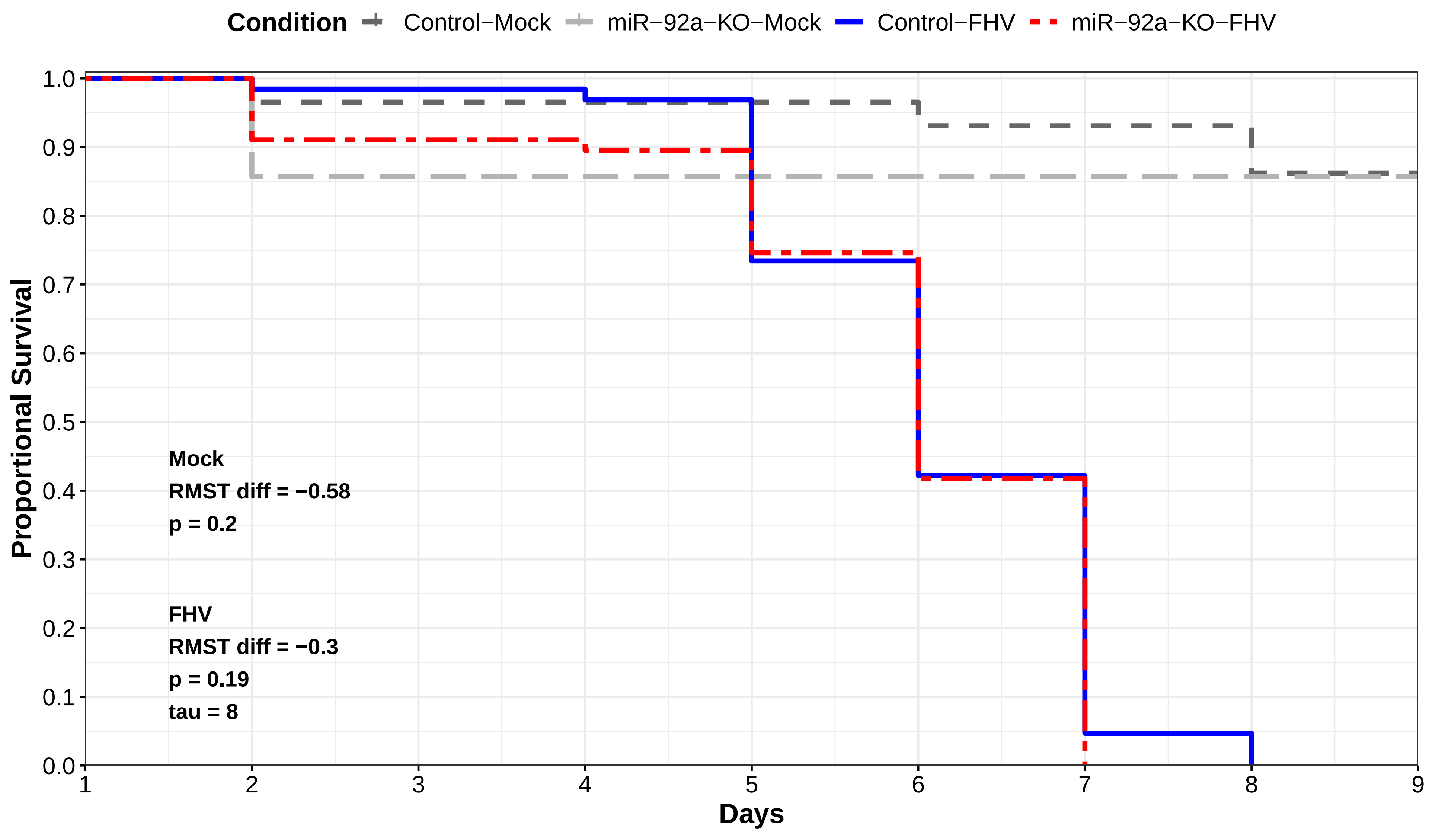

Condition
Control−Mock
miR−92a−KO−Mock
Control−FHV
miR−92a−KO−FHV
Proportional Survival
1.0
0.9
0.8
0.7
0.6
0.5
0.4
0.3
0.2
0.1
0.0
Mock
RMST diff = −0.58
p = 0.2
FHV
RMST diff = −0.3
p = 0.19
tau = 8
1
2
3
4
5
6
7
8
9
Days

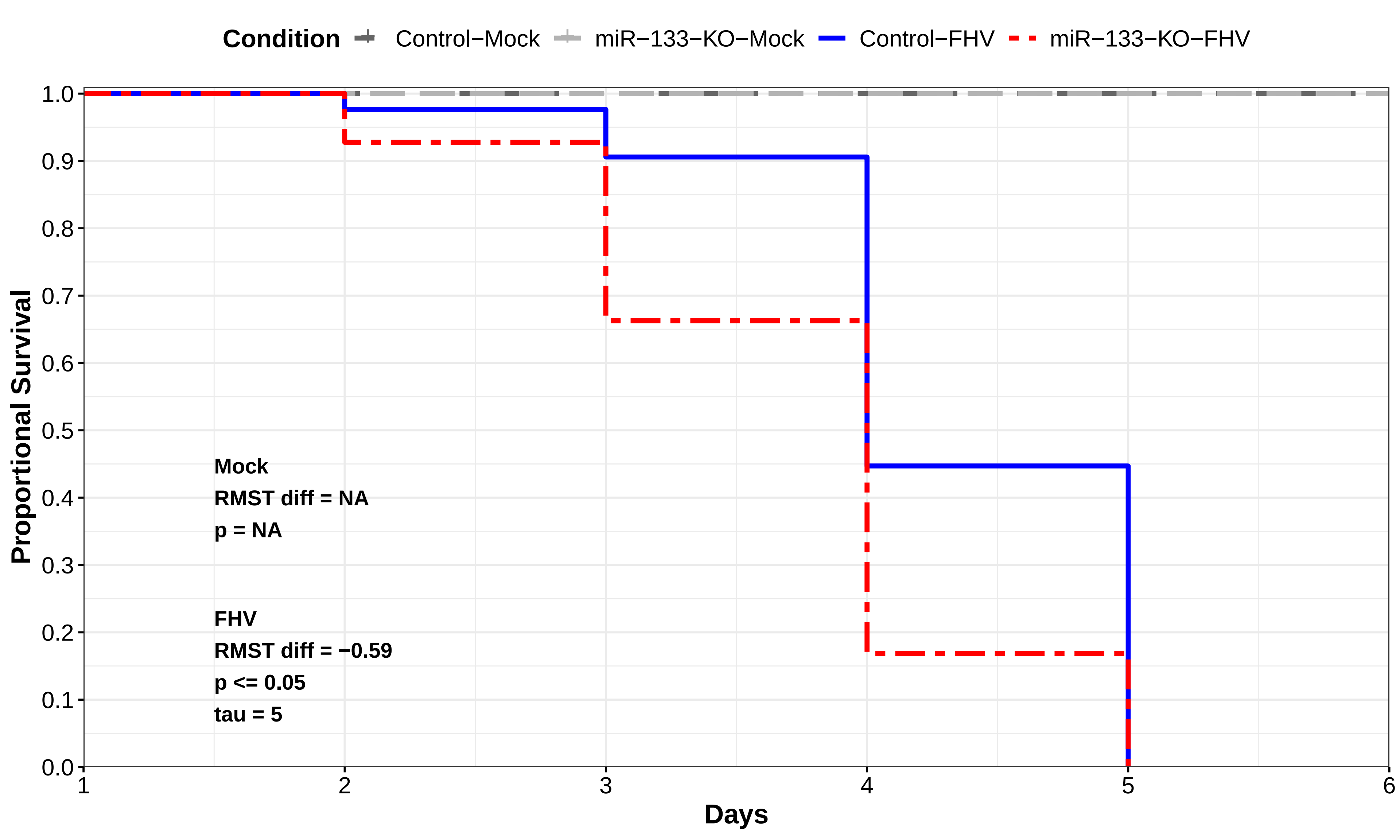

Condition
Control−Mock
miR−133−KO−Mock
Control−FHV
miR−133−KO−FHV
Proportional Survival
1.0
0.9
0.8
0.7
0.6
0.5
0.4
0.3
0.2
0.1
0.0
1
2
3
4
5
6
Days
Mock
RMST diff = NA
p = NA
FHV
RMST diff = −0.59
p <= 0.05
tau = 5

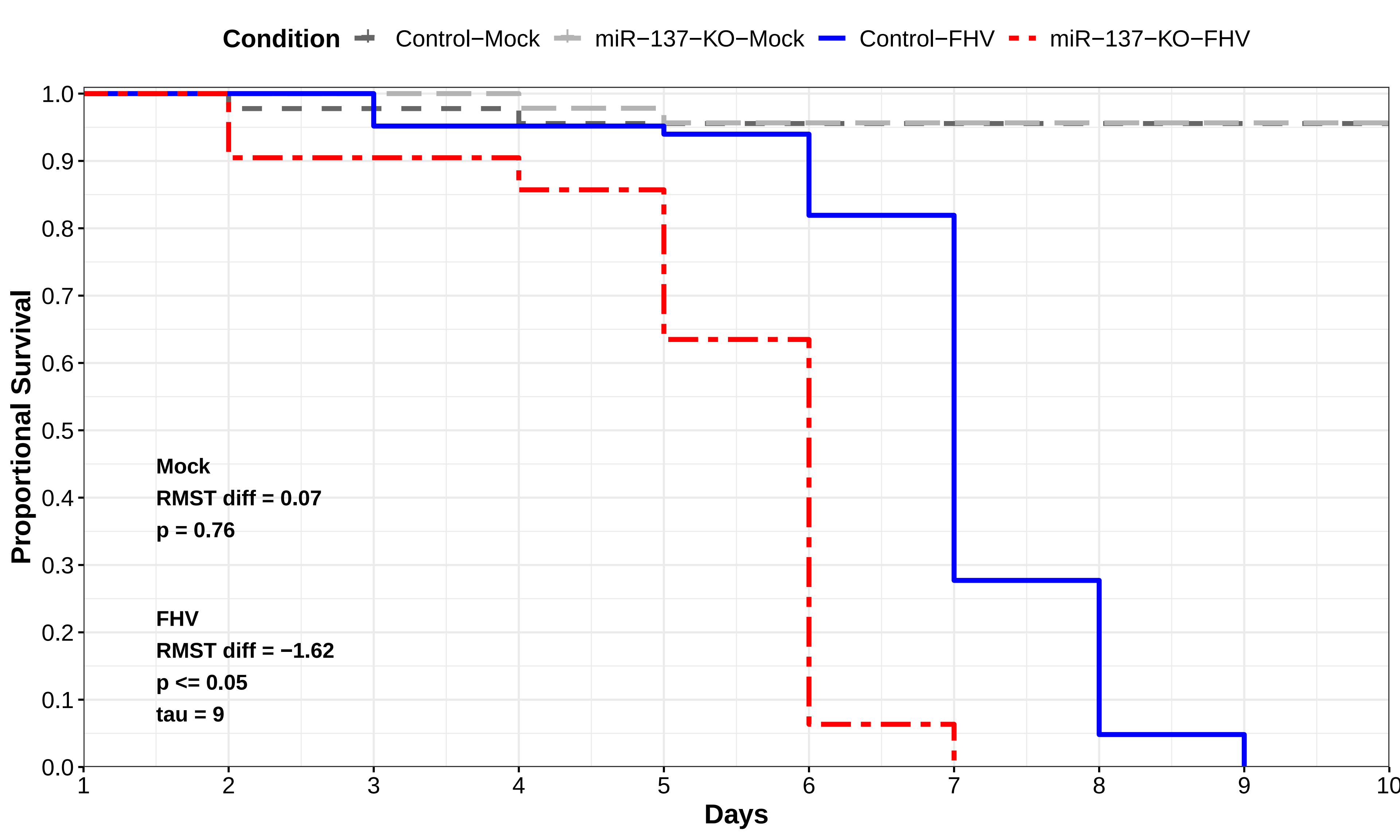
Condition
Control−Mock
miR−137−KO−Mock
Control−FHV
miR−137−KO−FHV
Proportional Survival
1.0
0.9
0.8
0.7
0.6
0.5
0.4
0.3
0.2
0.1
0.0
1
2
3
4
5
6
7
8
9
10
Days
Mock
RMST diff = 0.07
p = 0.76
FHV
RMST diff = −1.62
p <= 0.05
tau = 9

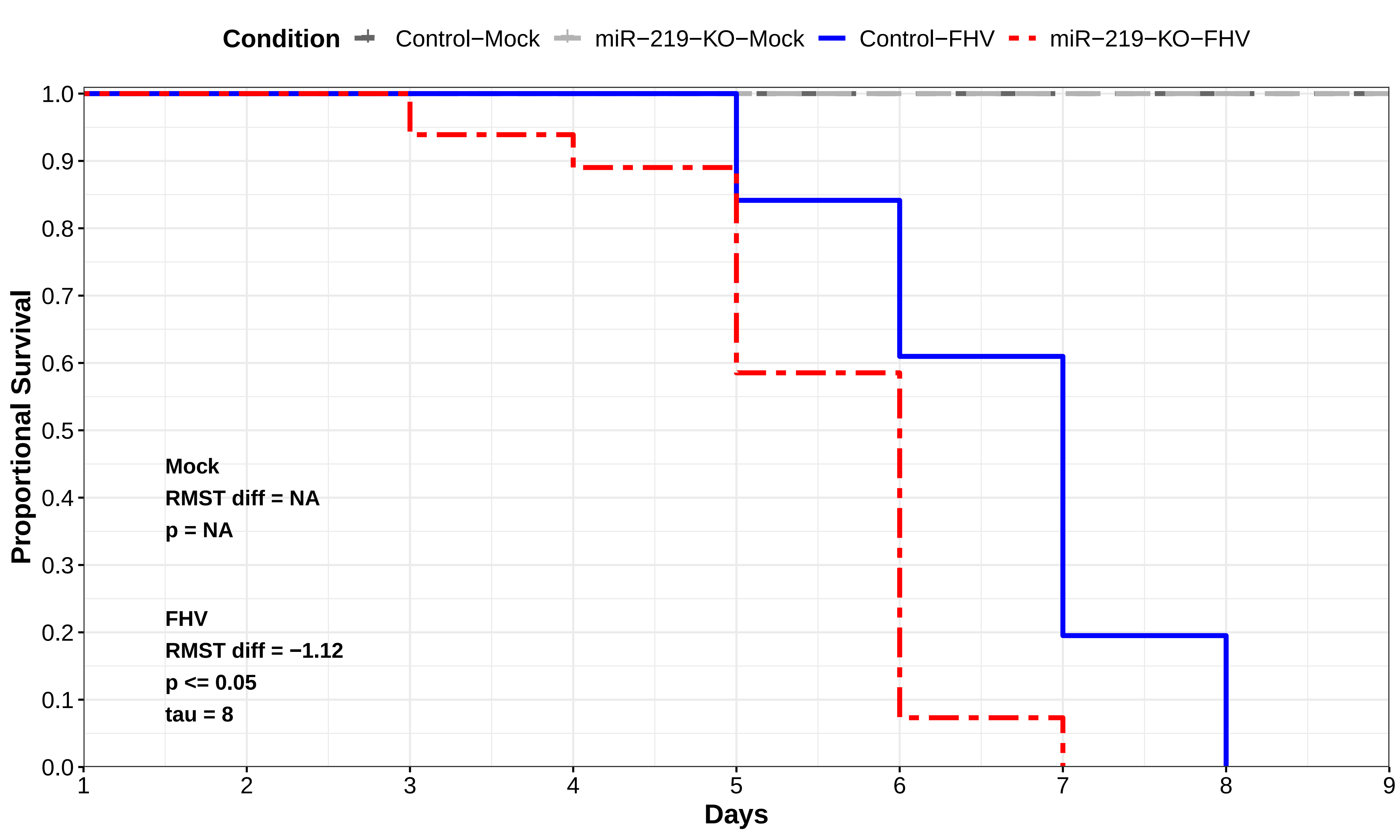

Condition
Control−Mock
miR−219−KO−Mock
Control−FHV
miR−219−KO−FHV
Proportional Survival
1.0
0.9
0.8
0.7
0.6
0.5
0.4
0.3
0.2
0.1
0.0
Mock
RMST diff = NA
p = NA
FHV
RMST diff = −1.12
p <= 0.05
tau = 8
1
2
3
4
5
6
7
8
9
Days

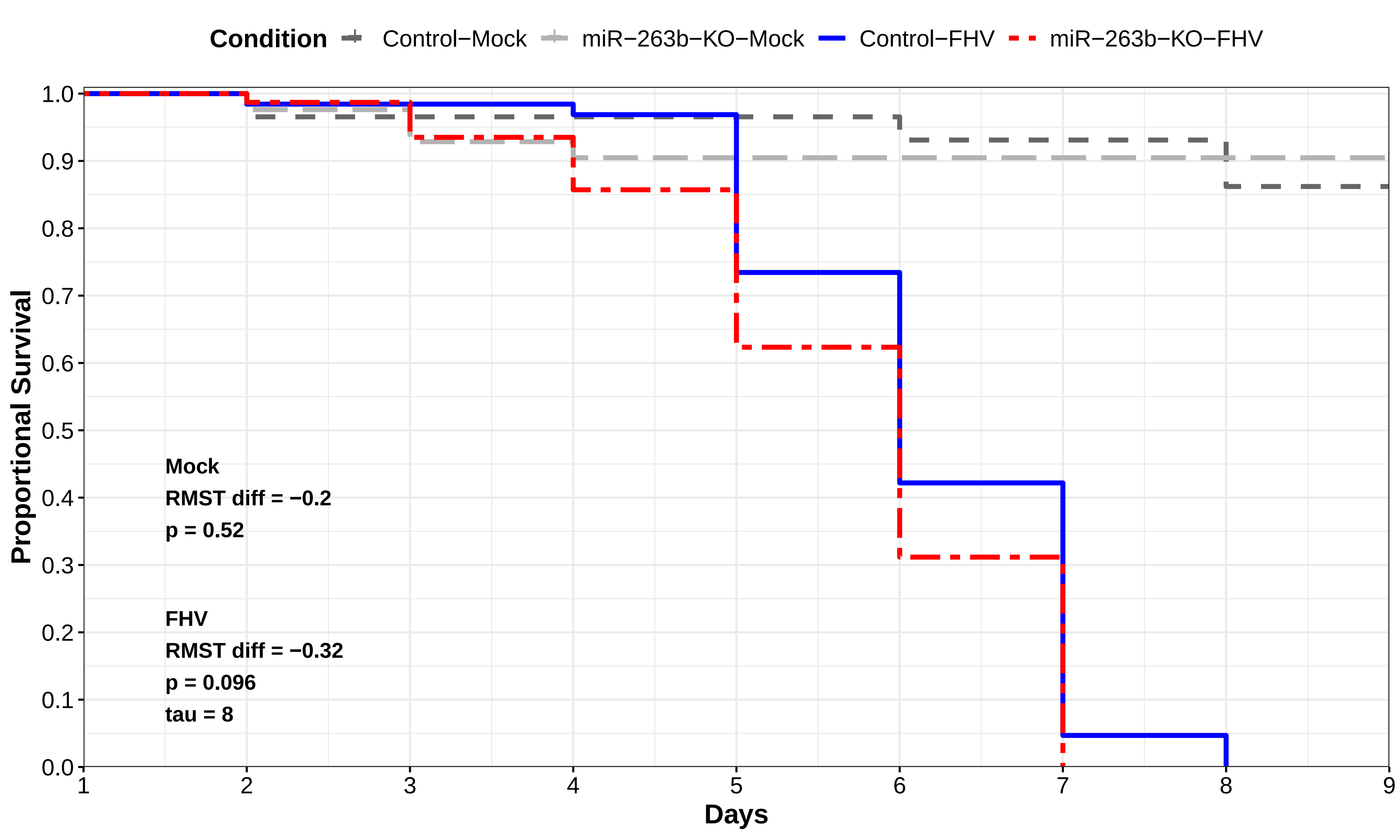

Condition
Control−Mock
miR−263b−KO−Mock
Control−FHV
miR−263b−KO−FHV
Proportional Survival
1.0
0.9
0.8
0.7
0.6
0.5
0.4
0.3
0.2
0.1
0.0
Mock
RMST diff = −0.2
p = 0.52
FHV
RMST diff = −0.32
p = 0.096
tau = 8
1
2
3
4
5
6
7
8
9
Days

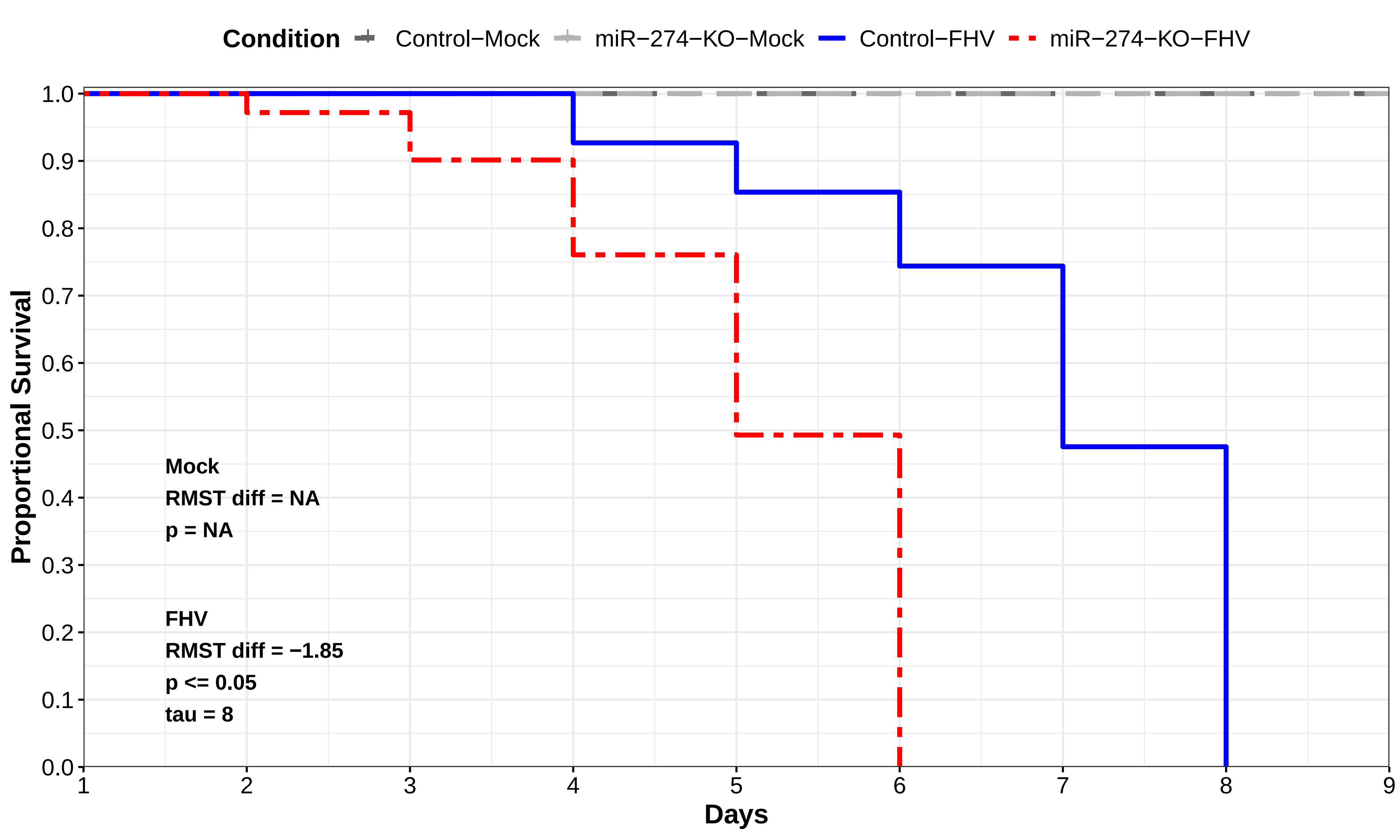
Condition
Control−Mock
miR−274−KO−Mock
Control−FHV
miR−274−KO−FHV
Proportional Survival
1.0
0.9
0.8
0.7
0.6
0.5
0.4
0.3
0.2
0.1
0.0
Mock
RMST diff = NA
p = NA
FHV
RMST diff = −1.85
p <= 0.05
tau = 8
1
2
3
4
5
6
7
8
9
Days

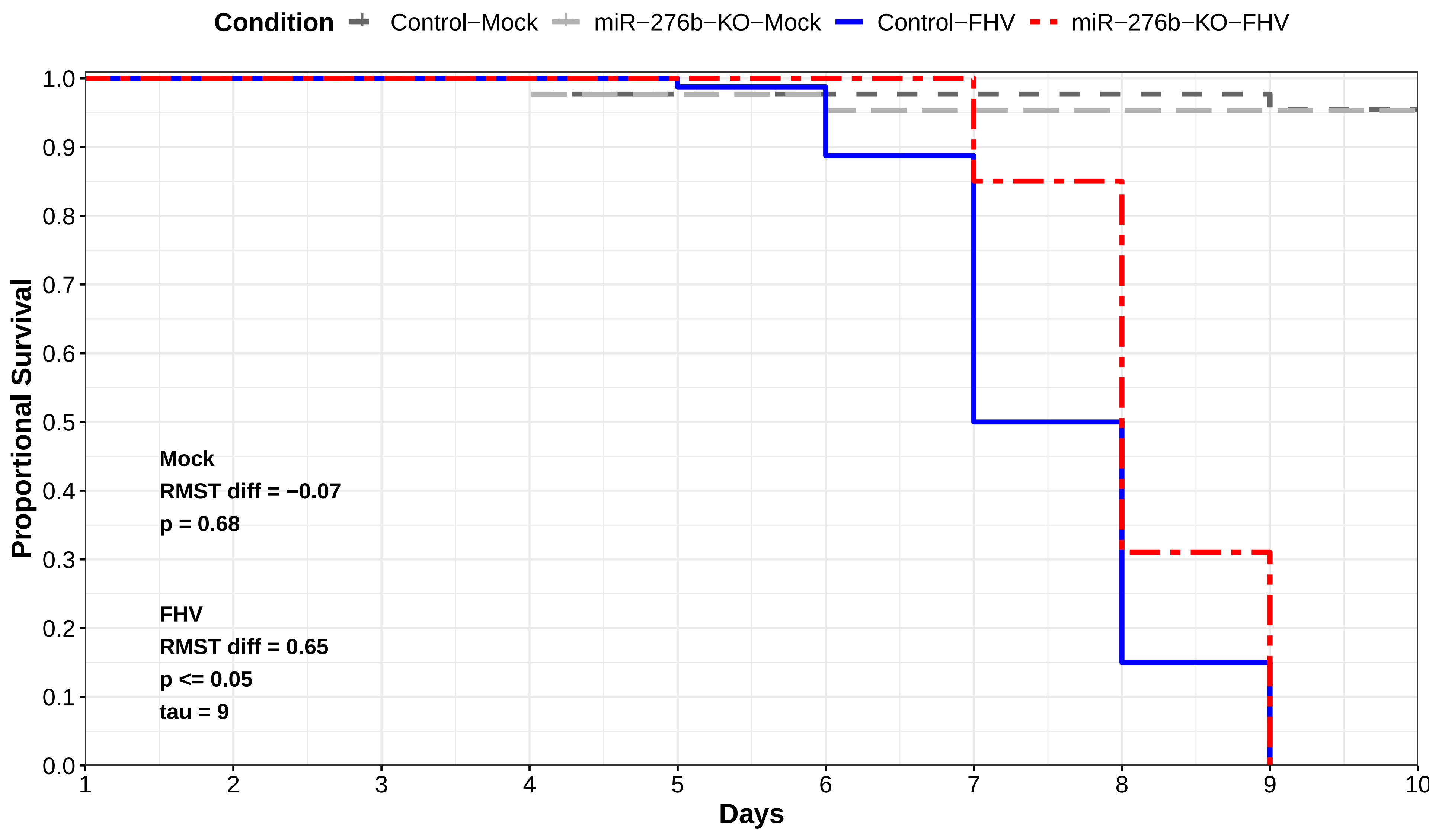

Condition
Control−Mock
miR−276b−KO−Mock
Control−FHV
miR−276b−KO−FHV
Proportional Survival
1.0
0.9
0.8
0.7
0.6
0.5
0.4
0.3
0.2
0.1
0.0
Mock
RMST diff = −0.07
p = 0.68
FHV
RMST diff = 0.65
p <= 0.05
tau = 9
1
2
3
4
5
6
7
8
9
10
Days

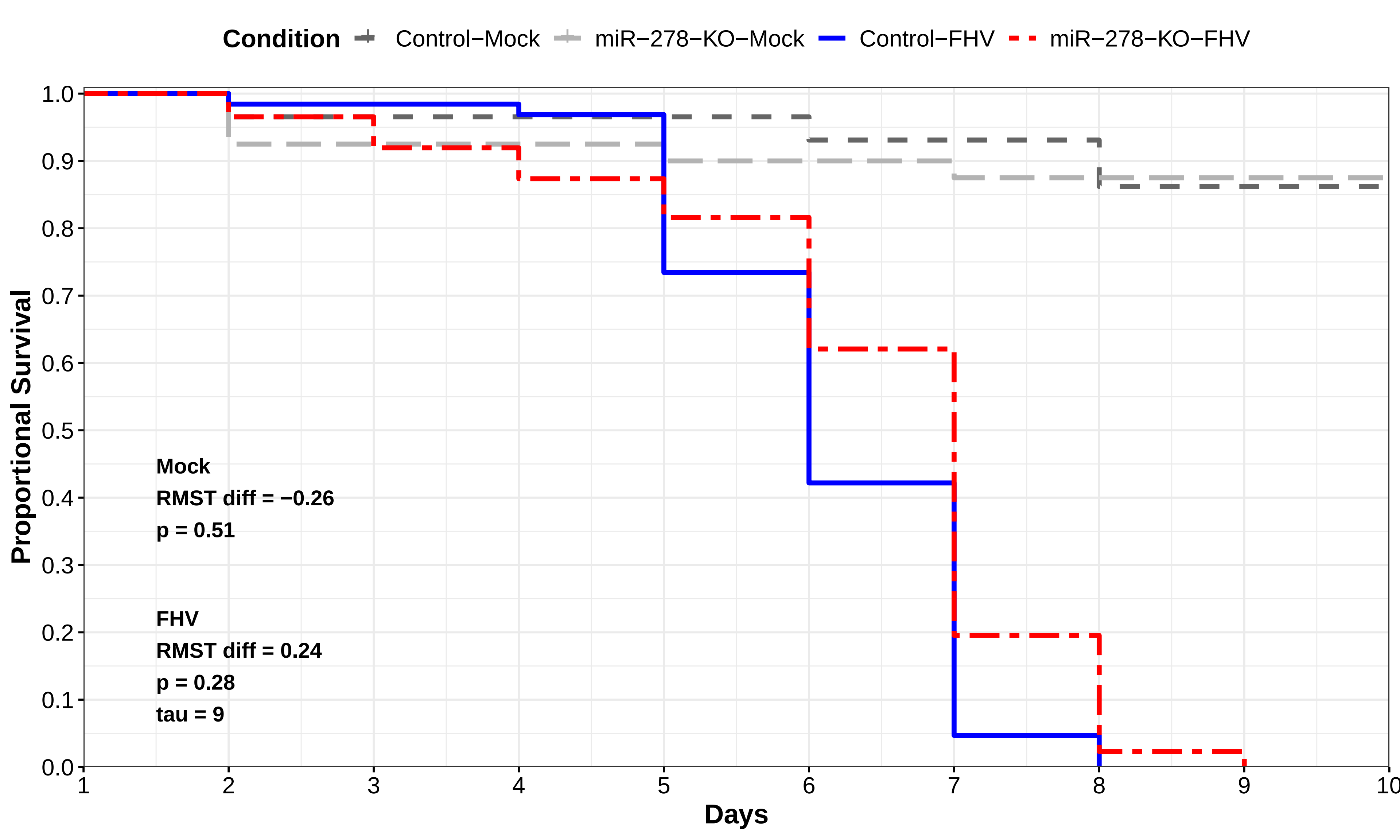
Condition
Control−Mock
miR−278−KO−Mock
Control−FHV
miR−278−KO−FHV
Proportional Survival
Days
Mock
RMST diff = −0.26
p = 0.51
FHV
RMST diff = 0.24
p = 0.28
tau = 9

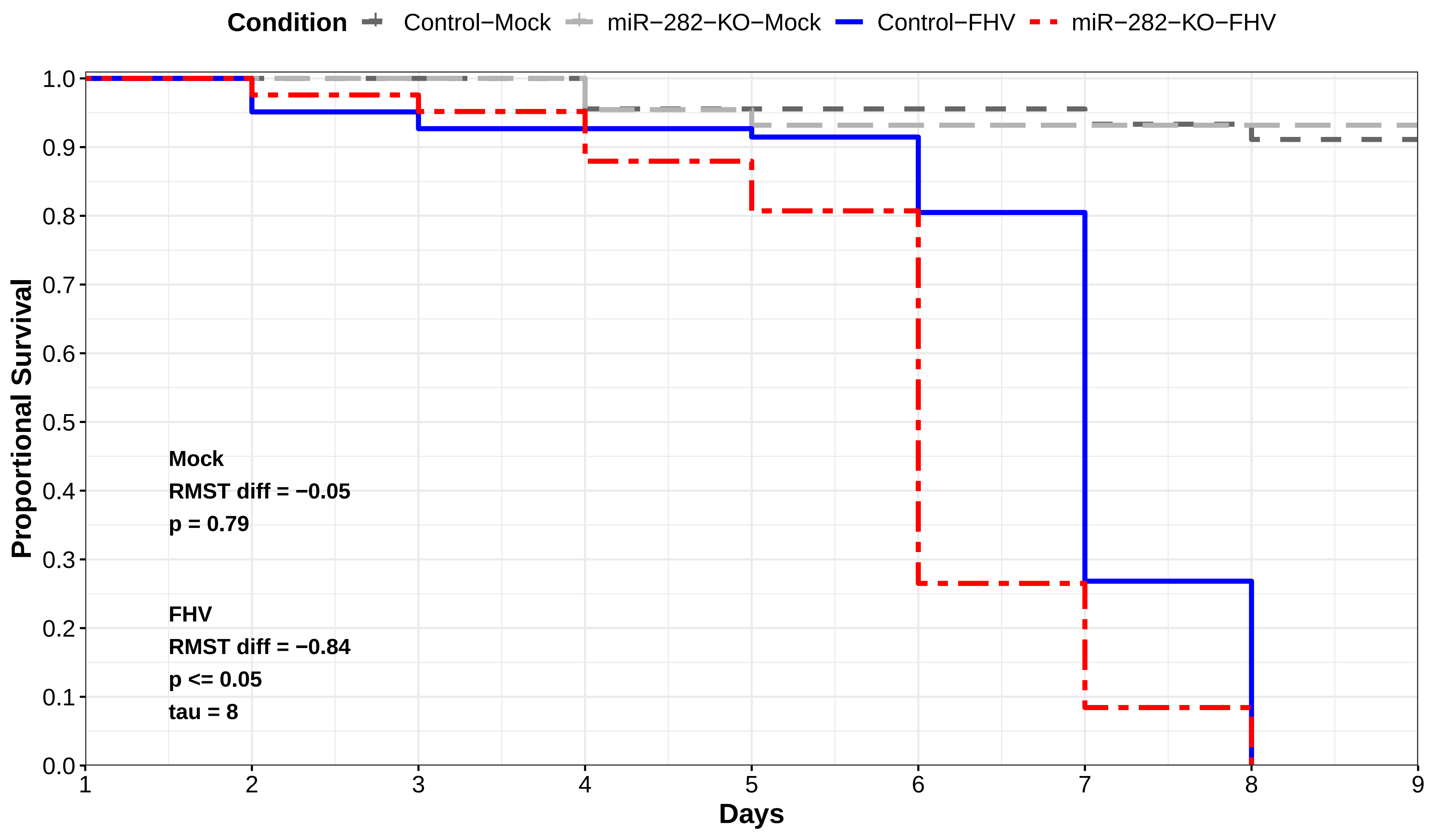

Condition
Control−Mock
miR−282−KO−Mock
Control−FHV
miR−282−KO−FHV
Proportional Survival
1.0
0.9
0.8
0.7
0.6
0.5
0.4
0.3
0.2
0.1
0.0
Mock
RMST diff = −0.05
p = 0.79
FHV
RMST diff = −0.84
p <= 0.05
tau = 8
1
2
3
4
5
6
7
8
9
Days

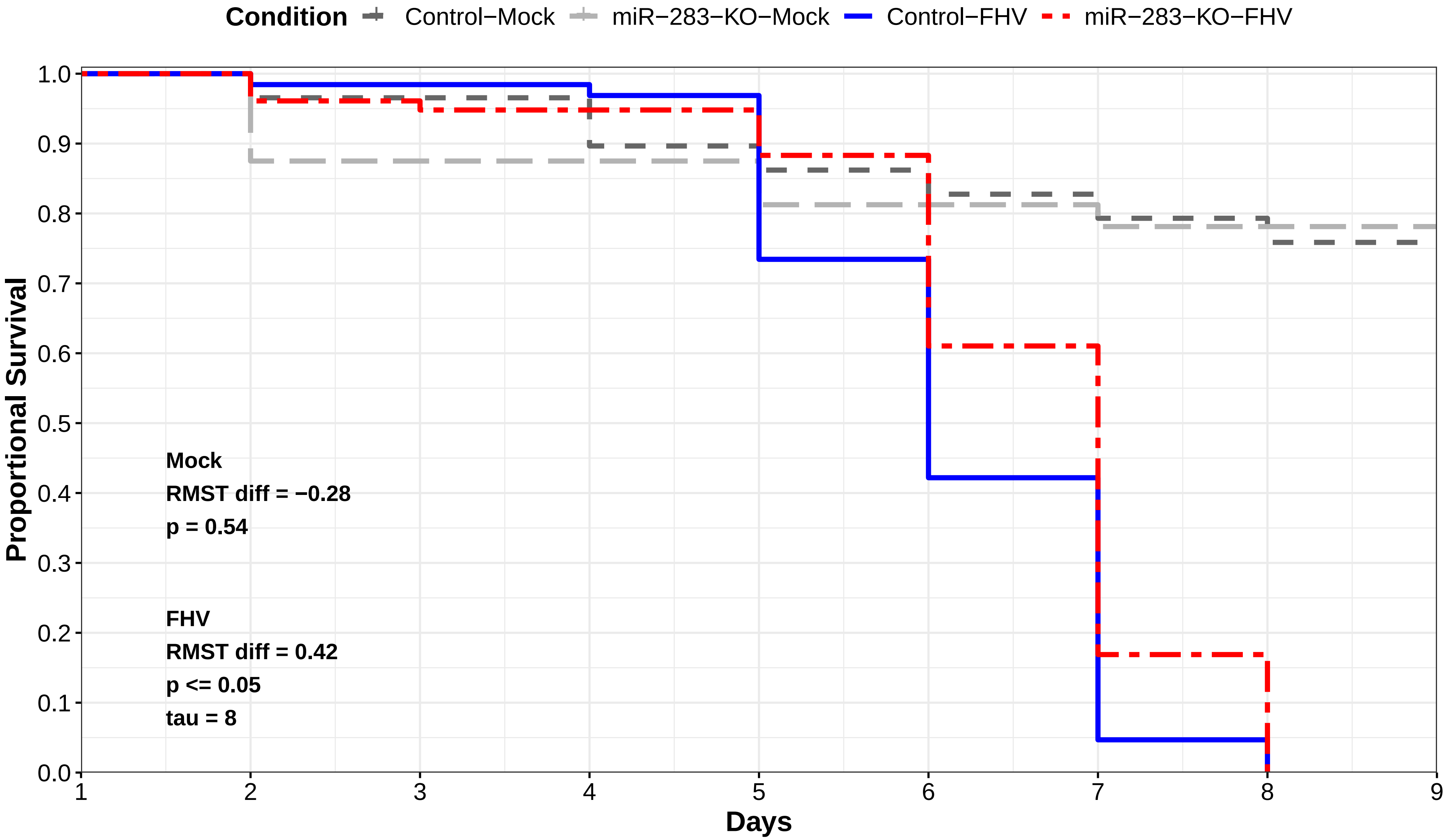

Condition
Control−Mock
miR−283−KO−Mock
Control−FHV
miR−283−KO−FHV
Proportional Survival
1.0
0.9
0.8
0.7
0.6
0.5
0.4
0.3
0.2
0.1
0.0
Mock
RMST diff = −0.28
p = 0.54
FHV
RMST diff = 0.42
p <= 0.05
tau = 8
1
2
3
4
5
6
7
8
9
Days

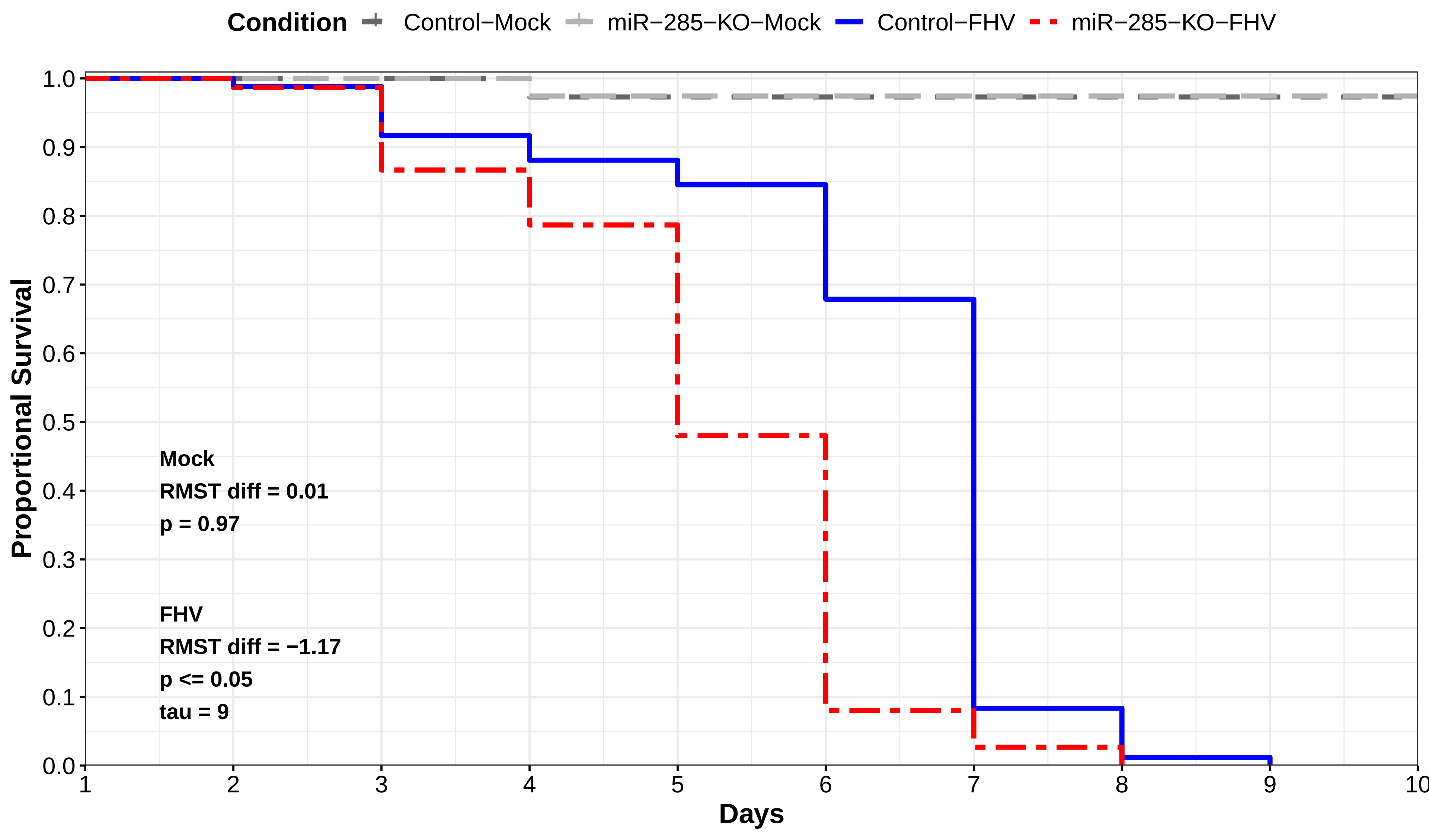
Condition
Control−Mock
miR−285−KO−Mock
Control−FHV
miR−285−KO−FHV
Proportional Survival
1.0
0.9
0.8
0.7
0.6
0.5
0.4
0.3
0.2
0.1
0.0
Mock
RMST diff = 0.01
p = 0.97
FHV
RMST diff = −1.17
p <= 0.05
tau = 9
1
2
3
4
5
6
7
8
9
10
Days

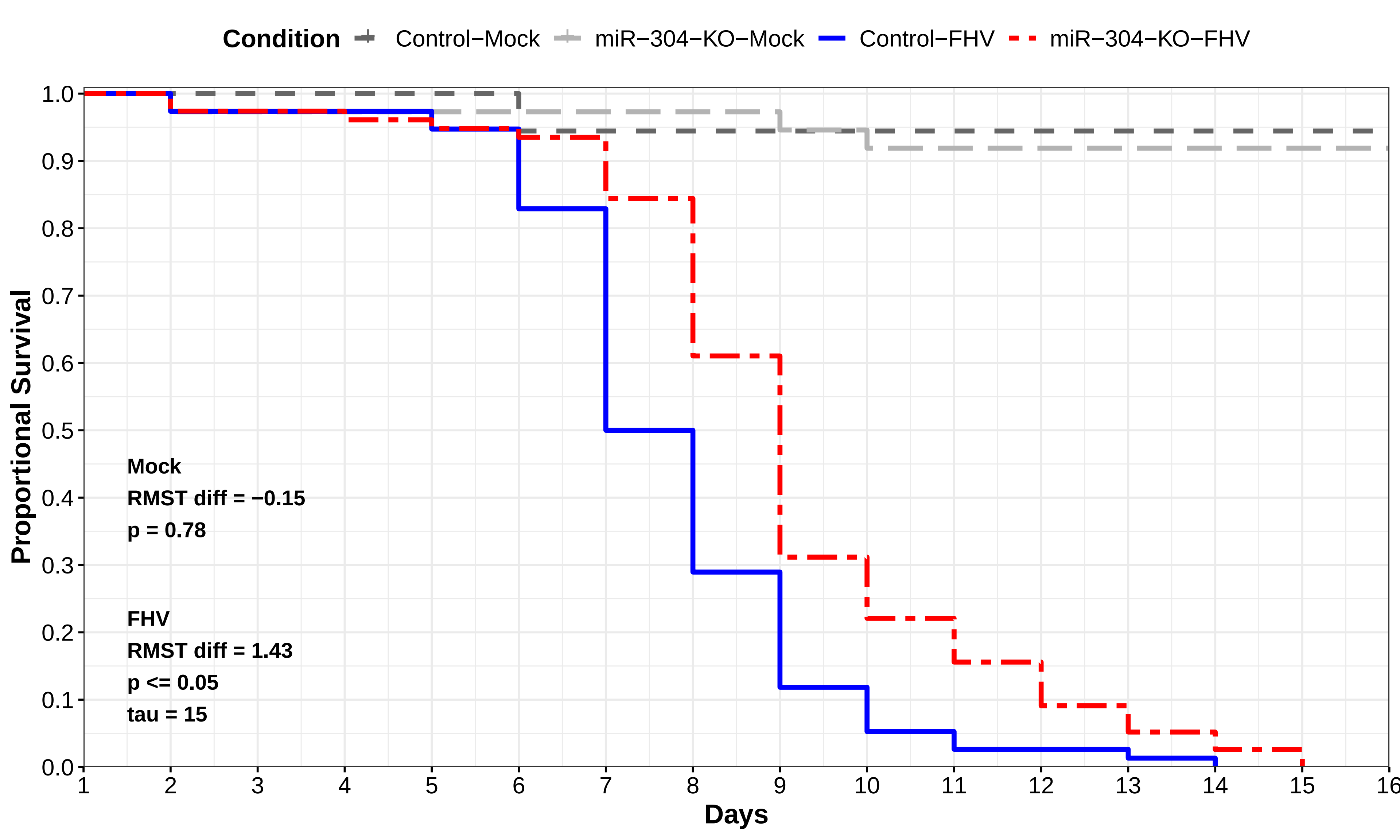

Condition
Control−Mock
miR−304−KO−Mock
Control−FHV
miR−304−KO−FHV
Proportional Survival
1.0
0.9
0.8
0.7
0.6
0.5
0.4
0.3
0.2
0.1
0.0
Mock
RMST diff = −0.15
p = 0.78
FHV
RMST diff = 1.43
p <= 0.05
tau = 15
1
2
3
4
5
6
7
8
9
10
11
12
13
14
15
16
Days

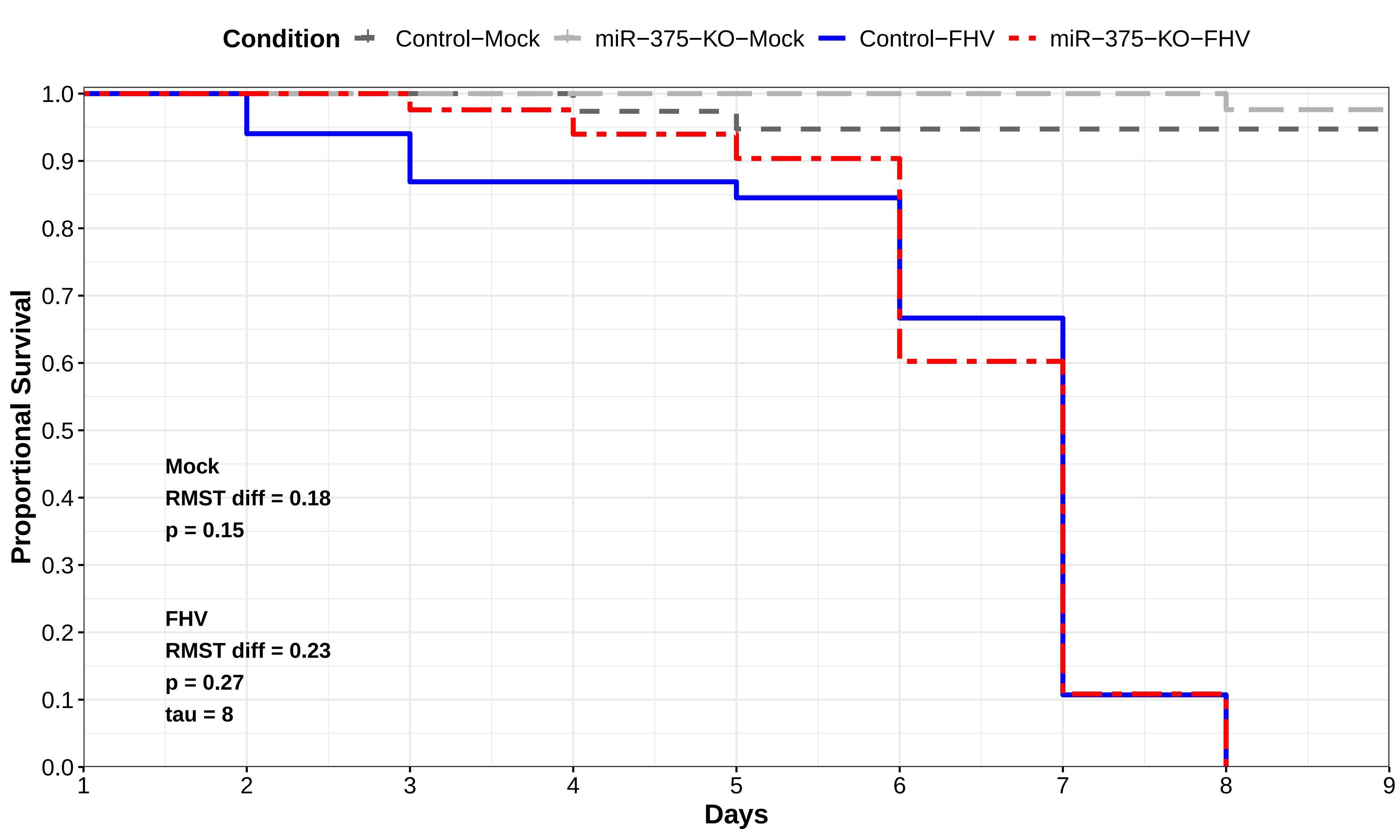

Condition
Control−Mock
miR−375−KO−Mock
Control−FHV
miR−375−KO−FHV
Proportional Survival
1.0
0.9
0.8
0.7
0.6
0.5
0.4
0.3
0.2
0.1
0.0
Mock
RMST diff = 0.18
p = 0.15
FHV
RMST diff = 0.23
p = 0.27
tau = 8
1
2
3
4
5
6
7
8
9
Days

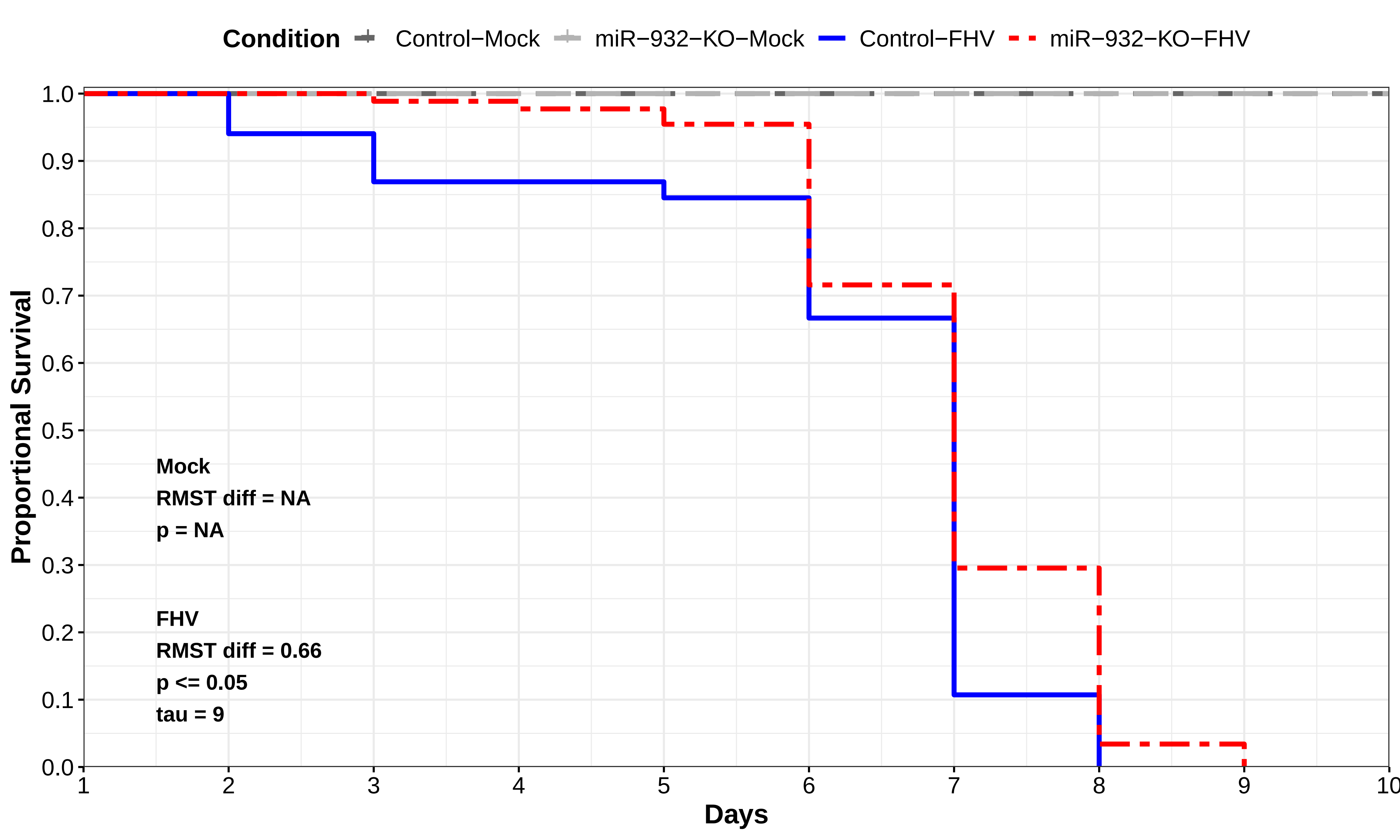
Condition
Control−Mock
miR−932−KO−Mock
Control−FHV
miR−932−KO−FHV
Proportional Survival
Days
Mock
RMST diff = NA
p = NA
FHV
RMST diff = 0.66
p <= 0.05
tau = 9
1.0
0.9
0.8
0.7
0.6
0.5
0.4
0.3
0.2
0.1
0.0
1
2
3
4
5
6
7
8
9
10

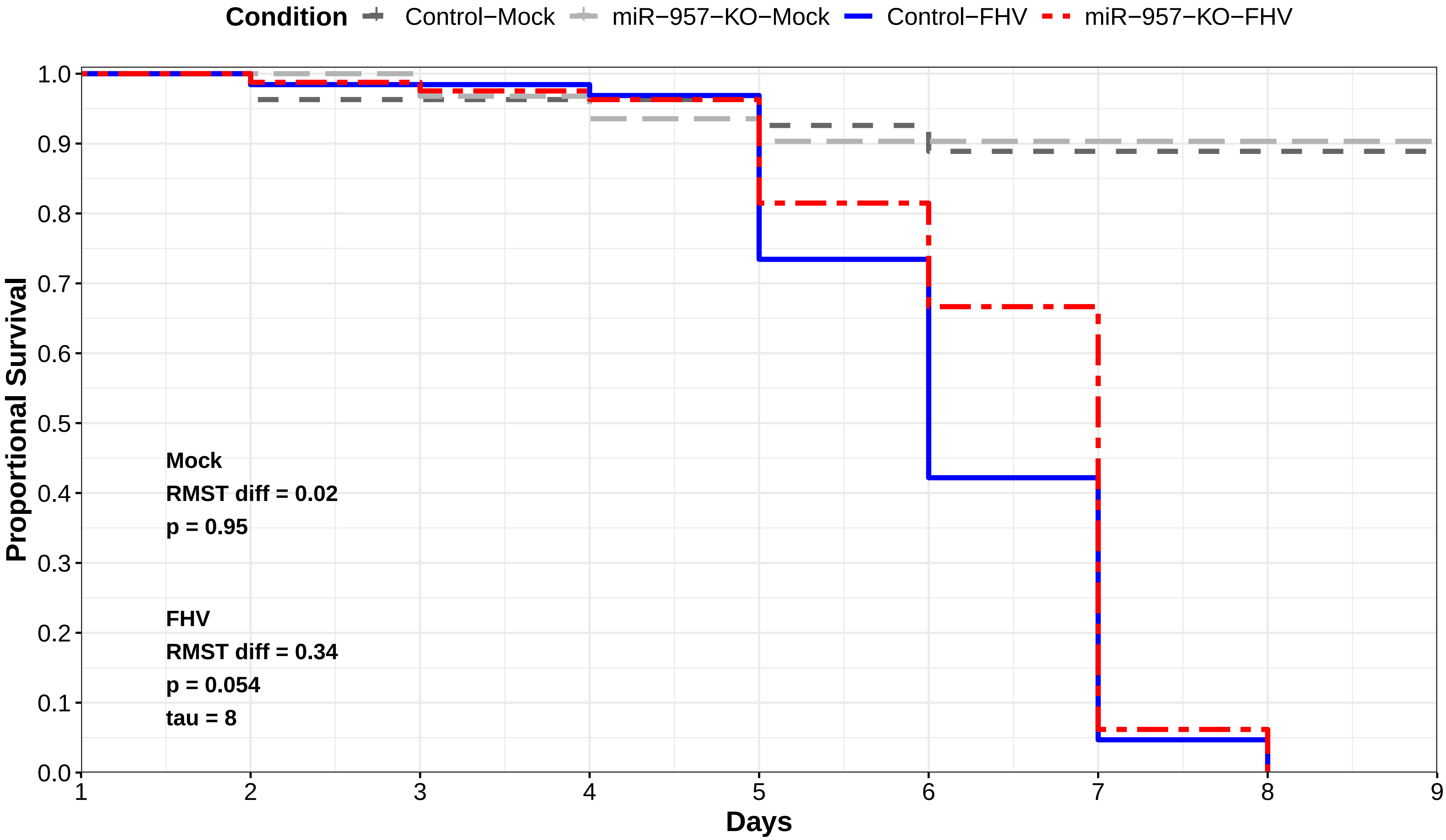

Condition
Control−Mock
miR−957−KO−Mock
Control−FHV
miR−957−KO−FHV
Proportional Survival
1.0
0.9
0.8
0.7
0.6
0.5
0.4
0.3
0.2
0.1
0.0
Mock
RMST diff = 0.02
p = 0.95
FHV
RMST diff = 0.34
p = 0.054
tau = 8
1
2
3
4
5
6
7
8
9
Days

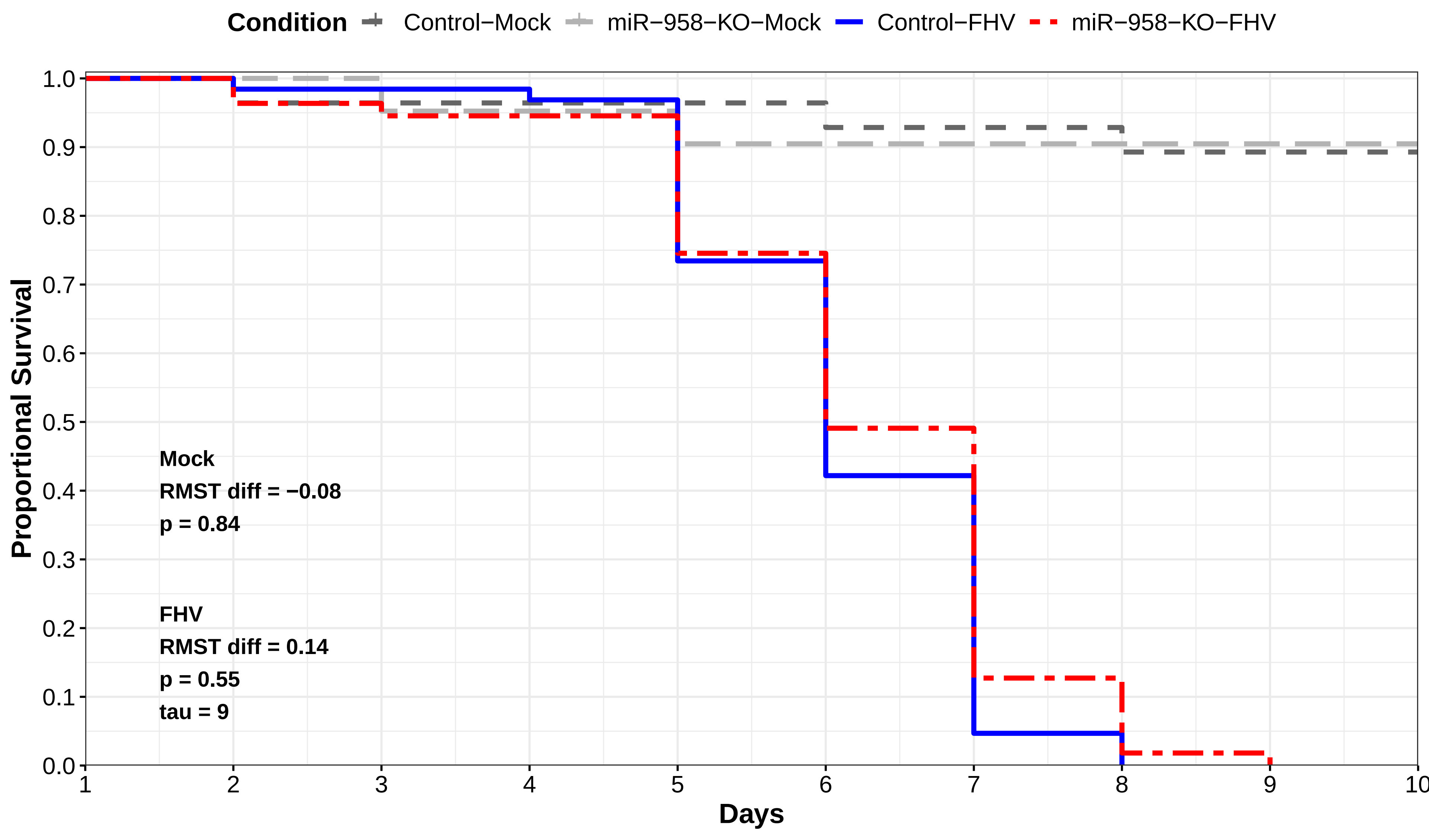

Condition
Control−Mock
miR−958−KO−Mock
Control−FHV
miR−958−KO−FHV
Proportional Survival
1.0
0.9
0.8
0.7
0.6
0.5
0.4
0.3
0.2
0.1
0.0
Mock
RMST diff = −0.08
p = 0.84
FHV
RMST diff = 0.14
p = 0.55
tau = 9
1
2
3
4
5
6
7
8
9
10
Days

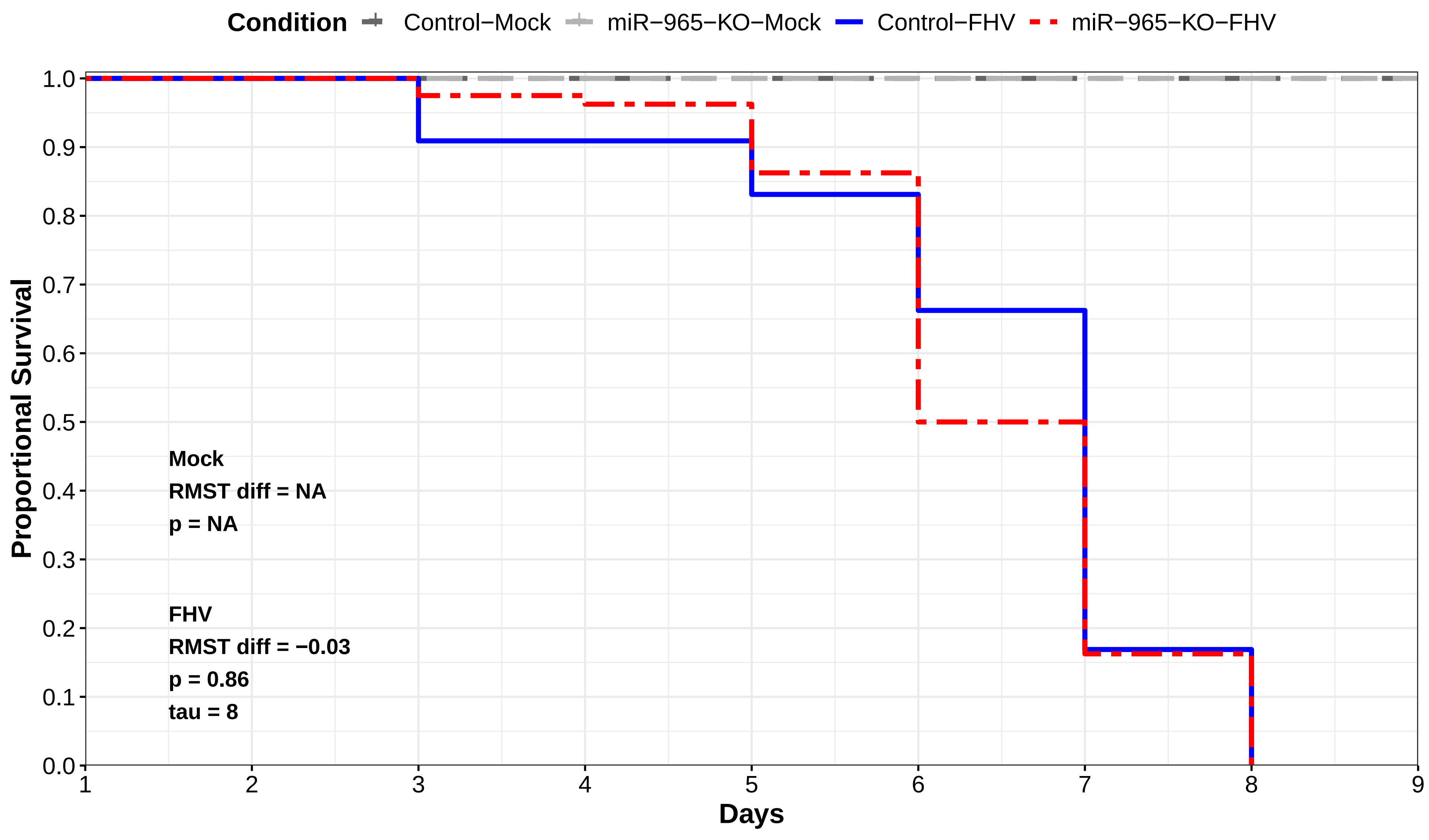
Condition
Control−Mock
miR−965−KO−Mock
Control−FHV
miR−965−KO−FHV
Proportional Survival
1.0
0.9
0.8
0.7
0.6
0.5
0.4
0.3
0.2
0.1
0.0
Mock
RMST diff = NA
p = NA
FHV
RMST diff = −0.03
p = 0.86
tau = 8
1
2
3
4
5
6
7
8
9
Days

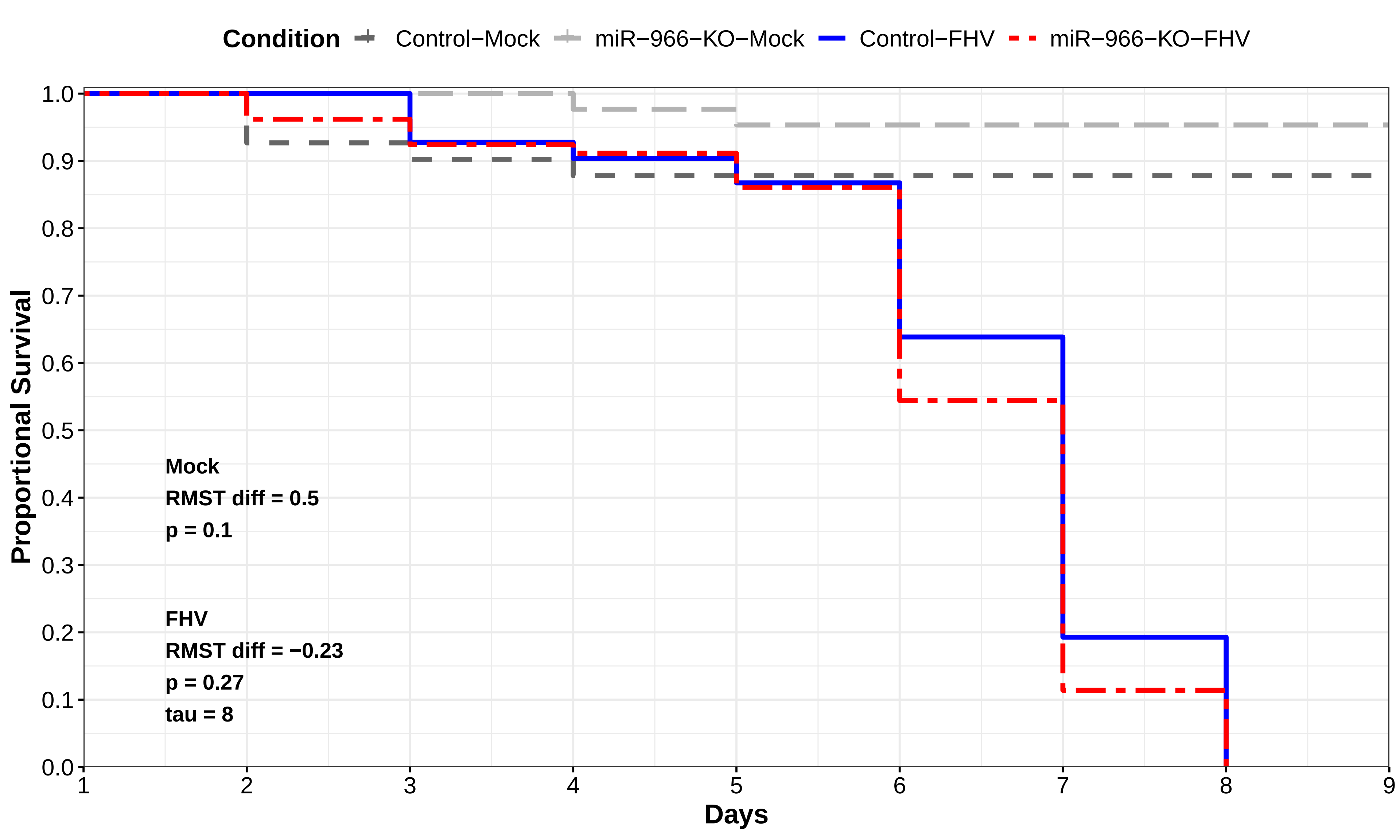

Condition
Control−Mock
miR−966−KO−Mock
Control−FHV
miR−966−KO−FHV
Proportional Survival
1.0
0.9
0.8
0.7
0.6
0.5
0.4
0.3
0.2
0.1
0.0
Mock
RMST diff = 0.5
p = 0.1
FHV
RMST diff = −0.23
p = 0.27
tau = 8
1
2
3
4
5
6
7
8
9
Days

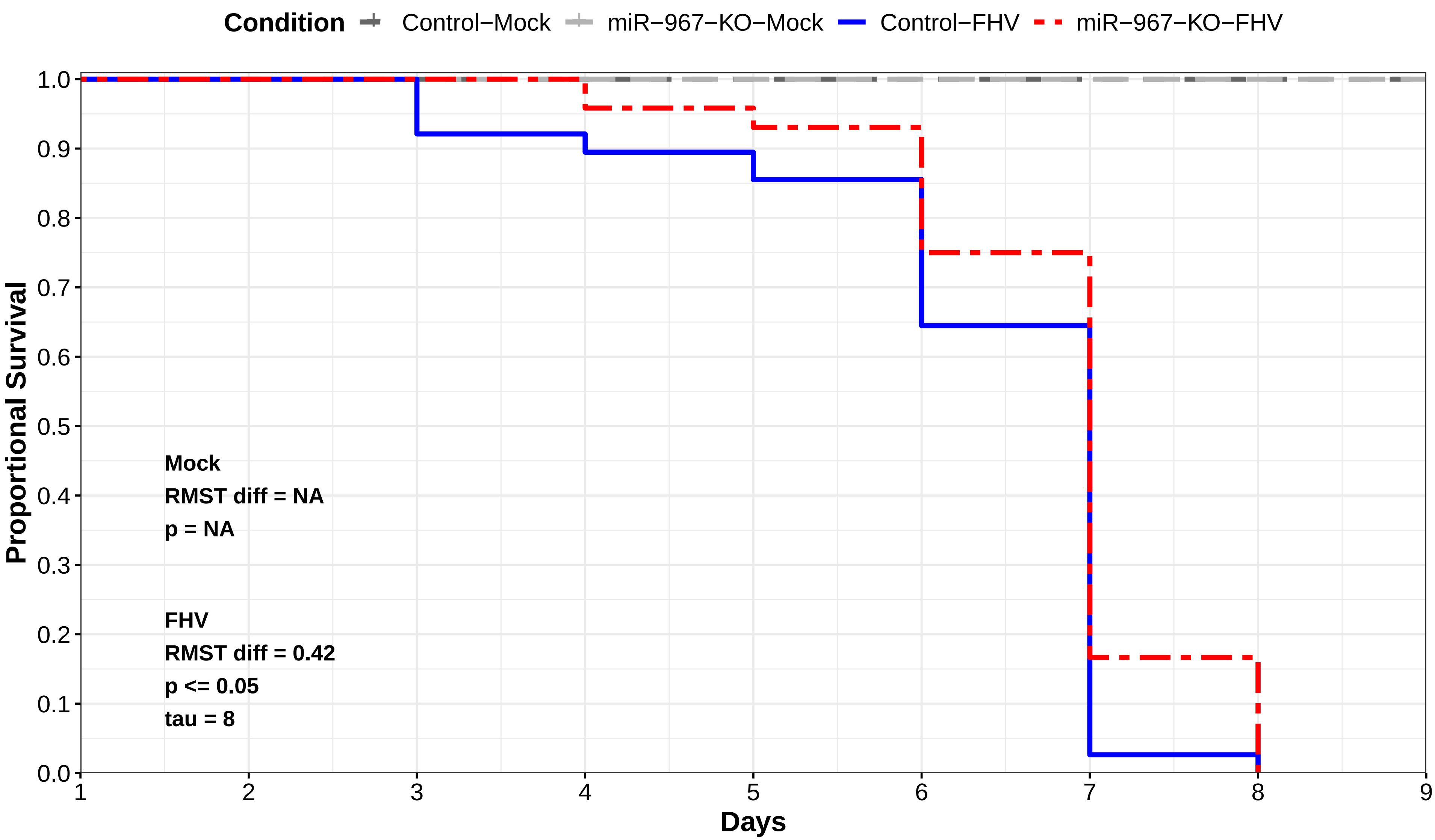

Condition
Control−Mock
miR−967−KO−Mock
Control−FHV
miR−967−KO−FHV
Proportional Survival
1.0
0.9
0.8
0.7
0.6
0.5
0.4
0.3
0.2
0.1
0.0
Mock
RMST diff = NA
p = NA
FHV
RMST diff = 0.42
p <= 0.05
tau = 8
1
2
3
4
5
6
7
8
9
Days

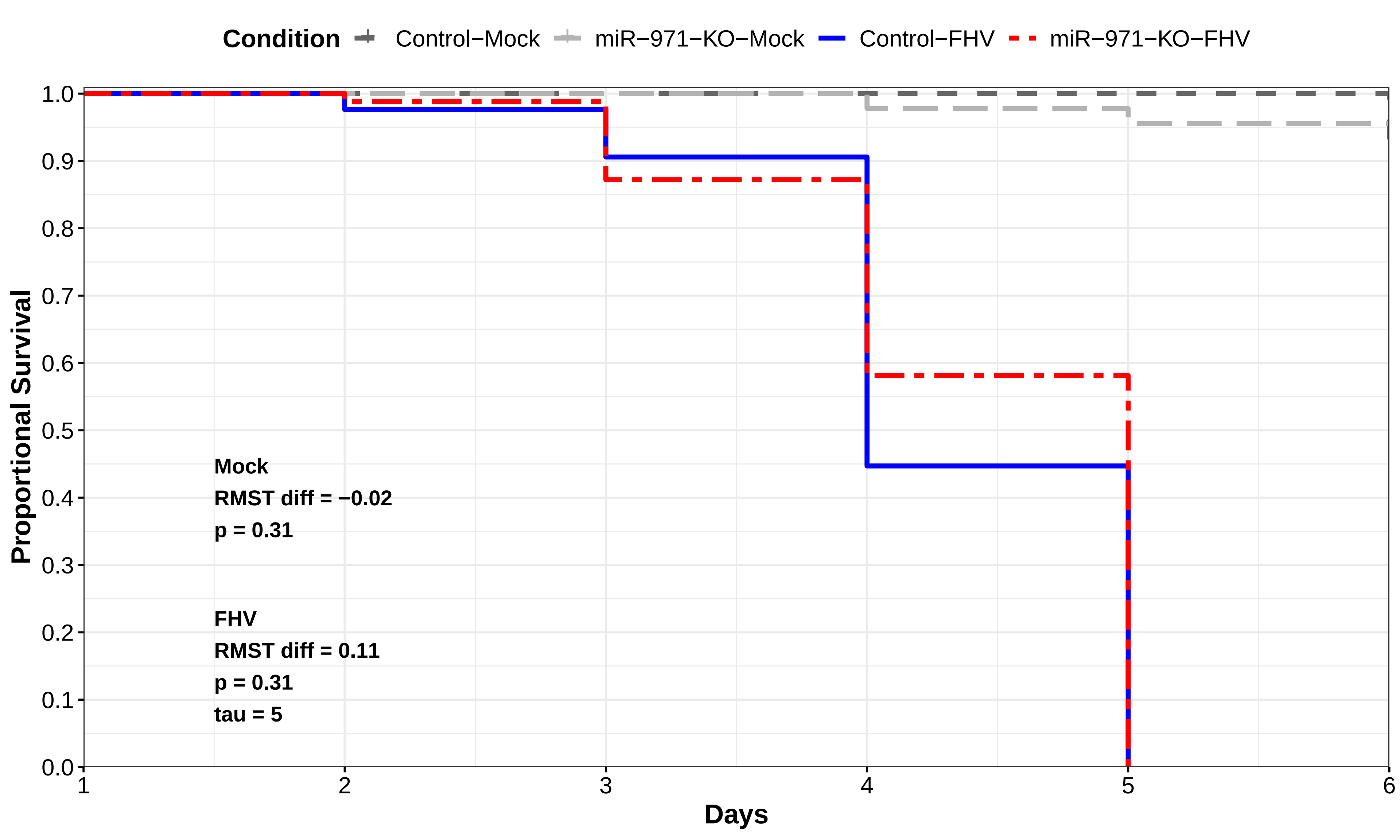

Condition
Control−Mock
miR−971−KO−Mock
Control−FHV
miR−971−KO−FHV
Proportional Survival
1.0
0.9
0.8
0.7
0.6
0.5
0.4
0.3
0.2
0.1
0.0
Mock
RMST diff = −0.02
p = 0.31
FHV
RMST diff = 0.11
p = 0.31
tau = 5
1
2
3
4
5
6
Days

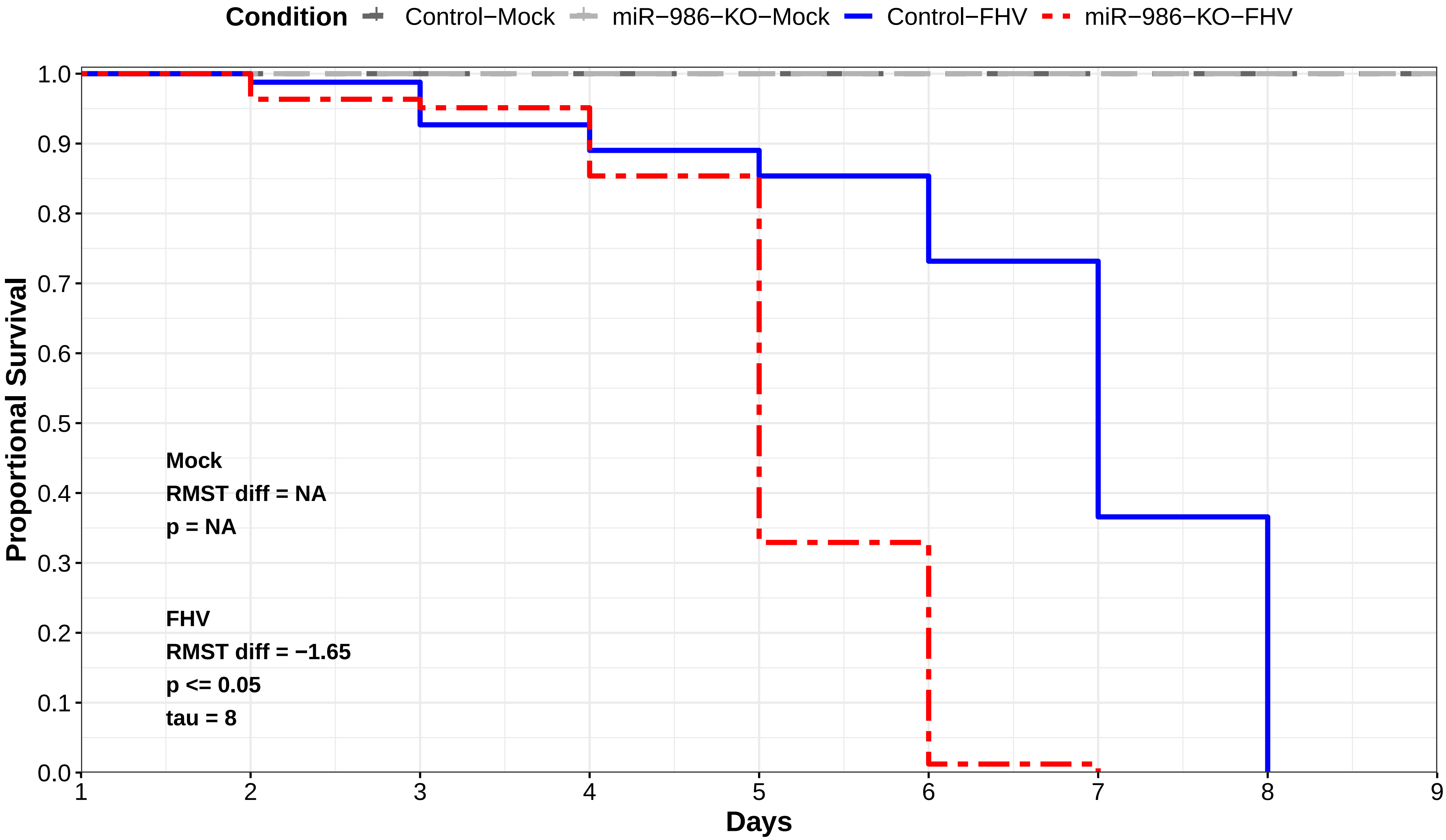

Condition
Control−Mock
miR−986−KO−Mock
Control−FHV
miR−986−KO−FHV
Proportional Survival
1.0
0.9
0.8
0.7
0.6
0.5
0.4
0.3
0.2
0.1
0.0
Mock
RMST diff = NA
p = NA
FHV
RMST diff = −1.65
p <= 0.05
tau = 8
1
2
3
4
5
6
7
8
9
Days

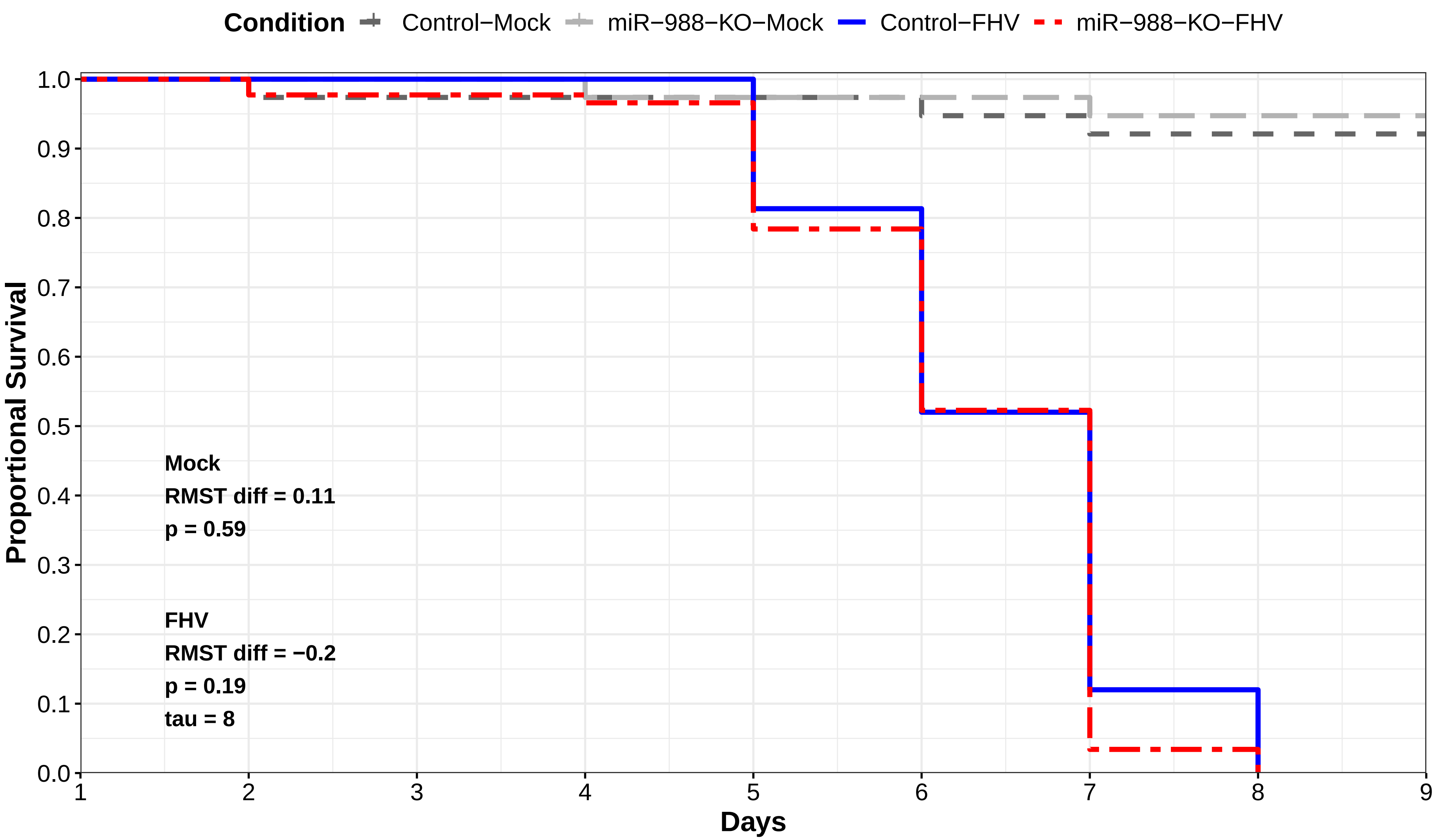
Condition
Control−Mock
miR−988−KO−Mock
Control−FHV
miR−988−KO−FHV
Proportional Survival
1.0
0.9
0.8
0.7
0.6
0.5
0.4
0.3
0.2
0.1
0.0
Mock
RMST diff = 0.11
p = 0.59
FHV
RMST diff = −0.2
p = 0.19
tau = 8
1
2
3
4
5
6
7
8
9
Days

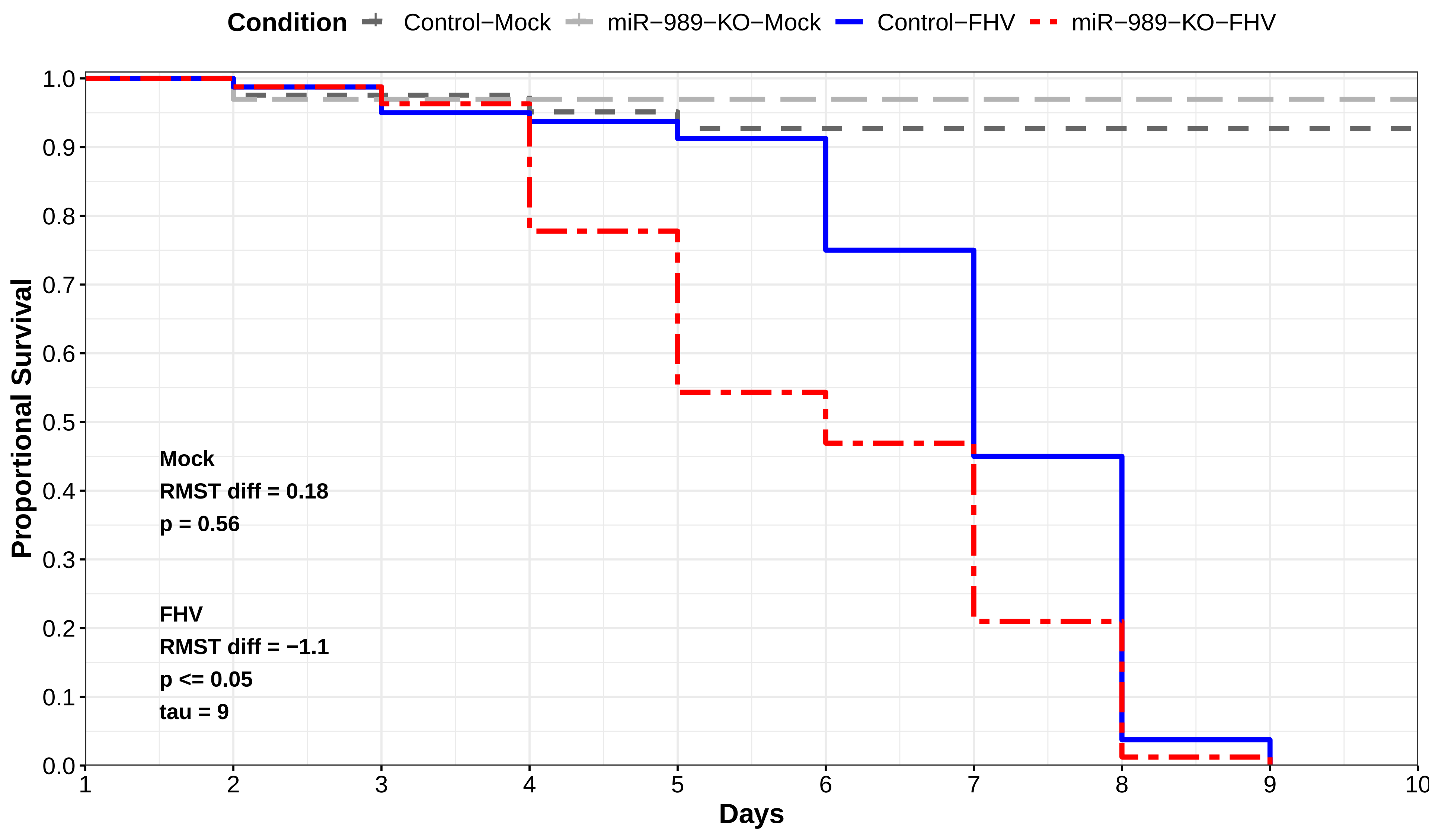

Condition
Control−Mock
miR−989−KO−Mock
Control−FHV
miR−989−KO−FHV
Proportional Survival
1.0
0.9
0.8
0.7
0.6
0.5
0.4
0.3
0.2
0.1
0.0
Days
1
2
3
4
5
6
7
8
9
10
Mock
RMST diff = 0.18
p = 0.56
FHV
RMST diff = −1.1
p <= 0.05
tau = 9

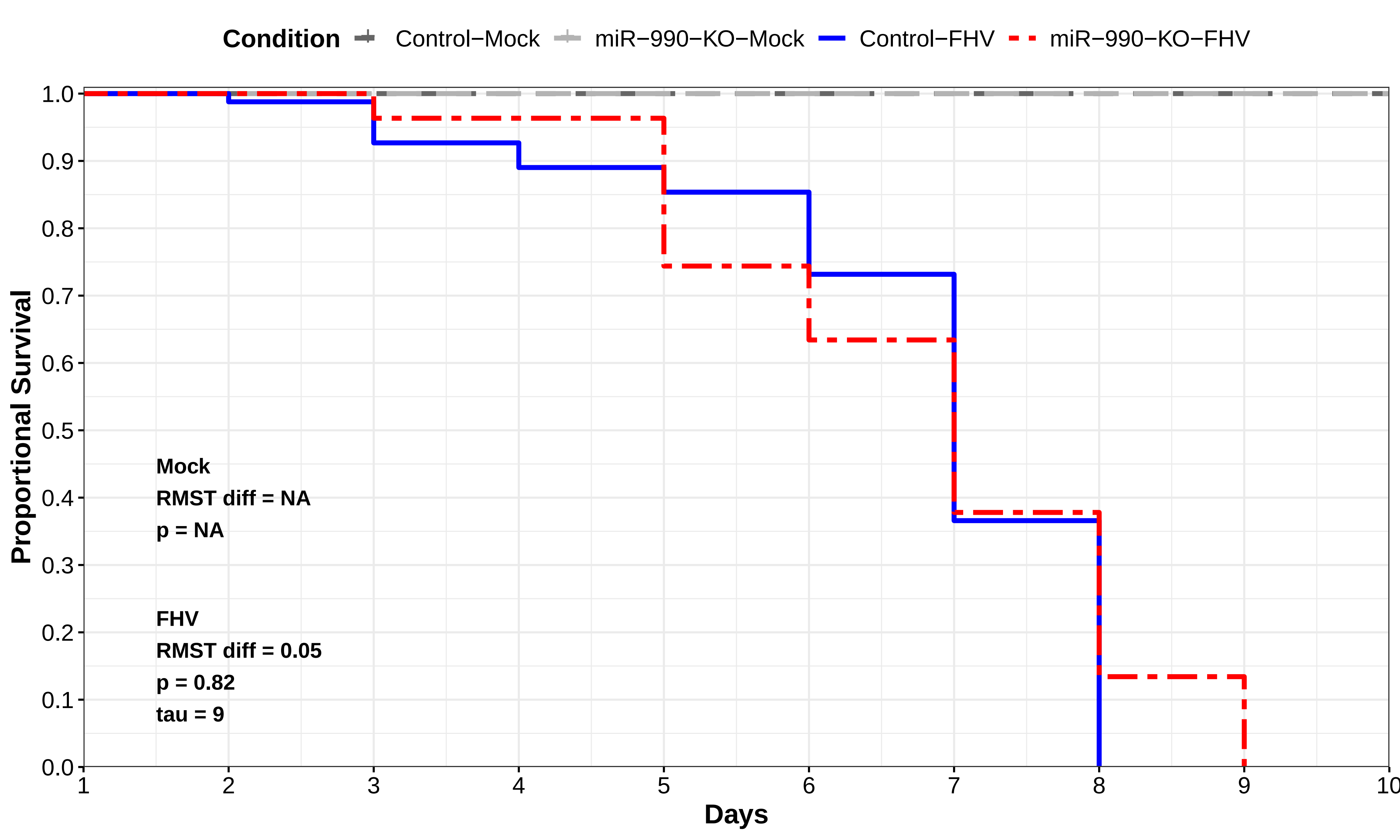

Condition
Control−Mock
miR−990−KO−Mock
Control−FHV
miR−990−KO−FHV
Proportional Survival
1.0
0.9
0.8
0.7
0.6
0.5
0.4
0.3
0.2
0.1
0.0
Mock
RMST diff = NA
p = NA
FHV
RMST diff = 0.05
p = 0.82
tau = 9
1
2
3
4
5
6
7
8
9
10
Days

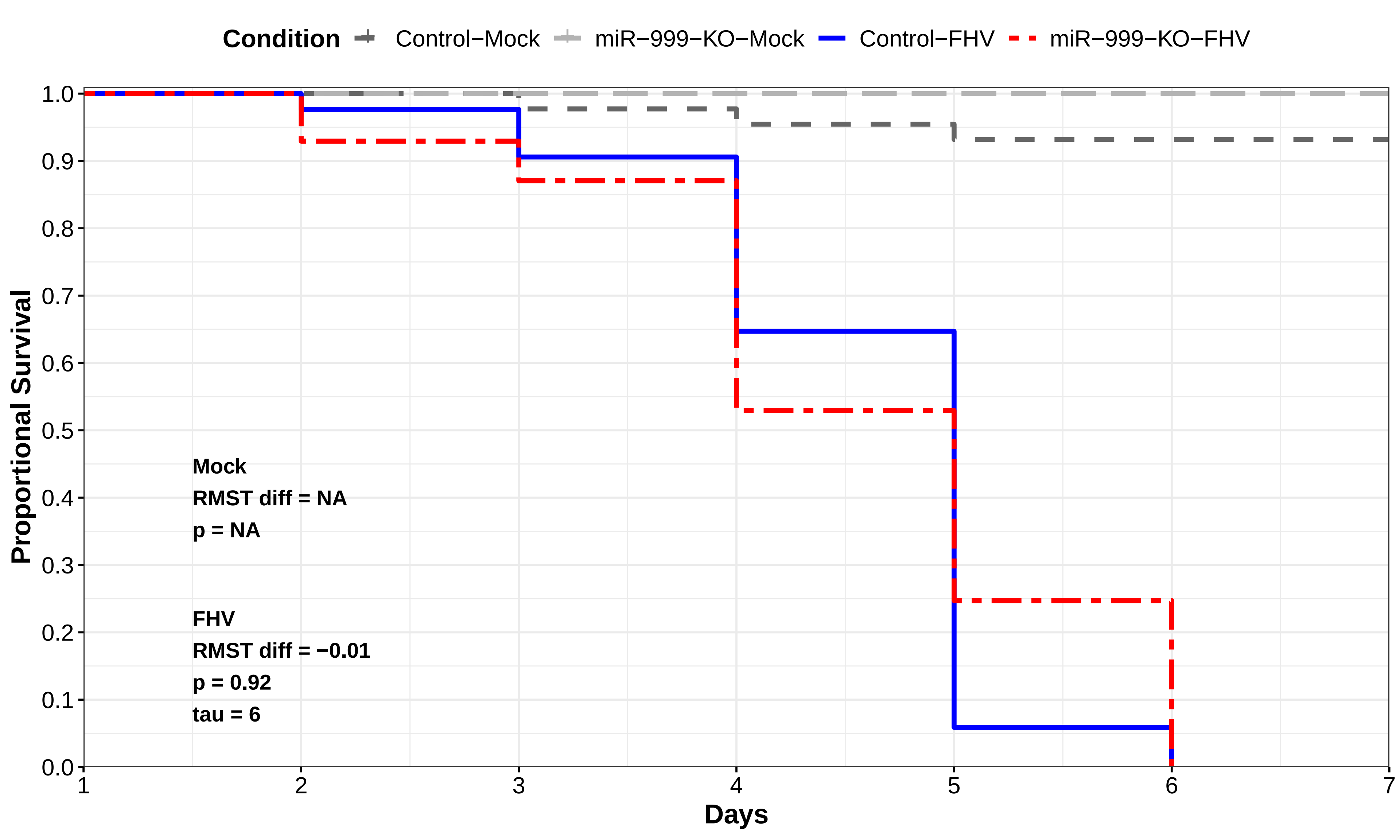
Condition
Control−Mock
miR−999−KO−Mock
Control−FHV
miR−999−KO−FHV
Proportional Survival
1.0
0.9
0.8
0.7
0.6
0.5
0.4
0.3
0.2
0.1
0.0
1
2
3
4
5
6
7
Days
Mock
RMST diff = NA
p = NA
FHV
RMST diff = −0.01
p = 0.92
tau = 6

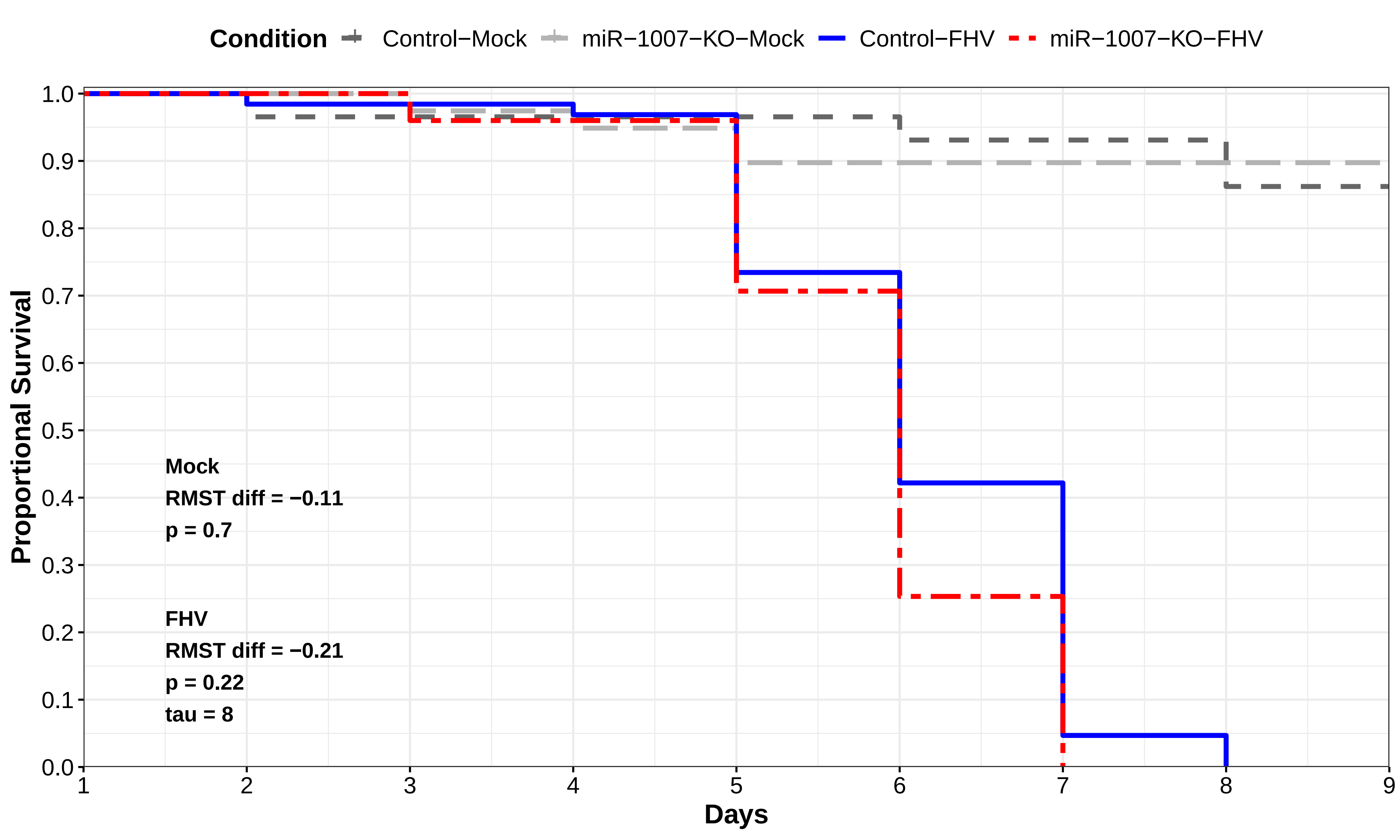
Condition
Control−Mock
miR−1007−KO−Mock
Control−FHV
miR−1007−KO−FHV
Proportional Survival
1.0
0.9
0.8
0.7
0.6
0.5
0.4
0.3
0.2
0.1
0.0
Mock
RMST diff = −0.11
p = 0.7
FHV
RMST diff = −0.21
p = 0.22
tau = 8
1
2
3
4
5
6
7
8
9
Days

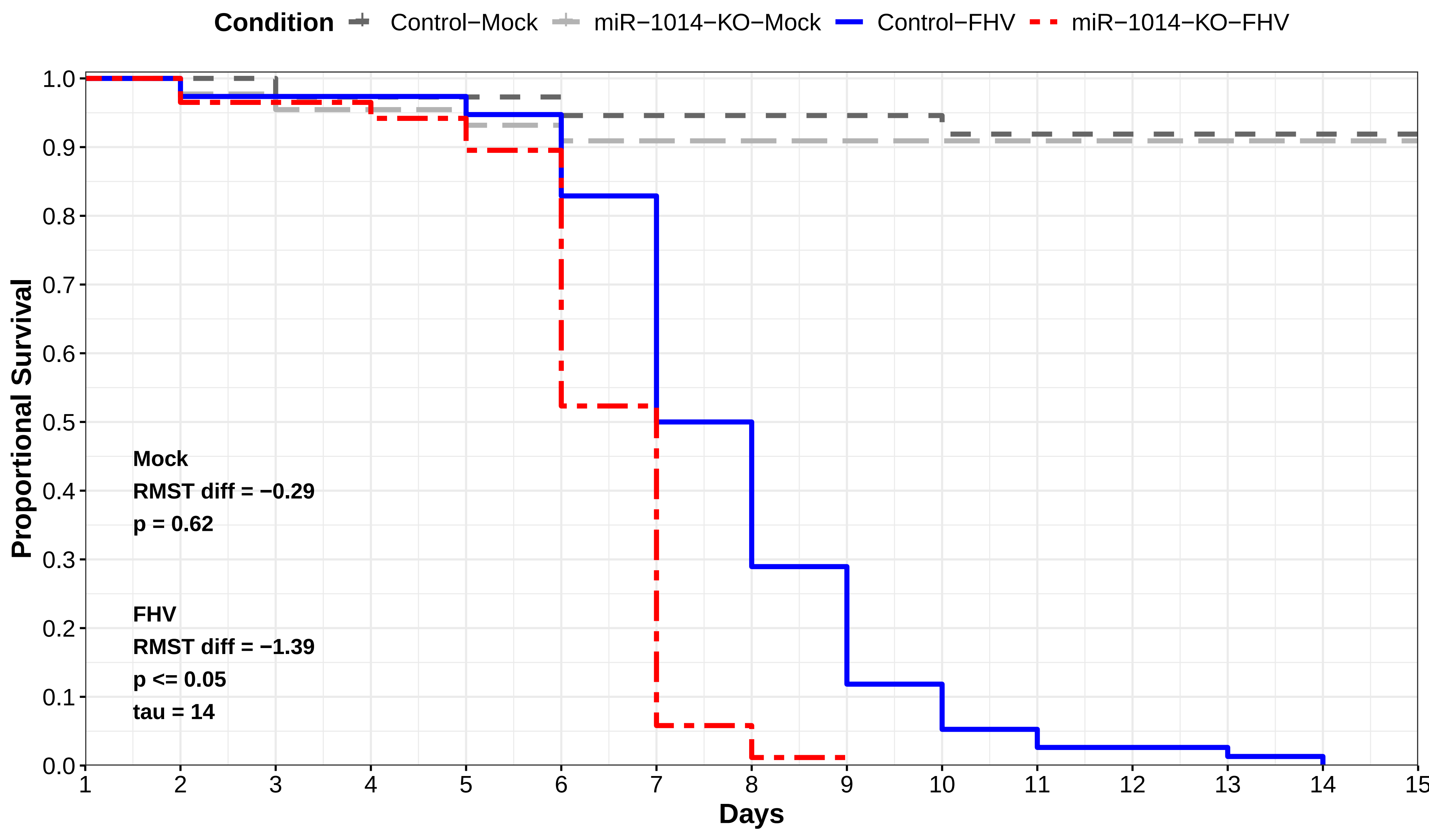

Condition
Control−Mock
miR−1014−KO−Mock
Control−FHV
miR−1014−KO−FHV
Proportional Survival
1.0
0.9
0.8
0.7
0.6
0.5
0.4
0.3
0.2
0.1
0.0
Mock
RMST diff = −0.29
p = 0.62
FHV
RMST diff = −1.39
p <= 0.05
tau = 14
1
2
3
4
5
6
7
8
9
10
11
12
13
14
15
Days

Figure S2: The impact of miRNA dysregulation on the outcome of FHV infection. A panel of 34 miRNA loss of function mutants were independently injected with FHV (10^8 IU/mL). Survival was monitored daily for each mutant (red, dash-dot) and compared to a paired control (blue, solid). Mock-injected mortality is also included for each line: mutant (light grey, long-dash) and control (dark grey, short-dash). Mortality occurring within the first 24 h was considered needle-stick injury and censored. Statistical analyses were conducted using Restricted Mean Survival Time (RMST) in R (v4.5.1). In this context, RMST difference is the approximate difference in survival between groups. A negative RMST difference can be interpreted as days decrease in survival, while a positive RMST difference as days increase in survival versus control (e.g. miR-31b has a RMST difference of 3.1, thus on average flies of the miR-31b loss of function line lived 3.1 days longer than control). Truncation time (tau) for both analyses was identified as the higher of the maximum survival times between FHV-injected mutant and control. A p-value < 0.05 indicates a statistically significant difference between the mutant line and control. For the FHV-injected populations, biological replicate was included as a covariate. Three independent biological replicates were performed for each line. Presented survival curves are the composite of all replicates.
