# Supplementary material for: Dysregulation of miRNAs has broad impacts on virus infection in Drosophila
Source: J Virol. 2026 Jul 2;100(7):e00850-26. doi: 10.1128/jvi.00850-26 (PMC13386962; doi:10.1128/jvi.00850-26)
Supplement: Table S8 — R packages used in this study. [file jvi.00850-26-s0006.docx]

**Table S8: R Packages used in this Study.**

| **Package** | **REF** |
| --- | --- |
| survRM2 | (1) |
| survival | (2) |
| broom | (3) |
| ggplot2 | (4) |
| purrr | (5) |
| dplyr | (6) |
| survminer | (7) |
| Biostrings | (8) |
| tidyr | (9) |
| MASS | (10) |

**References**

1. Uno H, Tian L, Horiguchi M, Cronin A, Battioui C, Bell J. 2022. survRM2: Comparing restricted mean survival time. CRAN Repos.

2. Therneau TM, Lumley T, Atkinson E, Crowson C. 2026. survival: Survival analysis. CRAN Repos.

3. Robinson D, Hayes A, Couch S, Hvitfeldt E. 2025. broom: Convert statistical objects into tidy tibbles. CRAN Repos 1.0.11.

4. Wickham H, Chang W, Henry L, Pedersen TL, Takahashi K, Wilke C, Woo K, Yutani H, Dunnington D, Van Den Brand T. 2025. ggplot2: Create elegant data visualisations using the grammar of graphics. CRAN Repos.

5. Wickham H, Henry L. 2026. purrr: Functional programming tools. CRAN Repos.

6. Wickham H, François R, Henry L, Müller K, Vaughan D. 2023. dplyr: A grammar of data manipulation. CRAN Repos.

7. Kassambara A, Kosinski M, Biecek P. 2025. survminer: Drawing survival curves using “ggplot2.” CRAN Repos.

8. H. Pagès PA. 2017. Biostrings: Efficient manipulation of biological strings. Bioconductor.

9. Wickham H, Vaughan D, Girlich M. 2025. tidyr: Tidy messy data. CRAN Repos.

10. Ripley B, Venables B. 2025. MASS: Support functions and datasets for Venables and Ripley’s MASS. CRAN Repos.
